# Structure revision of cryptosporioptides and determination of the genetic basis for dimeric xanthone biosynthesis in fungi†
†Electronic supplementary information (ESI) available. See DOI: 10.1039/c8sc05126g


**DOI:** 10.1039/c8sc05126g

**Published:** 2019-01-21

**Authors:** Claudio Greco, Kate de Mattos-Shipley, Andrew M. Bailey, Nicholas P. Mulholland, Jason L. Vincent, Christine L. Willis, Russell J. Cox, Thomas J. Simpson

**Affiliations:** a School of Chemistry , University of Bristol , Cantock's Close , Bristol , UK BS8 1TS . Email: russell.cox@oci.uni-hannover.de ; Email: tom.simpson@bristol.ac.uk; b School of Biological Sciences , 24 Tyndall Avenue , Bristol , BS8 1TQ , UK; c Syngenta , Jealott's Hill International Research Centre , Bracknell , RG42 6EY , UK; d Institute for Organic Chemistry , Leibniz University of Hannover , Schneiderberg 1B , 30167 , Hannover , Germany; e BMWZ , Leibniz University of Hannover , Schneiderberg 38 , 30167 , Hannover , Germany

## Abstract

Three novel dimeric xanthones, cryptosporioptides A–C were isolated from *Cryptosporiopsis* sp. 8999 and their structures elucidated. Methylation of cryptosporioptide A gave a methyl ester with identical NMR data to cryptosporioptide, a compound previously reported to have been isolated from the same fungus. However, HRMS analysis revealed that cryptosporioptide is a symmetrical dimer, not a monomer as previously proposed, and the revised structure was elucidated by extensive NMR analysis. The genome of *Cryptosporiopsis* sp. 8999 was sequenced and the dimeric xanthone (*dmx*) biosynthetic gene cluster responsible for the production of the cryptosporioptides was identified. Gene disruption experiments identified a gene (*dmxR5*) encoding a cytochrome P450 oxygenase as being responsible for the dimerisation step late in the biosynthetic pathway. Disruption of *dmxR5* led to the isolation of novel monomeric xanthones. Cryptosporioptide B and C feature an unusual ethylmalonate subunit: a hrPKS and acyl CoA carboxylase are responsible for its formation. Bioinformatic analysis of the genomes of several fungi producing related xanthones, *e.g.* the widely occurring ergochromes, and related metabolites allows detailed annotation of the biosynthetic genes, and a rational overall biosynthetic scheme for the production of fungal dimeric xanthones to be proposed.

## Abstract 2

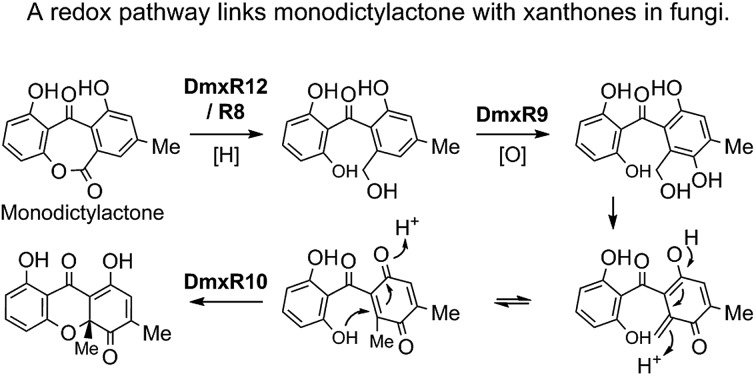
Three novel dimeric xanthones, cryptosporioptides A–C were isolated from *Cryptosporiopsis* sp. 8999 and their structures elucidated.

## Introduction

**Fig. 1:**
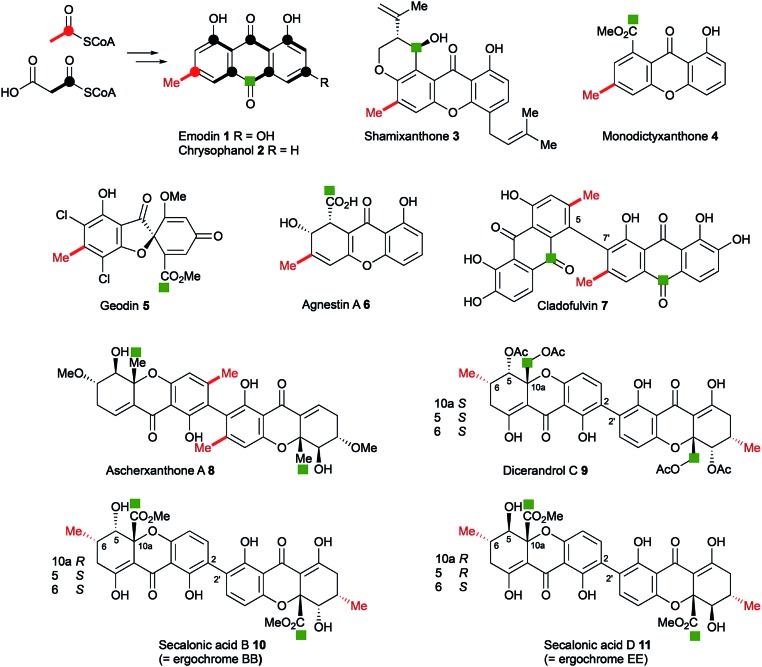
Typical fungal anthraquinone and xanthone metabolites. Isotope labelling pattern is shown for emodin/chrysophanol with the polyketide starter unit indicated in red. The green square labels (*e.g.* C-12 of the secalonic acids) indicate equivalent carbons derived from the C-10 carbonyl of emodin/chrysophanol.

Anthraquinones and xanthones are among the most common and earliest discovered fungal secondary metabolites,^1^ and they often have interesting and useful bioactivities. These compounds are polyketides, the skeletons of which are produced from acetyl and malonyl CoA by non-reducing polyketide synthases (nr-PKS).^2^ Examples include: the anthraquinones emodin **1** and its reduced congener chrysophanol **2** which is a precursor of shamixanthone **3**,^3^ and monodictyxanthone **4**^4^ from *Aspergillus nidulans*.^5^,^6^ Emodin **1** is a precursor of the antifungal agent geodin **5**,^7^ which while not strictly a xanthone, belongs to the same biosynthetic family. We have also recently isolated other xanthones such as agnestin A **6** from *Paecilomyces variotii* and linked it to its biosynthetic genes.^8^ Cladofulvin **7**^9^ from the tomato pathogen *Cladosporium fulvum* is a dimeric anthraquinone also derived from **2**. Dimeric xanthones are also known, and they often have varied biological activities.^10^ They include the antimalarial ascherxanthone A **8**;^11^ the antimicrobial and anticancer dicerandrol C **9**;^12^ and the mycotoxins secalonic acids B **10** and D **11** which are also known as ergochromes (Fig. 1).^13^ Although many of the dimeric xanthones, particularly the widespread secalonic acid family, have been studied for many years,^14^ key aspects of their biosynthesis such as the mechanisms and timing of the dimerisation process, and isolation and analysis of their biosynthetic gene clusters remain largely unknown.

**Fig. 2:**
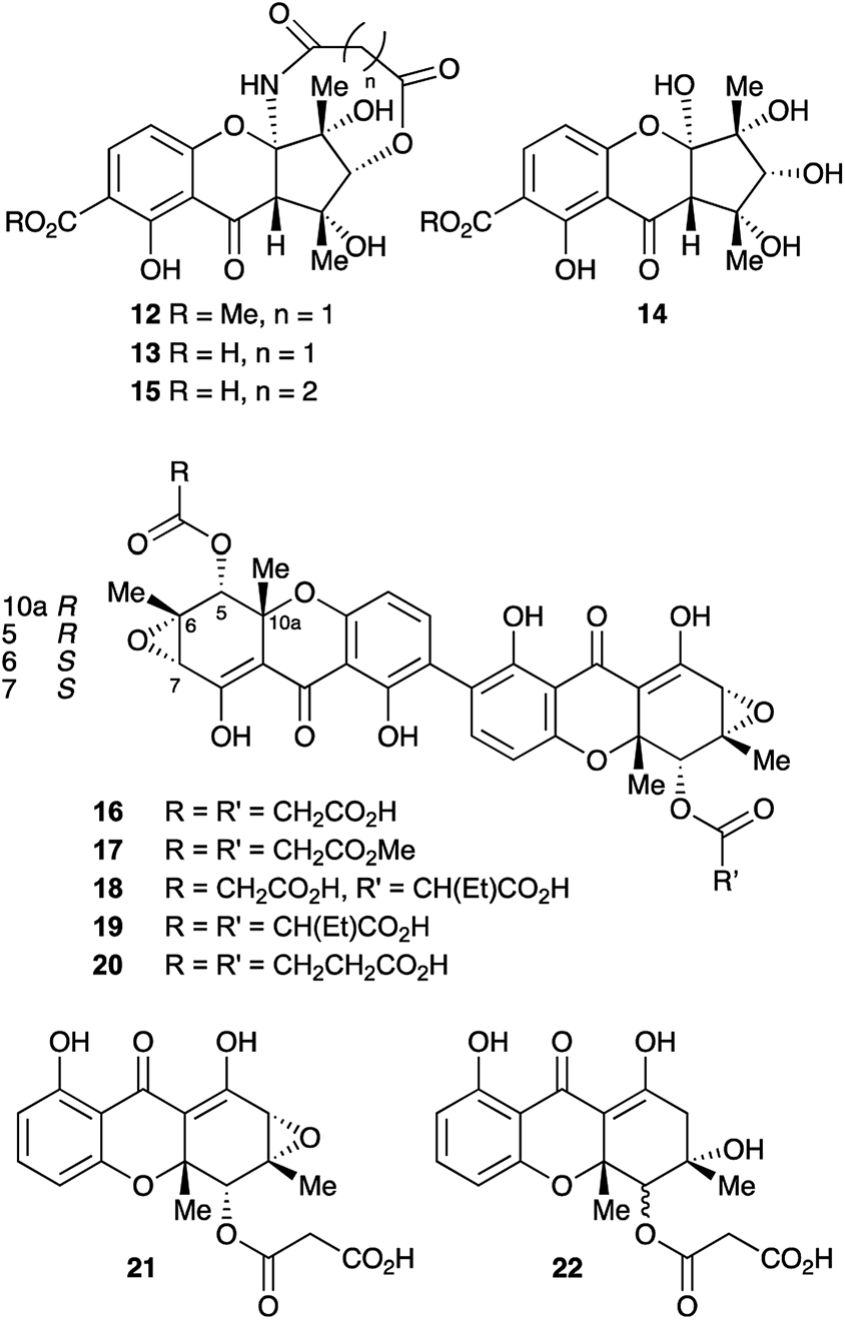
Previous (**12–15**) and reassigned (**16–20**) structures of the cryptosporioptides.

In the course of our ongoing studies on fungal maleidride biosynthesis^15^ we analysed fermentations of the endophytic fungus *Cryptosporiopsis* sp. 8999, which produces the unusual octadride, viburspiran.^16^ This strain was also reported^17^ to produce a metabolite named cryptosporioptide, which was assigned the monomeric xanthone methyl ester structure **12** (Fig. 2). This structure contains an unusual ring-contracted xanthone which is difficult to rationalise biosynthetically, and an unprecedented *N*-malonyl aminal bridge. Further studies^18^ of this strain also led to the isolation of the corresponding free acid, named cryptosporioptide A **13**, and cryptosporioptide B **14**, which lacks the malonic acid ester amide bridge.

Subsequently, isolation of an analogue **15**, in which the malonate bridge has been replaced by a succinate, was reported from the insect parasite *Cordyceps gracilloides*.^19^ Confusingly, this was also named cryptosporioptide A.

The structure of the parent cryptosporioptide **12** was assigned on the basis of FAB-HRMS which indicated a molecular formula C_19_H_19_NO_10_. Detailed analysis of 1D and 2D ^1^H and ^13^C NMR spectra gave the connectivities and relative stereochemistry on which the structures were assigned. The absolute configuration was assigned by circular dichroism allied to time-dependent density functional theory (TDDFT) computational procedures.^11^ The related structures **13** and **15** were assigned on the basis of the similarity of their NMR spectra and optical rotations to those originally reported for cryptosporioptide.

We now report the isolation and structure elucidation of a novel dimeric tetrahydroxanthone metabolite **16**, methylation of which gives a compound with identical NMR properties to those reported^17^ for cryptosporioptide, along with related dimeric structures, **17–19** (Fig. 2). Following sequencing of the biosynthetic gene cluster and targeted gene knock-outs, the monomeric structures, **21** and **22** were also isolated. Comparison with genome sequences of fungi known to produce xanthones and related structures, allows identification and annotation of the biosynthetic gene clusters (BCGs) for several monomeric and dimeric xanthones, *inter alia* the secalonic acids.

## Results

*Cryptosporiopsis* sp. 8999 was grown on a range of different media. Three related metabolites were produced in good yields when the fungus was grown on brown rice (*e.g.*Fig. 4B). The major metabolite (900 mg kg^–1^) had almost identical IR, UV and ^1^H and ^13^C NMR data apart from the lack of a methoxy group when compared to that reported for cryptosporioptide A **13**.^17^ When esterified using TMS-diazomethane, the resulting methyl ester had identical NMR data to those reported for cryptosporioptide **12** by Saleem *et al.*,^17^ for which the reported FAB-HRMS data was 444.0781 [M + Na]^+^ (C_19_H_19_NO_10_Na).^17^ However, when the molecular weight for the derivatised compound was redetermined using FAB-HRMS and ESI-HRMS, we obtained HRMS of 801.1506 [M + Na]^+^, and 778.1828 [M]^+^ respectively, both consistent with a molecular formula C_38_H_34_O_18_, and notably lacking nitrogen. The ^1^H NMR spectrum showed 9 hydrogen environments, but 19 different carbon environments were observed in the ^13^C NMR spectrum consistent with a symmetrical dimer.

**Fig. 3:**
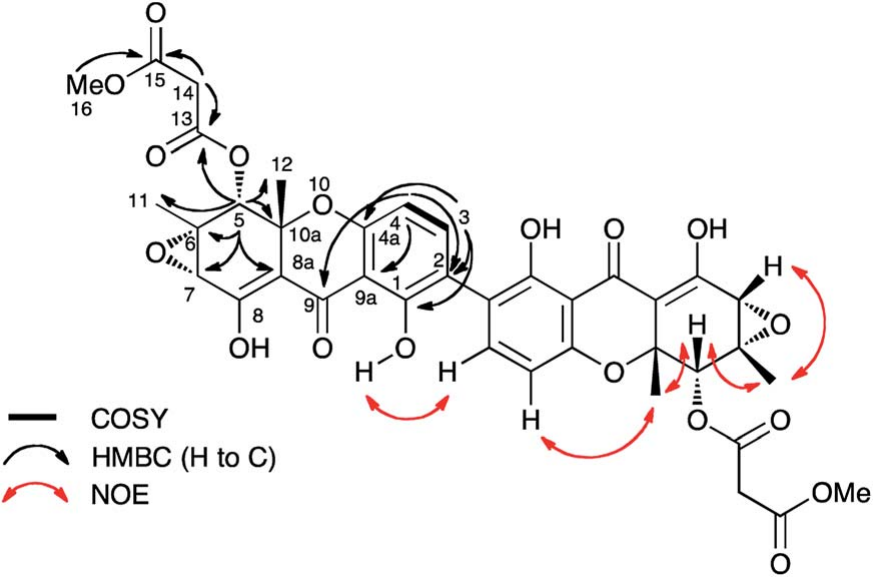
Selected correlations observed in 2D NMR spectra of cryptosporioptide A dimethyl ester **17**. Secalonic acid numbering used. See Table S7 (ESI†) for full details.

The presence of a tetrasubstituted aromatic ring was indicated by *ortho*-coupled hydrogens (*δ*_H_ 7.70 and 6.43 ppm, *J* = 8.5 Hz) in the ^1^H NMR spectrum, which also showed two methyl singlets (*δ*_H_ 1.65 and 1.59 ppm), two oxygen bearing methine singlets (*δ*_H_ 3.42 and 5.64 ppm), two low field exchangeable hydrogens (*δ*_H_ 11.69 and 14.02 ppm) and geminally coupled methylenes (*δ*_H_ 3.39 and 3.44 ppm, *J* = 15.8 Hz). The ^13^C NMR spectrum also showed other signals attributable to a phenolic ring (*δ*_C_ 159.2, 157.2, 140.6 and 106.1 ppm), four ester/enolic carbons (*δ*_C_ 165.5, 166.7, 170.0 and 104.4 ppm) and a benzophenone carbonyl (*δ*_C_ 187.9 ppm). Signals at *δ*_C_ 56.0, 58.8, 73.9 and 78.9 ppm are consistent with the presence of an epoxide, secondary alcohol and ether. The connectivities were determined from extensive HMBC correlations which confirmed the presence of the aromatic ring, and a highly substituted cyclohexene (Fig. 3), the key signal being H-5 (5.64 ppm) which correlates with a total of eight carbons (6, 7, 8a, 9, 10a, 11, 12 and 13 at *δ*_C_ 58.8, 56.0, 170.0, 187.9, 78.9, 28.6, 18.2 and 165.5 ppm respectively). The H-5/C-13 correlation establishes the position of attachment of the malonate moiety, the other malonate carbonyl (C-15) correlating with the methoxyl (*δ*_H_ 3.65, *δ*_C_ 52.7 ppm). The connectivities between the two carbocyclic rings were confirmed by observation of HMBC correlations (see ESI Fig. S15†) from both H-5 (5.64 ppm) and H-4 (6.43 ppm) to the C-9 ketone (*δ*_C_ 187.9 ppm), and nOes between the 12-methyl and H-4 in the aromatic ring. The relative stereochemistry C-5 to C-7 and C-11 was established by nOes between H-5 and H-7 and the 11- and 12-methyls.

While the absolute configuration of the original cryptosporioptide structure was established by comparison of the measured circular dichroism (CD) with that predicted by computational methods, the complete revision of the structure renders this assignment invalid, and indeed suggests caution should be exercised when applying these methods which are being increasingly used for configurational assignment. The absolute configuration at the C-10a stereogenic centres of secalonic acid B **10** and related structures, *e.g.* dicerandrol C **9** have been assigned on the basis of a positive n–π* CD band at 330–340 nm as 10a*R* and 10a*S* respectively (note change in CIP designation due to priority inversion for CO_2_Me and CH_2_OAc).^20^–^22^ The absolute configuration of dicerandrol C **9** has also been confirmed by total synthesis.^23^ Cryptosporioptide A dimethyl ester **17** has a reported CD (341 nm, Δ*ε* = + 5.0)^17^ which is consistent with the 10a*R* configuration. Thus cryptosprioptide can be designated as 5*R*,6*S*,7*S*,10a*R*.

Finally, the connectivity between the individual xanthone monomers was established by an intra-dimer nOe between 1-OH and H-3. The point of dimerisation was confirmed by isolation of monomers **21** and **22** following KO of DmxR5, the cytochrome P450 responsible for oxidative coupling (see below). Thus the previously reported structures **12**, and **13** have been revised to **17** and **16** respectively, and we propose renaming them cryptosporioptide A dimethyl ester, and cryptosporioptide A respectively. We have not observed any trace of the methyl ester **17** in any of our extracts, and the reported isolation of **17** as a natural product is possibly an artefact of the purification involved (Sephadex LH20 eluted with methanol).

The remaining two dimeric metabolites, cryptosporioptides B **18** and C **19**, differ from cryptosporioptide A **16** in the malonyl subunit, their UV and NMR spectra being otherwise identical (see ESI Tables S1 and S2†). HRMS showed their molecular formulae to be C_38_H_34_O_18_ and C_40_H_38_O_18_ respectively. The NMR spectra showed the latter **19** to be also essentially symmetrical with signals attributable to the ethylmalonate moieties (*δ*_H_ 3.29, 2H, m; 1.81, 4H, m; and 0.85, 6H, m). Doubling of some signals was observed, probably due to facile epimerisation of the ethylmalonyl substituent, giving diastereomers at C-13. The spectra for **18** are more complex with many signals doubled due to the presence of only one ethylmalonate moiety removing the symmetry. Again on the basis of the close similarities of the NMR spectra, the *C. gracilloides* metabolite^19^**15** has been renamed as cryptosporioptide D **20**.

While the biosynthetic pathways towards monomeric xanthones and related compounds such as geodin **5** have been investigated previously, there is much less information on the dimeric systems despite these having been the subject of intensive study over many years.^10^ The main question is the timing of ring cleavage to give the xanthone moiety relative to dimerisation. The recently reported^20^ co-occurrence of the monomeric blennolides (*e.g.* ergochrome B) with their symmetrical dimer, secalonic acid B **10** supports, but does not unequivocally prove that during secalonic acid biosynthesis, xanthone formation precedes dimerisation. In those systems where the anthraquinonoid carbonyl (*i.e.* C-10) is retained, it is often found as a methyl ester as in the secalonic acids (note C-12 in SA numbering), or partially reduced to a hydroxymethyl equivalent, *e.g.* in dicerandrol C **9**, or fully reduced to a methyl group as in the ascherxanthones, *e.g.***8** and cryptosporioptides **16–20**. Other questions include: which of the original anthraquinone rings retains the cleaved carbonyl C-10 (C-12 in SA nomenclature) – that containing the polyketide starter unit as in secalonic acids, *e.g.***10** and cryptosporioptides or the non-starter ring as in, *e.g.* ascherxanthones **8**; and the location of the site of dimerisation (Fig. 1).

**Fig. 4:**
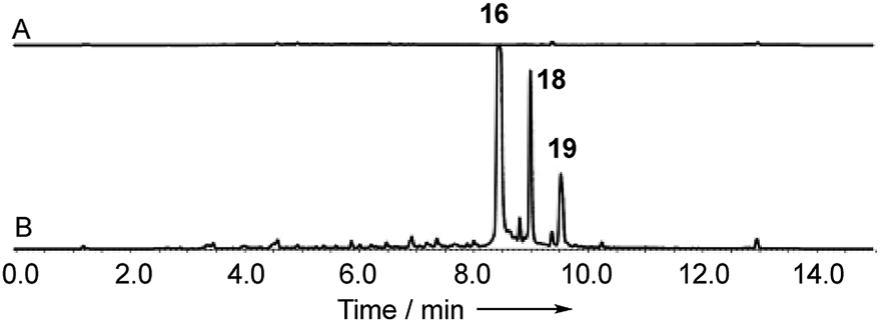
Knockout of *dmxpks1*. (A) LCMS analysis (DAD) of organic extract of *Cryptosporiopsis* sp. 8999 Δ*dmxpks1* strain fermented on brown rice; (B) LCMS analysis (DAD) of organic extract of wild type *Cryptosporiopsis* sp. 8999 fermented on brown rice. LCMS method 50–90% CH_3_CN:H_2_O gradient, 15 min.

With these questions in mind, the 54 Mbp draft genome of *Cryptosporiopsis* sp. 8999 was obtained using Illumina MiSeq and assembled and annotated using Newbler v29. The total number of contigs was 2166 with an N50 of 204 Kb. Bioinformatic studies using antiSMASH^24^ identified ten BGCs containing non-reducing polyketide synthase (nr-PKS) genes. A putative gene cluster for xanthone biosynthesis^25^ was readily identified by BLAST analysis using protein sequences from: the shamixanthone **3**/monodictyphenone **4** BGC from *Aspergillus nidulans*;^5^,^6^,^26^ the agnestin **6** BGC from *Paecilomyces variotii*;^8^ the recently (but partially, *vide infra*) described secalonic acid **10** BGC from *Claviceps purpurea*;^27^ the geodin **5** BGC from *Aspergillus terreus*;^7^ and the cladofulvin **7** BGC from *Cladosporium fulvum*.^9^ Development of a transformation system, targetted knockout of the putative cryptosporioptide (*dmx*) PKS using the bipartite method of Neilsen and coworkers,^28^ and subsequent observation of abolition of all cryptosporioptide production (Fig. 4) proved this assignment to be correct. We then used the cryptosporioptide BGC to screen the genomes of other fungi for similar clusters. *Penicillium oxalicum*^29^ and *Aspergillus aculeatus*^30^ are both known to produce secalonic acid D **11**,^31^ and their genomes have been sequenced.^32^ In both cases we found a BGC (*P. oxa*, *A. acu*Table 1) featuring homologs of many of the genes in the *dmx* BGC.

**Table 1:** The cryptosporioptide dimeric xanthone (*dmx*) cluster and similarities of encoded proteins with those of the monodictyphenone (*mdp*), geodin (*ged*), agnestin (*agn*), cladofulvin (*cla*) and secalonic acid (*sec*, *P. oxa* and *A. acu*) clusters

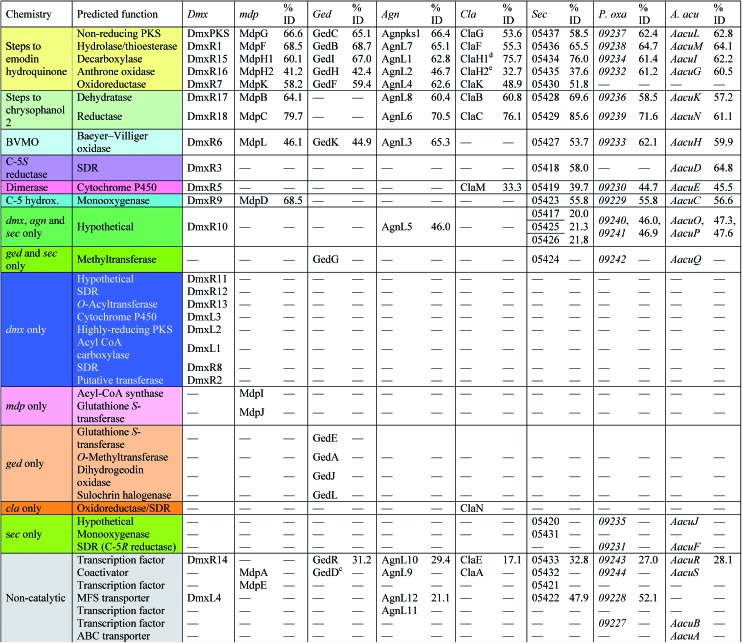

**Fig. 5:**
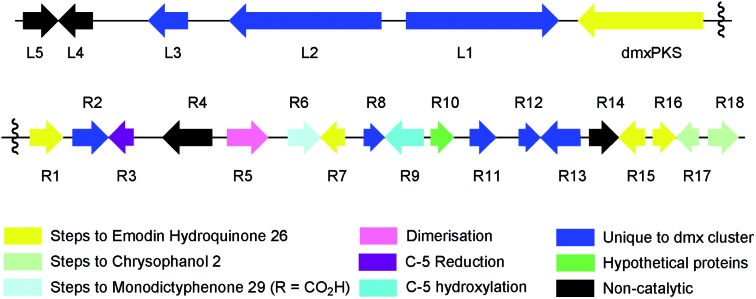
Map of the cryptosporioptide dimeric xanthone (*dmx*) BGC coloured by proposed function (see Table 1). Not to scale.

The *dmx* BGC spans approximately 84 Kb surrounding *dmxPKS* which encodes a fungal nr-PKS homologous to the monodictyphenone PKS (MdpG, 67% identity) from *A. nidulans*^6^ and the analogous polyketide synthases encoded by the geodin **5** (*ged*), agnestin **6** (*agn*), cladofulvin **7** (*cla*) and secalonic acid **10**, **11** (*sec*) clusters (Table 1, Fig. 5). Homologies to all genes which encode proteins required for the synthesis of emodin **1** (*dmxpks*, *dmxR1*, *dmxR15* and *dmxR16*) are present, as are the genes required for the synthesis of chrysophanol **2** (*dmxR7*, *dmxR17* and *dmxR18*). The geodin pathway, which does not proceed *via* chrysophanol **2**, lacks homologs of *dmxR17* and *dmxR18*.

Oxidative ring-opening of chrysophanol **2** is specified by *dmxR6* which encodes a Baeyer–Villiger monooxygenase (BVMO) homologous to those encoded by the *mdp*, *ged*, *agn* and *sec* clusters where this transformation is required. Notably cladofulvin, which is a dimeric anthraquinone, does not require this chemistry and the *cla* cluster lacks a *dmxR6* homolog. Beyond this point the content of the clusters varies significantly.

The *dmx* BGC has a series of unique genes not present in the other clusters such as *dmxR2*, *dmxR8*, *dmxR11-13* and *dmxL1-3*. In particular *dmxL2* encodes a highly reducing PKS (hr-PKS) homologous to the lovastatin diketide synthase^33^ and squalestatin tetraketide synthase.^34^ The gene *dmxL1* encodes an acyl-CoA carboxylase. The gene *dmxR5* encodes a cytochrome P450 enzyme homologous to ClaM which is known to dimerise nataloe-emodin to form cladofulvin **7**.^35^,^36^ This gene is missing from the clusters of the monomeric compounds, but is present in the *sec* cluster (CPUR_05419). Finally, *dmxR13* encodes an *O*-acyl transferase, and again this is absent from the other clusters.

**Fig. 6:**
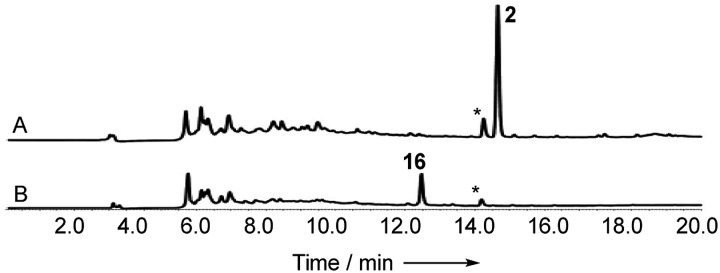
Knockout of *dmxR6* (BVMO). (A) LCMS analysis (DAD) of organic extract of *Cryptosporiopsis* sp. 8999 *ΔdmxR6* fermented on brown rice; (B) LCMS analysis (DAD) of organic extract of wild type *Cryptosporiopsis* sp. 8999 fermented on brown rice. LCMS method 50–90% CH_3_CN:H_2_O gradient, 20 min. * = unrelated compound.

**Fig. 7:**
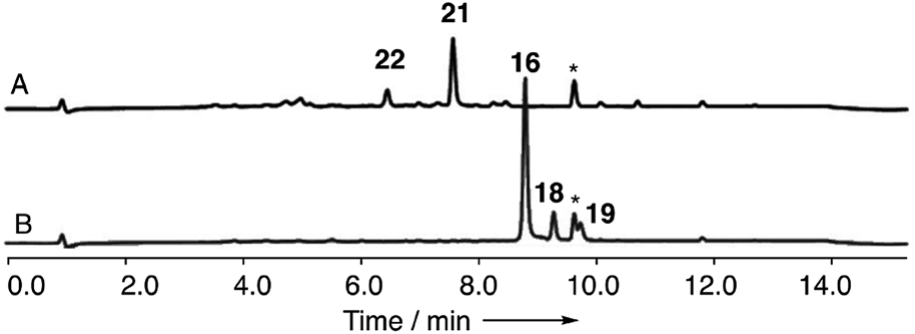
Knockout of *dmxR5* (P450). (A) LCMS analysis (DAD) of organic extract of *Cryptosporiopsis* sp. 8999 *ΔdmxR5* fermented on brown rice; (B) LCMS analysis (DAD) of organic extract of wild type *Cryptosporiopsis* sp. 8999 fermented on brown rice. LCMS method 50–90% CH_3_CN:H_2_O gradient, 15 min. * = unrelated compound.

In order to gain evidence for the function of these genes we devised knockout experiments. Disruption of *dmxR6* (BVMO) gave high titres of chrysophanol **2** as expected, showing that dimerisation occurs after anthraquinone ring cleavage and xanthone formation (Fig. 6). Deletion of *dmxR5* (putative dimerase) abolished dimer production and two novel metabolites with similar UV spectra to the cryptosporioptides but with molecular weights consistent with monomers were isolated (Fig. 7). Full NMR analysis (see ESI†) confirmed that these were hemi-cryptosporioptide **21** (C_19_H_16_O_9_), and an analogue **22** (C_19_H_18_O_9_) containing a tertiary alcohol at C-6 which was isolated as a mixture of epimers at C-5.

**Fig. 8:**
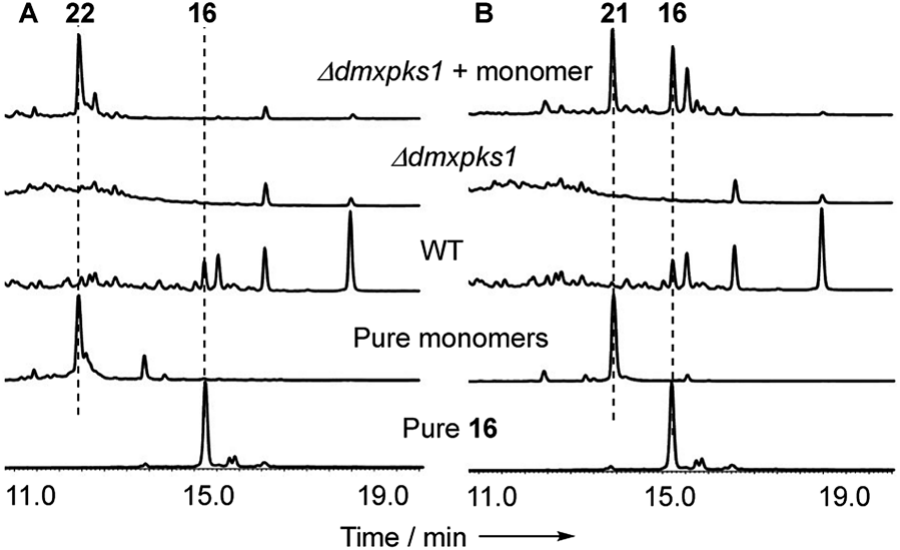
Experiments showing that **21** is a precursor of **16**: (A) feeding experiments using purified monomer **22**; (B) feeding experiments using purified monomer **21**. Fermentations were performed in liquid PDB media. LCMS method 10–90% CH_3_CN:H_2_O gradient, 20 min. DAD scans shown.

When monomer **21**, but not **22**, was re-fed to the Δ*dmxPKS* mutant, cryptosporioptide A **16** production was restored (Fig. 8), confirming this as a pathway intermediate.

**Fig. 9:**
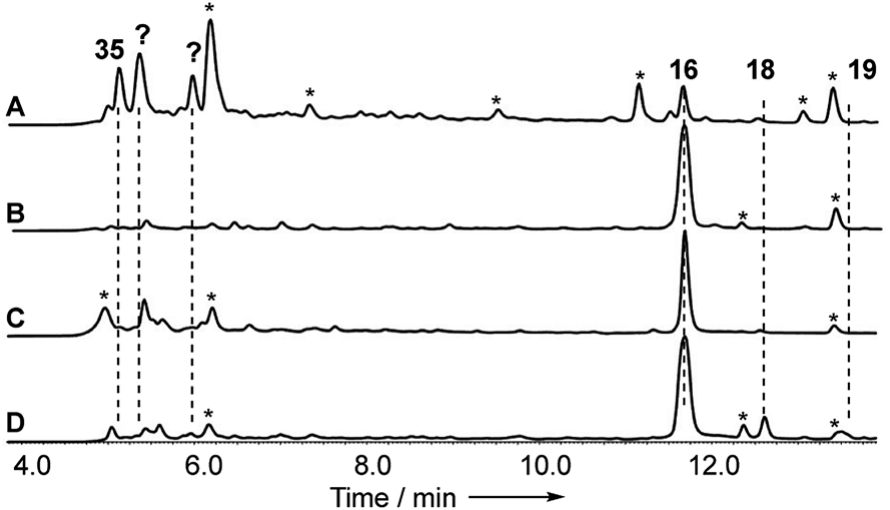
Analysis of knockout experiments: (A) LCMS analysis (DAD) of organic extracts from Δ*dmxR13* (*O*-acetyl transferase); (B) LCMS analysis (DAD) of organic extracts from Δ*dmxL1* (*acyl CoA carboxylase*); (C) LCMS analysis (DAD) of organic extracts from Δ*dmxL2* (hrPKS); (D) LCMS analysis (DAD) of organic extracts from WT (Cryptosporiopsis sp. 8999). All strains were grown on brown rice. LCMS method 10–90% CH_3_CN:H_2_O gradient, 15 min. * = unrelated metabolite.

**Scheme 1:**
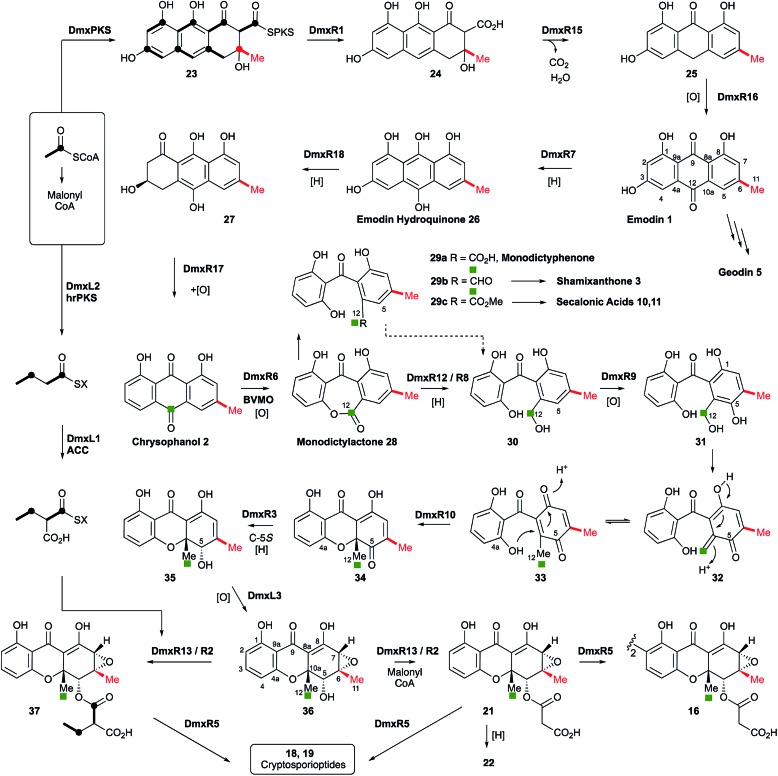
Proposed cryptosporioptide biosynthesis and relationships among monomeric and dimeric xanthone metabolites. Secalonic acid numbering used throughout.^20^ Red bond indicates polyketide starter unit. Green atom derived from C-10 of chrysophanol (=C-12 secalonic acid numbering).

Disruptions of *dmxL2* and *dmxL1* both abolished production of cryptosprioptide B **18** and C **19**, to give exclusively cryptosporioptide A **16** (Fig. 9B and C). Knockout of *dmxR13* gave a similar result, abolishing cryptosporioptide C **18** and D **19** production but retaining cryptosporioptide A **16** in smaller quantities than previously (Fig. 9D). A number of new metabolites were produced in small quantities that precluded structure elucidation although mass spectrometry indicated molecular weights consistent with monomeric xanthones, in particular non-malonylated monomeric xanthone **35** (Scheme 1).

## Discussion

From these results, a biosynthetic sequence for cryptosporioptides can be proposed (Scheme 1) and more general conclusions for the formation of dimeric xanthones made. The BGCs encoding the biosynthesis of shamixanthone **3** (*mdp*),^5^,^6^ geodin **5** (*ged*),^7^ agnestin **6** (*agn*)^8^ and cladofulvin **7**^9^ (*cla*) are all known. Tudzynski and coworkers recently revealed a putative secalonic acid (*sec*) BGC in *Claviceps purpurea*.^27^ In their analysis fifteen genes were described, but here, by comparison to the other known BGCs we extend this analysis to include six more genes in the *sec* cluster and two additional gene clusters from *Penicillium oxalicum* and *Aspergillus aculeatus* (Table 1 and see ESI†).

All analysed clusters encode proteins with high homologies (41–76% orf for orf) to proteins known to be involved in the biosynthesis of emodin hydroquinone **26**, and all BGCs except that for geodin contain genes which advance the pathway to chrysophanol **2** (Table 1). Thus, the nrPKS (DmxPKS) produces an enzyme-bound octaketide **23** which is released (DmxR1, giving **24**), decarboxylated (DmxR15, giving **25**) and oxidised (DmxR16) to give emodin **1**, followed by reduction (DmxR7) to emodin hydroquinone **26**. These steps are fully consistent with previous experimental results.^8^ A-ring reduction (DmxR18 giving **27**), dehydration (DmxR17) and probable spontaneous re-oxidation, results in overall deoxygenation to chrysophanol **2**, again consistent with previous results.^8^ Baeyer–Villiger oxidation (DmxR6) would then be expected to give monodictylactone **28** in equilibrium with monodictyphenone **29a** (R = CO_2_H) as we recently observed in the agnestin pathway.^8^ All pathways encode this BVMO except the *cla* cluster which does not form xanthones. We have previously^8^ demonstrated the existence of BVMO enzymes which have complementary regioselectivity, and such a BVMO is likely to be operating in the ascherxanthone **8** pathway in which C-12 (C-10 AQ numbering) becomes attached to the non-starter unit ring (Fig. 1).

At this stage we propose a branch-point in the pathway. Conversion of monodictyphenone **29a** (R = CO_2_H) to a methyl ester **29c** would direct the intermediates towards the secalonic acids which feature a distinctive C-12 methyl ester. Recent results from Matsuda and coworkers support this conclusion by showing that a specific methyltransferase NsrG directs monodictyphenone **29a** towards the biosynthesis of the heterodimeric xanthone neosartorin.^37^ This is consistent with the presence of methyltransferase-encoding genes (*CPUR_05424*, *P_oxa-09242* and *aacuQ*) in the secalonic acid BGCs but not the others (Table 1). Alternatively, reduction at C-12 to form an aldehyde **29b** would direct the pathway towards the shamixanthone group. In the case of the cryptosporioptides, however, reduction of C-12 to an alcohol **30** and hydroxylation at C-5 (likely DmxR9, see below) could give the electron-rich aromatic **31** which could eliminate H_2_O to form the *ortho*-quinonemethide **32**, followed by tautomerisation to *para*-quinone **33** and complete the formal reduction to produce the 10-methyl group.

C-5 hydroxylation is required in the cryptosporioptide **16**, shamixanthone **3** and secalonic acid **10**, **11** pathways. In shamixanthone **3**, this is proposed to be carried out by the monooxygenase MdpD, which shows 68% homology to DmxR9, while CPUR_05423 encodes a homolog in the *sec* cluster. Homologs of DmxR9 are also encoded in the *P. oxalicum* and *A. aculeatus* clusters. Notably homologs of *dmxR9* are missing from the *ged*, *agn* and *cla* clusters where this chemistry is not required.

In an early application of doubly ^13^C-labelled acetate feeding experiments, Vining and coworkers showed^38^ that during secalonic acid biosynthesis in *Pyrenochaeta terrestris*, the initial product of anthraquinone ring cleavage must have a symmetrical 1,3-dihydroxyphenyl ring as in **29–33**. Our suggested pathway is in agreement with this observation.

We propose that conjugate addition of C-4a-OH to the resulting *para*-quinone **33** then gives cyclohexadienone **34**, which is then reduced at C-5 to give the dihydroxanthone **35**. The ring closing reaction may be performed by DmxR10. This protein has no close BLAST hits with proteins of known function, but structural analysis using Phyre-2 (ref. 39) (see ESI†) shows that it contains a SnoAL domain^40^ and is related to proteins such as PhzB^41^ and Trt14 (ref. 42) known to be involved in secondary metabolite ring-forming reactions.

**Fig. 10:**
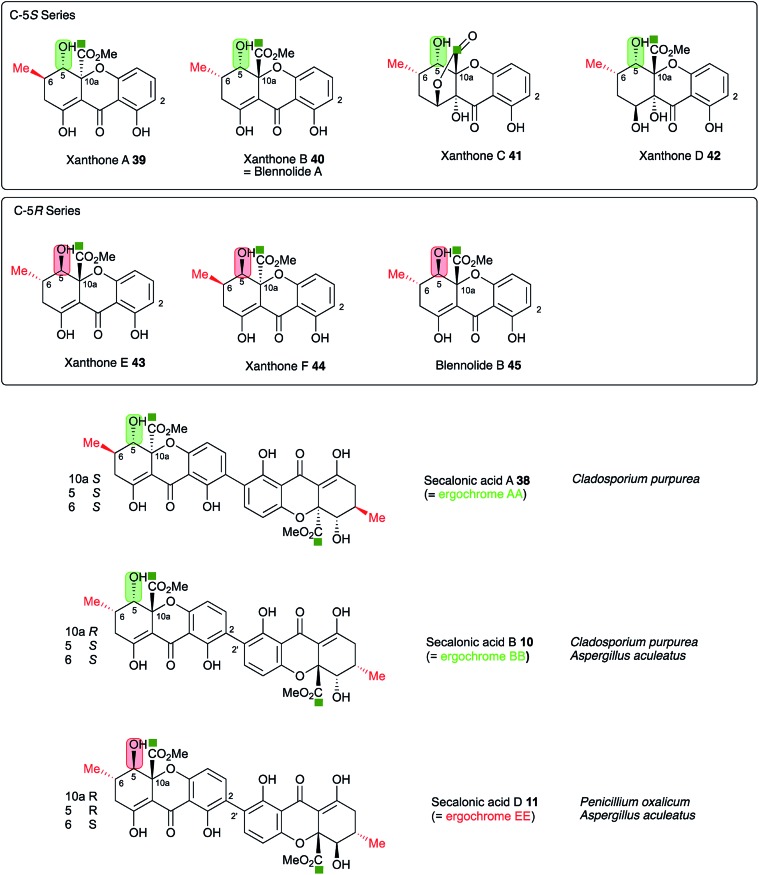
Stereochemical analysis of the known secalonic acids and correlation with producing organisms *Penicillium oxalicum*, *Aspergillus aculeatus* and *Claviceps purpurea*. Atom labelling according to Fig. 1.

In this step the stereochemistry at C-10a is set, and it is known that both epimers at this position can be formed in the cases of the secalonic acids (*e.g.* see Fig. 10). It is interesting to note that the three secalonic acid clusters examined here (Table 1) appear to encode multiple copies of this protein, conceivably explaining the presence of both 10a-epimers in these systems. However further experimental work will be required to confirm this hypothesis.

The 6,7-epoxide in the cryptosporioptides could be introduced by the cytochrome P450 monooxygenase encoded by *dmxL3*, which is unique to the *dmx* cluster, to give **36**. Our results suggest that the hrPKS (DmxL2) manufactures butyrate. This is consistent with domain analysis of DmxL2 (NCBI CDD)^43^ which shows it to consist of N-terminal KS and AT domains, followed by a DH domain. Highly-reducing PKS often feature a *C*-methyltransferase domain but this appears to be absent or inactive in DmxL2, consistent with the lack of methylation observed. Canonical C-terminal ER, KR and ACP domains make up the rest of the hrPKS. Butyrate is then carboxylated (DmxL1) to form ethylmalonate. It is not yet clear whether the carboxylation occurs while the butyrate is attached to the ACP of DmxL2, but this unusual fungal metabolite could then be esterified to *O*-5 by DmxR13.

In the absence of ethylmalonate or the acyl transferase DmxR13, malonyl CoA can be used, possibly transferred by DmxR2. Finally, dimerisation (DmxR5) gives the observed dimers **16**, **18** and **19** as the final products of the pathway. A homolog of *dmxR5* is found in the *C. purpurea sec* cluster (*CPUR_05419*, again previously unrecognised) and *P. oxalicum* and *A. aculeatus* clusters, and the cladofulvin cluster where *claM* has been already shown to direct oxidative dimerisation.^9^ However, *dmxR5* is missing from the *agn*, *mdp* and *ged* clusters as expected. The lower relative homology of the cladofulvin dimerase may reflect the fact that cladofulvin is a dimeric anthraquinone rather than a dimeric xanthone, and that cladofulvin is an asymmetric dimer in contrast to the symmetric cryptosporioptide and secalonic acid dimers.

A key step in the pathways to the dimeric xanthone metabolites is reduction at C-5. This could result in either 5*S* or 5*R* products depending on the selectivity of the reductase. Both types of xanthones are known^44^ (Fig. 10) and are, as shown above, likely to be direct precursors of the secalonic acids. Indeed, the ergochrome nomenclature explicitly recognises this, such that secalonic acid A **38** is also known as ergochrome AA showing that it is a dimer of two xanthone A **39** units. Xanthones A **39**, B **40**, C **41** and D **42** have C-5*S* stereochemistry, while xanthones E **43** and F **44** and blennolide B **45** have 5*R* stereochemistry. *C. purpurea* produces secalonic acids which are derived from xanthones A–D and it thus must reduce to give exclusively 5*S* stereochemistry. In contrast *P. oxalicum* produces secalonic acid D **11** which derives from xanthone E **43** which has 5*R* stereochemistry. *Aspergillus aculeatus*, however, produces both secalonic acids B **10***and* D **11** which are made from xanthones B **40***and* E **43** and it must therefore be able to reduce at C-5 to give *both* possible stereoisomers. In accord with this observation the *sec* cluster encodes a single SDR, which is homologous to DmxR3 (58%). The cryptosporioptides **16**, **18** and **19** and secalonic acids A **38** and B **10** possess 5*S* stereochemistry, so we propose that DmxR3 is a 5*S* reductase. The *P. oxalicum* cluster possesses a different SDR encoded by P_oxa-09231 and we propose that this is a 5*R* selective reductase. In agreement with these ideas the *A. aculeatus* cluster encodes *two* SDRs, one of which (AacuD) is homologous to the 5*S* reductase DmxR3, while the other (AacuF) is homologous to the 5*R* selective P_oxc-09231. Homologous SDRs are not encoded by the *agn*, *geo* or *cla* clusters where the pathways do not require C-5 reduction, again consistent with this hypothesis.

## Conclusions

Here we have shown that the originally reported monomeric structures of the cryptosporioptides must be reassigned to a series of dimeric xanthones featuring unusual malonate substituents and full reduction at C-10 (=C-12 in SA nomenclature). Isolation of the cryptosporioptide BGC and its verification by directed knockout experiments allowed the unusual biosynthesis and attachment of the ethylmalonyl substituents to be determined. Furthermore, comparison with known, but partially characterised fungal gene clusters, has allowed a fuller hypothesis regarding the biosynthesis of the dimeric xanthones in general, and the secalonic acids in particular, to be developed. Questions remain surrounding the proposed reduction of monodictylactone **28** in the cryptosporioptide and shamixanthone pathways where evidence for enzyme candidates is lacking. Similarly the catalyst for xanthone formation in the shamixanthone pathway is unknown, although the cryptosporioptide, agnestin and secalonic acid pathways appear to use homologs of DmxR10 for this step. Missing catalysts in the shamixanthone pathway may be related to the fact that the shamixanthone BGC is split, with outlying prenyltransferase genes and at least one redox-encoding gene located in a BGC distant from the core PKS-encoding BGC so the ‘missing’ genes may be elsewhere on the *A. nidulans* genome. However, the remaining very close genetic, and therefore chemical, homologies of the pathways revealed here should allow further targeted engineering in known dimeric xanthone clusters to be designed and performed, and it should also allow clusters encoding new dimeric xanthones to be more rapidly recognised and discovered.

## Conflicts of interest

There are no conflicts to declare.

## Supplementary Material

Supplementary information
