# Supplementary material for: Structure revision of cryptosporioptides and determination of the genetic basis for dimeric xanthone biosynthesis in fungi
Source: Chem Sci. 2019 Jan 21;10(10):2930–9. doi: 10.1039/c8sc05126g (PMC6428139; doi:10.1039/c8sc05126g)
Supplement: Supplementary file 1 [file SC-010-C8SC05126G-s001.pdf]

Electronic Supplementary Material (ESI) for Chemical Science.
This journal is © The Royal Society of Chemistry 2019

# Supplementary information – Structure Revision of Cryptosporioptides and Determination of the Genetic Basis for Dimeric Xanthone Biosynthesis in Fungi

## Contents


1. General procedure ..... 2
2. NMR ..... 2
3. HRMS ..... 2
4. LCMS ..... 2
5. Strains ..... 3
6. Growth conditions and method of extraction ..... 3
7. Fungal nucleic acid preparation ..... 4
8. Analysis cluster ..... 4
9. Primer table ..... 7
10. Transformation ..... 9
    - *E. coli* transformation ..... 9
    - *S. cerevisiae* transformation ..... 9
    - *Cryptosporiopsis* sp. transformation ..... 9
11. Genetic characterisation of the transformants ..... 10
    - Genetic characterisation of the nrPKS disruption (*dmxPKS*) ..... 10
    - Genetic characterisation of the P450 dimerase disruption (*dmxR5*) ..... 11
    - Genetic characterisation of the BVMO dimerase disruption (*dmxR6*) ..... 11
    - Genetic characterisation of the *O*-acyl transferase (*dmxR13*), the acyl CoA carboxylase (*dmxL1*) and hrPKS (*dmxL2*) disruptions ..... 12
    - 12. Mass spectra and UV spectra ..... 13

FAB MS of cryptosporioptide 17 ..... 15

13. Structure elucidation and derivatisation ..... 15
14. NMR data ..... 19

References ..... 38

## 1. General procedures

Analytical grade chemicals and reagents were supplied from Sigma-Aldrich, Alfa Aesar, Acros Organics, Becton-Dickinson, BDH, Fischer, Fluka and Difco, unless otherwise stated. Solvents used for LC-DAD-MS analyses were HPLC grade. General molecular biology procedures were performed as standard and molecular biology kits used according to manufacturer's protocols. Analytical PCR was performed using BioMix Red (Bioline), Kapa HiFi (Kapabiosystems) and KOD (Merck Millipore) polymerases. Optical rotations were recorded at the sodium D line (λ = 589 nm) on a Bellingham and Stanley ADP220 polarimeter. Infrared spectra were recorded on a Perkin Elmer Spectrum One FT-IR spectrometer in the solid or liquid state. Sterilisation of all media, glassware and items used in microbiological work was carried out using Astell autoclave at 121 °C for 15 minutes. Glycerol solutions were filtered using 0.22 µm syringe filters.

## 2. NMR

NMR experiments were conducted on the following spectrometers: Varian 400-MR Varian ($^{1}$H NMR at 400 MHz and $^{13}$C NMR at 100 MHz), Varian VNMR S500 spectrometer, ($^{1}$H NMR at 500 MHz, $^{19}$F-NMR at 470 MHz and $^{13}$C NMR at 125 MHz), Bruker Avance III HD Cryo $^{13}$C-probe, ($^{1}$H NMR at 500 MHz and $^{13}$C NMR at 125 MHz) and Bruker 700 micro-cryo ($^{1}$H NMR at 700 MHz). Chemical shifts were recorded in parts per million (ppm referenced to the appropriate residual solvent peak) and coupling constant (*J*) in Hz, reported to the closest 0.5 Hz. Multiplicity is described by the following abbreviations: s, singlet; d, doublet; t, triplet; q, quartet; m, multiplets.

## 3. HRMS

The mass analyser detectors used were electrospray ionization (ESI) mass spectra, recorded on a MicrO-ToF II Bruker Daltonics mass spectrometer and Orbitrap Elite Thermo Scientific mass spectrometer, and fast atom bombardment ionisation (FAB) mass spectrum recorded on a ThermoElectron MAT 900 (University of Edinburgh).

## 4. LCMS

### 4.1 Analytical LCMS

All crude extracts were prepared to a concentration of 10 mg/ml in HPLC grade acetonitrile and placed in LCMS vials. 20 µl of the extracts were injected and analysed using one of two Waters LCMS instruments.

(1) Waters 2795HT; Phenomenex Kinetex column (2.6 µ, $C_{18}$, 100 Å, 4.6 ×100 mm, flow rate 1 mL/min) equipped with a Phenomenex Security Guard precolumn (Luna $C_5$ 300 Å). Detection was achieved using Waters 998 diode array detector for UV between 200 and 400 nm; Waters Micromass ZQ ESI mass spectrometry in $ES^+$ and $ES^-$ modes between 100 *m/z* and 1000 *m/z*; Solvents were: **A**, HPLC grade $H_2O$ containing 0.05% formic acid; **B**, HPLC grade MeOH containing 0.045% formic acid; and **C**, HPLC grade $CH_3CN$ containing 0.045% formic acid. Gradients were: *Method 1* - Kinetex/$CH_3CN$: 0 min, 5% **C**; 10 min, 90% **C**; 12 min, 90% **C**; 13 min, 5% **C**; 15 min, 5% C. *Method 2* - Kinetex/$CH_3CN$: 0 min, 5% **C**; 2 min, 50% **C**; 12 min, 95% **C**; 13 min, 5% **C**; 15 min, 5% **C**.

(2) Waters 2445SFO HPLC; Waters 2767 autosampler, Phenomenex Kinetex column (2.6 µ, $C_{18}$, 100 Å, 4.6 × 100 mm) equipped with a Phenomenex Security Guard precolumn (Luna $C_5$ 300 Å) eluted at 1 mL/min. Detection was achieved by Waters 2298 diode array detector between 200 and 400 nm; Waters Quattro Micro ESI mass spectrometer in $ES^+$ and $ES^-$ modes between 100 *m/z* and 1000 *m/z*; Waters 2424 ELS detector. Solvents were: A, HPLC grade $H_2O$ containing 0.05% formic acid; B, HPLC grade MeOH containing 0.045% formic acid; and C, HPLC grade $CH_3CN$ containing 0.045% formic acid. Gradients were: *Method 3* - Kinetex/$CH_3CN$: 0 min, 5% C; 15 min, 90% C; 12 min, 90% C; 17 min,

95% C; 18 min, 5% C, 20 min, 5% C. *Method 4* - Kinetex/$CH_3CN$: 0 min, 5% C; 2 min, 50% C; 15 min, 95% C; 17 min, 95% C; 18 min, 5% C, 20 min, 5% C. *Method 5* - Kinetex/$CH_3CN$: 0 min, 5% C; 2 min, 5% C; 25 min, 90% C; 27 min, 95% C; 28 min, 5% C, 30 min, 5% C. *Method 6* - Kinetex/$CH_3CN$: 0 min, 5% C; 2 min, 50% C; 26 min, 95% C; 27 min, 95% C; 28 min, 5% C, 30 min, 5% C.

### 4.2 Preparative LCMS

Compounds were generally purified using a Waters mass or time directed autopurification system compromising Waters 2767 autosampler, Waters 2545 pump system, Phenomenex Kinetex column (5 µ, $C_{18}$, 100 Å, 250 × 21.20 mm) equipped with Phenomenex Security Guard precolumn (Luna $C_5$ 300 Å) eluted at 16 mL/min. Solvents were: **A**, HPLC grade $H_2O$ containing 0.05% formic acid; **B**, HPLC grade MeOH containing 0.045% formic acid; and **C**, HPLC grade $CH_3CN$ containing 0.045% formic acid. The post column was split (100:1) with the minority flow made up with HPLC grade MeOH containing formic acid 0.045% to 1 mL/min for simultaneous analysis by Waters 2298 diode array detector between 200 and 400 nm; Waters Quattro Micro ESI mass spectrometer in $ES^+$ and $ES^-$ modes between 100 *m/z* and 1000 *m/z*; Waters 2424 ELS detector. Metabolites were collected into glass test tubes. Combined samples were evaporated under $N_2$ gas, weighed and dissolved in NMR solvent for NMR analysis.

## 5. Strains

The fungal strains used in this project were *Cryptosporiopsis* sp. 8999 and *Saccharomyces cerevisiae* (Stratagene) strain BY4742 (*MATα*, *his3Δ-1*, *leu2Δ-0*, *lys2Δ-0*, *ura3Δ-0*). *Escherichia coli* competent strain was used for plasmid manipulations TOP10 (Invitrogen). *Bacillus subtilis* strain ATCC 6633 was used for all plate-based bioassays for antibiotic activity.

## 6. Growth conditions and method of extraction

*Cryptosporiopsis* sp. was grown on rice (50 g) prepared by adding 50 mL of deionised water and autoclaved. After 28 days at 25°C the rice was blended and soaked in ethyl acetate (100 mL). After 2 hours the solid was remove by vacuum filtration. The organic layer was washed with water (2 × 50 mL), dried over anhydrous magnesium sulfate and concentrated under reduced pressure. The crude was dissolved in acetonitrile (50 mL) and defatted using hexane (2 × 25 mL). The acetonitrile layers were combined and concentrated under a stream of $N_2$ gas.

*Cryptosporiopsis* sp. was grown in 100 mL GNB (20 g glucose and 30 g Nutrient Broth No. 2 LabM in 1.0 L of water) in a 500 mL conical flask at 25 °C. After 1, 2 and 3 weeks the broth was separated from the mycelia using vacuum filtration. The mycelia were soaked in water (50 mL), acidified to pH 3 using HCl and blended in ethyl acetate (100 mL). After 1 hour, the mycelia were removed by vacuum filtration and the organic solution was washed with water (3 × 25 mL). The combined organic phases were dried over anhydrous magnesium sulfate and concentrated under reduced pressure.

*Cryptosporiopsis* sp. was also grown on 25 mL of MEA in a 90 mm petri dish wrapped with Parafilm at 25 °C. After 28 days, the whole agar plate was blended, soaked in ethyl acetate (100 mL) and water (50 mL, acidified to pH 3.5 using HCl). After 2 hours, the solid was removed using vacuum filtration and the organic layer was separated. The aqueous layer was extracted with ethyl acetate (2 × 25 mL). The combined organic phases were dried over anhydrous magnesium sulfate and concentrated under reduced pressure.

## 7. Fungal nucleic acid preparation

The wild type and transformants *Cryptosporiopsis* strains were grown in GN media (10 mL) in a 50 mL tube at 25 °C for 3 days. Then they were centrifuged (6000 g), the pellet was lyophilized and ground under liquid nitrogen. Genomic DNA for both sequencing and PCR analysis was prepared using the GenElute Plant Genomic DNA Miniprep kit (Sigma).

## 8. Cluster Analysis

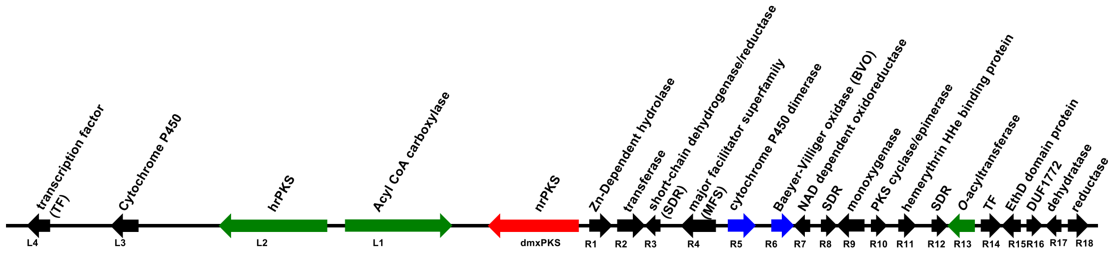


**Figure S1.** The putative BGC (*dmx*) of the cryptosporioptides **16**, **18** and **19.**

**Table S1A.** Summary of the predicted functions for the genes in the putative cryptosporioptide cluster. The function was determined using NCBI BLAST in the Swissprot database. *Indicates where the putative function was determined using non-redundant protein sequences. The E-value indicates the likelihood of such an alignment occurring by chance. The score indicates the overall quality of alignment.

| Protein | Putative function with cofactors | Homologue | Ref. | Gene identity | E-value | Query coverage | Score |
|---|---|---|---|---|---|---|---|
| DmxL4 | Transcription factor | V439_05325* | - | 91% | $2e^{-75}$ | 91% | 254 |
| DmxL3 | Heme-thiolate Cytochrome P450 | MepH | 2 | 34% | $9e^{-96}$ | 93% | 301 |
| DmxL2 | HR-PKS (SAT, KS, AT, DH, ER KR, ACP) | Hmp8 | 3 | 40% | 0.0 | 99% | 1513 |
| DmxL1 | Acyl CoA carboxylase | ACC | 4 | 57% | $3e^{-8}$ | 98% | 2600 |
| DmxPKS | NR-PKS (KS, AT, PT, ACP) | MdpG | 5 | 66% | 0.0 | 99% | 2399 |
| DmxR1 | Zinc-dependent hydrolase / thioesterase | TpcB | - | 66% | $3e^{-157}$ | 96% | 362 |
| DmxR2 | Transferase / acyl transferase | LysR | 6 | 42% | $1e^{-118}$ | 94% | 274 |
| DmxR3 | Short-chain dehydrogenase / NADPH-reductase (SDR) | XP_002144863.1* | - | 74% | $3e^{-118}$ | 80% | 359 |
| DmxR4 | Major facilitator superfamily | AflT | 7 | 44% | $3e^{-12}$ | 78% | 359 |
| DmxR5 | Heme-thiolate cytochrome P450 | KFX52354 | 8 | 56% | 0.0 | 98% | 606 |
| DmxR6 | Baeyer-Villiger Oxidase | MdpL | 9 | 47% | $2e^{-128}$ | 93% | 379 |
| DmxR7 | NADPH-dependent oxidoreductase | MdpK | 9 | 62% | $6e^{-101}$ | 99% | 321 |
| DmxR8 | SDR (NADPH) | TropG | 10 | 31% | $1e^{-36}$ | 94% | 134 |
| DmxR9 | FAD-monooxygenase | MdpD | 9 | 69% | 0.0 | 98% | 729 |
| DmxR10 | PKS cyclase SnoaL / epimerase | XP_002144875.1* | - | 95% | $1e^{-79}$ | 88% | 238 |
| DmxR11 | hemerythrin HHE binding protein | XP_002144874.1* | - | 82% | $3e^{-162}$ | 82% | 456 |
| DmxR12 | SDR (NADPH) | XP_002144873.1* | - | 80% | $5e^{-125}$ | 98% | 360 |
| DmxR13 | *O*-acyl transfrease | AzaD | 11 | 33% | $2e^{-68}$ | 38% | 225 |
| DmxR14 | TF | TpcE | 12 | 27% | $2e^{-30}$ | 94% | 121 |
| DmxR15 | EtH domain protein decarboxylase Anthrone decarboxylase | TpcK MdpH1 | 12, 13 | 72% 59% | $3e^{-71}$ $2e^{-58}$ | 88% 89% | 210 186 |

| | | | | | | | |
|---|---|---|---|---|---|---|---|
| DmxR16 | DUF1772 anthrone oxidase/ | GeoH | 14 | 42% | $1e^{-37}$ | 78% | 126 |
| DmxR17 | Dehydratase | MdpB | 9 | 68% | $8e^{-74}$ | 92% | 220 |
| DmxR18 | NADPH-Reductase | MdpC | 9 | 80% | $1e^{-161}$ | 99% | 449 |

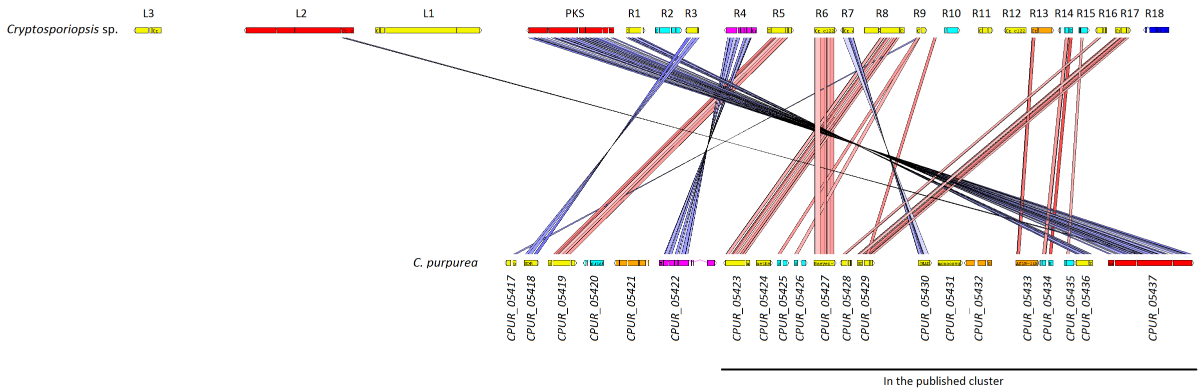


**Figure S2A.** Comparison of the putative cryptosporioptide BGC and the secalonic acid BGC using Artemis Comparison Tool. Lines show sequence homology.

**Table S1B**. Comparison of the cryptosporioptide cluster and the secalonic acid cluster from *Claviceps purpurea* (Neubauer et al., 2016)

| *C. PURPUREA* | CRYPTO | % IDENTITY | PREDICTED FUNCTION |
|---|---|---|---|
| ***CPUR_05417** | DmxR10 | 20.0 | SnoaL-like |
| ***CPUR_05418** | DmxR3 | 58.0 | SDR |
| ***CPUR_05419** | DmxR5 | 39.7 | Cytochrome P450 |
| ***CPUR_05420** | – | – | Hypothetical |
| ***CPUR_05421** | – | – | Transcription factor |
| ***CPUR_05422** | DmxR4 | 47.9 | MFS transporter |
| ***CPUR_05423*** | DmxR9 | 55.8 | Monooxygenase |
| ***CPUR_05424*** | – | – | Methyltransferase |
| ***CPUR_05425*** | DmxR10 | 21.3 | Hypothetical |
| ***CPUR_05426*** | DmxR10 | 21.8 | Hypothetical |
| ***CPUR_05427*** | DmxR6 | 53.7 | Baeyer-villiger oxidase |
| ***CPUR_05428*** | DmxR17 | 69.6 | Dehydratase |
| ***CPUR_05429*** | DmxR18 | 85.6 | Reductase |
| ***CPUR_05430*** | DmxR7 | 51.8 | Oxidoreductase |
| ***CPUR_05431*** | – | – | Monooxygenase |
| ***CPUR_05432*** | – | – | Coactivator |
| ***CPUR_05433*** | DmxR14 | 32.8 | Transcription factor |
| ***CPUR_05434*** | DmxR15 | 76.0 | Decarboxylase |
| ***CPUR_05435*** | DmxR16 | 37.6 | Anthrone Oxidase |
| ***CPUR_05436*** | DmxR1 | 65.5 | Hydrolase |
| ***CPUR_05437*** | DmxPKS | 58.5 | Non-reducing PKS |
| – | DmxL5** | – | Hypothetical |
| – | DmxL4** | – | Transcription factor |
| – | DmxL3 | – | Cytochrome P450 |
| – | DmxL2 | – | hrPKS |
| – | DmxL1 | – | Acyl coA carboxylase |
| – | DmxR2 | – | Transferase |
| – | DmxL8 | – | SDR |
| – | DmxR11 | – | Hemerythrin-like domain protein |
| – | DmxR12 | – | SDR |
| – | DmxR13 | – | *O*-acetyltransferase |

* Genes missed from the published annotation of the *C. purpurea* secalonic acid cluster

** RNAseq data suggests that these genes are outside the cryptosporioptide gene cluster boundary

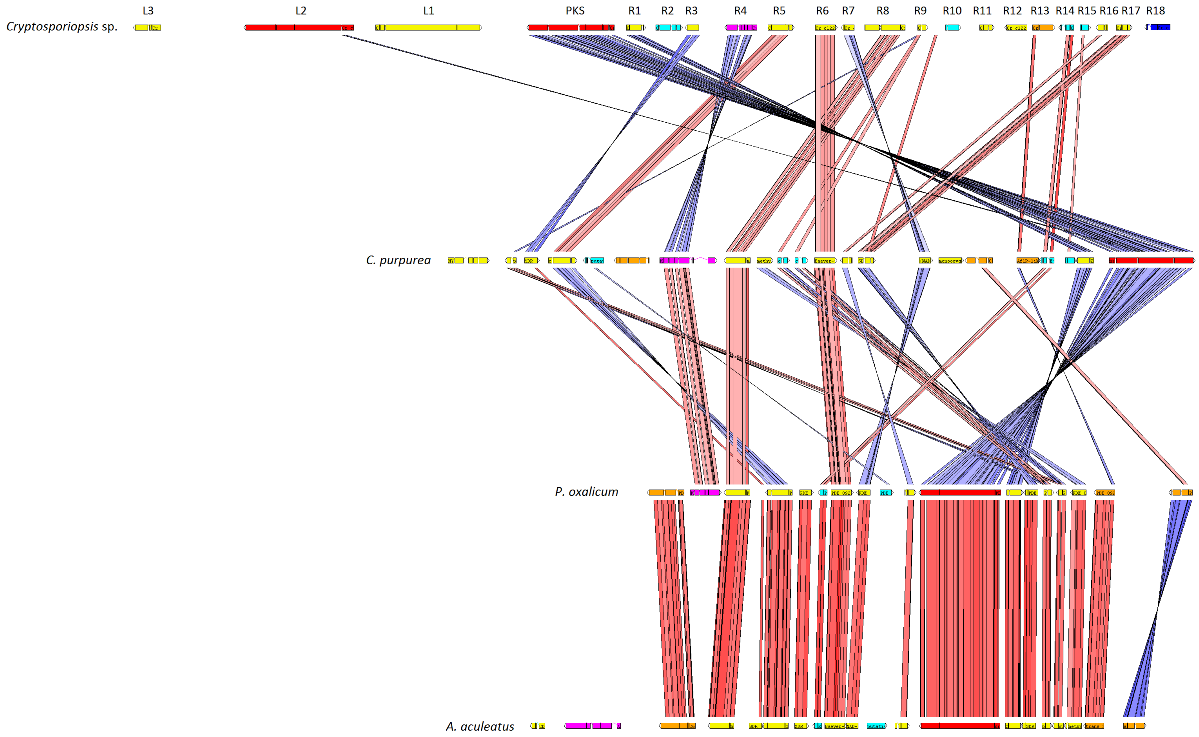


**Figure S2B**. The gene clusters can also be aligned with gene clusters from the other secalonic acid producers; *P. oxalicum* and *A. aculeatus*. Although these gene clusters have very different gene organisation they have almost the identical set of genes as *C. purpurea*:

## 9. Primer table

PCR was used to amplify DNA to test fungal and bacterial transformants, to amplify specific sequence (e.g. ITS) and to provide DNA fragments for recombination. The PCR machines used were SureCycler 8800 (Agilent Technologies) and MultiGene™ Mini (Labnet). Primers were synthesised by Sigma-Aldrich (Custom Oligos).

### Primers

**Table S2.** Primer sequences for KO plasmid construction of dmx BGC genes.

| Target | Primer | Sequence 5'-3' |
|---|---|---|
| ITS | ITS1 | TCCGTAGGTGAACCTGCGG |
| | ITS4 | TCCTCCGCTTATTGATATGC |

| Fragment | Forward Primer | Reverse Primer | Size (bp) |
|---|---|---|---|
| ***hygR* cassette** | | | |
| PCR3 | CATGATGGGGATCCTCTAGTG | CGTCAGGACATTGTTGGAG | 2795 |
| PCR4 | GCTTTCAGCTTCGATGTAGG | CAGGTCGAGTGGAGATGTG | 1789 |
| ***dmxPKS* (NR-PKS)** | | | |
| PCR1-LH | TAATGCCAACTTTGTACAAAAAAGCAGGCTATGAAGGTGGCATATTTCAG | TAATGCCAACTTTGTACAAAAAAGCAGGCTATGAAGGTGGCATATTTCAG | 2055 |
| PCR2-RH | AGCGCCCACTCCACATCTCCACTCGACCTGGTTAGGCAAAGTCATCTTCG | AATGCCAACTTTGTACAAGAAAGCTGGGTGCAAAGGACGCAGACAATTC | 2063 |
| ***dmxR6* (BVMO)** | | | |
| PCR1-LH | TAATGCCAACTTTGTACAAAAAAGCAGGCTTAACCTCCGGAATAATAGCC | CGAAAGATCCACTAGAGGATCCCCATCATGAAGGACGTATTCCTGGATAG | 918 |
| PCR2-RH | AGCGCCCACTCCACATCTCCACTCGACCTGCTGAGATGATTAGCACATGC | AATGCCAACTTTGTACAAGAAAGCTGGGTTTCCAGAGCCTATTGTAGAAC | 926 |
| ***dmxR5* (P450)** | | | |
| PCR1-LH | TAATGCCAACTTTGTACAAAAAAGCAGGCTGGCTTTGCACGACTTCCGAT | CGAAAGATCCACTAGAGGATCCCCATCATGTCTACGAGCACGAGTCATAC | 1079 |
| PCR2-RH | AGCGCCCACTCCACATCTCCACTCGACCTGATCCAGTGCGATTCCTATTC | AATGCCAACTTTGTACAAGAAAGCTGGGTTCATATCAGAGCGAGCCTAT | 1131 |
| ***dmxL2* (HR-PKS)** | | | |
| PCR1-LH | TAATGCCAACTTTGTACAAAAAAGCAGGCTGAGATCATATGCCGACCAC | CGAAAGATCCACTAGAGGATCCCCATCATGGAGTTCCTAGTGCTCATGGA | 994 |
| PCR2-RH | AGCGCCCACTCCACATCTCCACTCGACCTGGTACAGACCGATGTAGAGGC | AATGCCAACTTTGTACAAGAAAGCTGGGTCTAGCATCCAGTCCCAGTGA | 1219 |
| ***dmxL1* (Acyl CoA Carboxylase)** | | | |
| PCR1-LH | TAATGCCAACTTTGTACAAAAAAGCAGGCTGCTCACGTTAATGGCTACGT | CGAAAGATCCACTAGAGGATCCCCATCATGGCATACTGCACCACATATTG | 2055 |
| PCR2-RH | AGCGCCCACTCCACATCTCCACTCGACCTGCGATATCACCTTCCGAGTAG | AATGCCAACTTTGTACAAGAAAGCTGGGTCTTGGCGGATAACATACTG | 2039 |
| ***dmxR13* (*O*-acyl transferase)** | | | |
| PCR1-LH | TAATGCCAACTTTGTACAAAAAAGCAGGCTCGTCAATCCTGACGTTAGAC | CGAAAGATCCACTAGAGGATCCCCATCATGTGAGGAGTTCACGAAGTATG | 937 |
| PCR2-RH | AGCGCCCACTCCACATCTCCACTCGACCTGGAGTGAGGTAGGGCTGAAGA | AATGCCAACTTTGTACAAGAAAGCTGGGTACGGACTCCGAGCTCTATCT | 927 |

**Table S3.** Primer sequences for bipartite DNA fragment for KO of dmx BGC genes.

| Fragment | Forward Primer | Reverse Primer | Size (bp) |
|---|---|---|---|
| ***dmxPKS* (NR-PKS)** | | | |
| PCR1-LH | TAATGCCAACTTTGTACAAAAAAGCAGGCTATGA AGGTGGCATATTTCAG | CGTCAGGACATTGTTGGAG | 4790 |
| PCR2-RH | GCTTTCAGCTTCGATGTAGG | AATGCCAACTTTGTACAAGAAAGCTGGGTGCAAAG GACGCAGACAATTC | 3714 |
| ***dmxR6* (BVMO)** | | | |
| PCR1-LH | TAATGCCAACTTTGTACAAAAAAGCAGGCTTAAC CTCCGGAATAATAGCC | CGTCAGGACATTGTTGGAG | 3683 |
| PCR2-RH | GCTTTCAGCTTCGATGTAGG | AATGCCAACTTTGTACAAGAAAGCTGGGTTTCCAG AGCCTATTGTAGAAC | 2606 |
| ***dmxR3* (P450)** | | | |
| PCR1-LH | TAATGCCAACTTTGTACAAAAAAGCAGGCTGGCT TTGCACGACTTCCGAT | CGTCAGGACATTGTTGGAG | 3845 |
| PCR2-RH | GCTTTCAGCTTCGATGTAGG | AATGCCAACTTTGTACAAGAAAGCTGGGTTCATAT CAGAGCGAGCCTAT | 2811 |
| ***dmxL2* (HR-PKS)** | | | |
| PCR1-LH | TAATGCCAACTTTGTACAAAAAAGCAGGCTGAG ATCATATGCCGACCAC | CGTCAGGACATTGTTGGAG | 3759 |
| PCR2-RH | GCTTTCAGCTTCGATGTAGG | GACGATAGATCTCCTGCACT | 2807 |
| ***dmxL1* (Acyl CoA Carboxylase)** | | | |
| PCR1-LH | TAATGCCAACTTTGTACAAAAAAGCAGGCTGCTC ACGTTAATGGCTAC GT | CGTCAGGACATTGTTGGAG | 4820 |
| PCR2-RH | GCTTTCAGCTTCGATGTAGG | AATGCCAACTTTGTACAAGAAAGCTGGGTCTTGGC GGATAACATACTG | 3719 |
| ***dmxR13* (*O*-acyl transferase)** | | | |
| PCR1-LH | TAATGCCAACTTTGTACAAAAAAGCAGGCTCGTC AATCCTGACGTTAGAC | CGTCAGGACATTGTTGGAG | 3702 |
| PCR2-RH | GCTTTCAGCTTCGATGTAGG | AATGCCAACTTTGTACAAGAAAGCTGGGTACGGAC TCCGAGCTCTATCT | 2607 |

**Table S4.** Primer sequences for testing transformants of the *dmx* BGC KO.

| Fragment | Forward Primer | Reverse Primer | Size (bp) |
|---|---|---|---|
| ***dmxPKS* (NR-PKS)** | | | |
| PCR1-LH | CCTTTGTGGAGCCTGGCAA | GCTCGACGTATTTCAGTGTC | 2063 |
| PCR2-RH | GCTCCGTAACACCCAATACG | AACTGCCATTAACCGCACGG | 2264 |
| PCR3-purity | CAGGAAGTCTGGCTCCATTA | CATCAGATGCTTGCTGTACG | 869 |
| ***dmxR6* (BVMO)** | | | |
| PCR1-LH | GTAACGCTACCGGCAGTATT | CGTCAGGACATTGTTGGAG | 3806 |
| PCR2-RH | GCTTTCAGCTTCGATGTAGG | GCTCACGTGAATTGTTAGAC | 2673 |
| PCR3-purity | GTAACGCTACCGGCAGTATT | AGTGAATGATGGGGTGGTAT | 1061 |
| ***dmxR5* (P450)** | | | |
| PCR1-LH | CCTCCAATTCAAGGAGATGG | CGTCAGGACATTGTTGGAG | 4245 |
| PCR2-RH | GCTTTCAGCTTCGATGTAGG | ACCGCTACAGTACATCTTGG | 2790 |
| PCR3-purity | CCTCCAATTCAAGGAGATGG | ACCGCTACAGTACATCTTGG | 2557 |
| ***dmxL2* (HR-PKS)** | | | |
| PCR1-LH | AGATCATATGCCGACCAC | GCTCGACGTATTTCAGTGTC | 997 |
| PCR2-RH | GCTCCGTAACACCCAATACG | TAGGATCAAGAACCCGAG | 1315 |
| PCR3-purity | AGATCATATGCCGACCAC | AAGACATGCGACGACTGT | 2047 |
| ***dmxL1* (Acyl CoA Carboxylase)** | | | |
| PCR1-LH | CCTGCTTGTAACAACAGCCT | CGTCAGGACATTGTTGGAG | 4894 |
| PCR2-RH | AGGGCGAAGAATCTCGTGCT | CATCATTACAACACGCACAC | 3852 |
| PCR3-purity | CCTGCTTGTAACAACAGCCT | CAATAGATCGTATGGCTCTG | 2364 |
| ***dmxR13* (*O*-acyl transferase)** | | | |
| PCR1-LH | CTTGTGCTTGATATCCTGGT | GCTCGACGTATTTCAGTGTC | 1104 |
| PCR2-RH | GCTCCGTAACACCCAATACG | GCTCGACGTATTTCAGTGTC | 1011 |
| -PCR3-purity | CTTGTGCTTGATATCCTGGT | GTAACCAGCCAATGAATGTC | 2534 |

## 10. Transformation

### *E. coli* transformation

Competent *E. coli* cells were removed from -80 °C and thawed on ice. For the transformation 50 µL were transferred to a chlled micro-centrifuge tube, 5 µL of DNA was added and they were incubated on ice for 30 minutes. The cells were heat shocked at 42 °C for 30 seconds and returned to ice. 125 µL of SOC medium was added and the cells were incubated at 37 °C for 1 hour. The mix was plated on LB plates containing the appropriate antibiotic for selection (carbenicillin at 100 µg/mL and kanamycin at 50 µg/mL). The plates were the incubated overnight at 37 °C.[15]

### *S. cerevisiae* transformation

A single colony of *S. cerevisiae* was inoculated into a 10 mL YPDA starter culture and incubated at 28 °C shaking at 200 rpm overnight. The starter culture was then added into a 250 mL flask containing 40 mL of YPAD and incubated for 5 hours at 28 °C shaking at 200 rpm. After 5 hours the culture was separated into two 50 mL tubes and centrifuged at 3000 x g for 5 minutes. The supernatant was discarded and the cells were washed with 10 mL of sterile water per tube. The water was removed by centrifugation (3000 x g for 5 minutes) and 500 µL of 0.1 M LiOAc was transferred in two 1.5 mL microfuge tubes. The cells were centrifuged at 14500 rpm for 15 seconds, the supernatant discarded and suspended in 200 µL of 0.1 M LiOAc by vortexing. For each transformation 50 µL were added to a fresh 1.5 mL microfuge tube and pelleted by spinning at 14500 rpm for 15 seconds. After discarding the supernatant 240 µL of PEG solution (50 % w/v PEG 3350), 36 µL of 1 M LiOAc, 50 µL of salmon sperm DNA (2 mg of denatured salmon testis DNA per mL of TE buffer) and 34 µL of DNA were added to the cells in order. Approximately 0.5-1 µL of each DNA fragment was added with linear DNA fragments to be joined containing at least 30 bp overlap. The cells were resuspended in the transformation by vortexing and incubated at 30 °C for 30 minutes and then at 42 °C for another 30 minutes. The cells were pelleted at 6000 rpm for 15 seconds and then gently resuspended in 1 mL of sterile water. 200 µL aliquots were spread on SM-URA plates and incubated ay 28 °C for 3-4 days.[16]

### *Cryptosporiopsis* sp. transformation

The protocol was developed by optimisation of an *Aspergillus oryzae* transformation protocol.[17] Three small plugs of conidia grown on MEA at 25 °C for one week at from plates were used to inoculate 50 mL GN medium and incubated at 25 °C shaking at 200 rpm. After 3 days the culture broth was homogenised and 10 mL were used to inoculate fresh GN flasks (100 mL in 500 mL conical flask) that were cultivated at 25 °C shaking at 200 rpm for another 3 days. The young hyphae were centrifuged at 8000 rpm for 10 minutes and the supernatant discarded. The hyphae were washed with sterilised water and 0.8 M sodium chloride solution. The mycelia were resuspended in 10 mL of filter sterilised protoplasting solution (20 mg/mL *Trichoderma* lysing enzyme and 5 mg/mL driselase in 0.8 M NaCl) and gently mixed for 3 hours. The protoplasts were then gently released from the hyphae by pipetting and filtered through sterile miracloth. The protoplasts were centrifuged at 3000 x g for 5 minutes, the supernatant discarded and washed with 10 mL of Solution 1 (0.8 M NaCl, 10 mM $CaCl_2$, 50 mM Tris-HCl pH7.5). The pallet was resuspended in 200-500 µL of Solution 1. For each transformation 100 µL were transferred in a 15 tube on ice, 5- 10 µL of plasmid DNA was added to the protoplast and gently mixed and incubated on ice for 2 minutes. After 1 mL of Solution 2 (60 % (w/v) PEG 3350, 0.8 M NaCl, 10 mM $CaCl_2$, 50 mM Tris-HCl pH 7.5) and the tube was incubated at room temperature for 20 minutes. 5 mL of molten (50 °C) MEB containing 0.8 agar and 1 M sorbitol

was added and gently mixed. The mixture was overlaid onto pre-prepared MEA plates containing 1 M sorbitol with approx. 15 mL of medium. The plates were incubated at 25 °C for 1 day. After 1 day 5 mL MEB containing 0.8 % (w/v) and 1 M sorbitol with hygromycin B (50 µg/mL) was overlaid on each plate. The plates were incubated at 25 °C and colonies were checked after 5-6 days.

## 11. Genetic characterisation of the transformants

Transformants from the gene knock-out experiment were used to inoculate 10 mL GN liquid media and cultured at 25 °C shaking at 180 rpm for 5 days. The culture was removed and lyophilized for gDNA extraction. The integration of the hygromycin cassette was tested *via* primers designed outside the homologous regions and within the hygromycin cassette. Primers were also designed to check for genetic purity of the transformants.

### Genetic characterisation of the nrPKS disruption (*dmxPKS*)

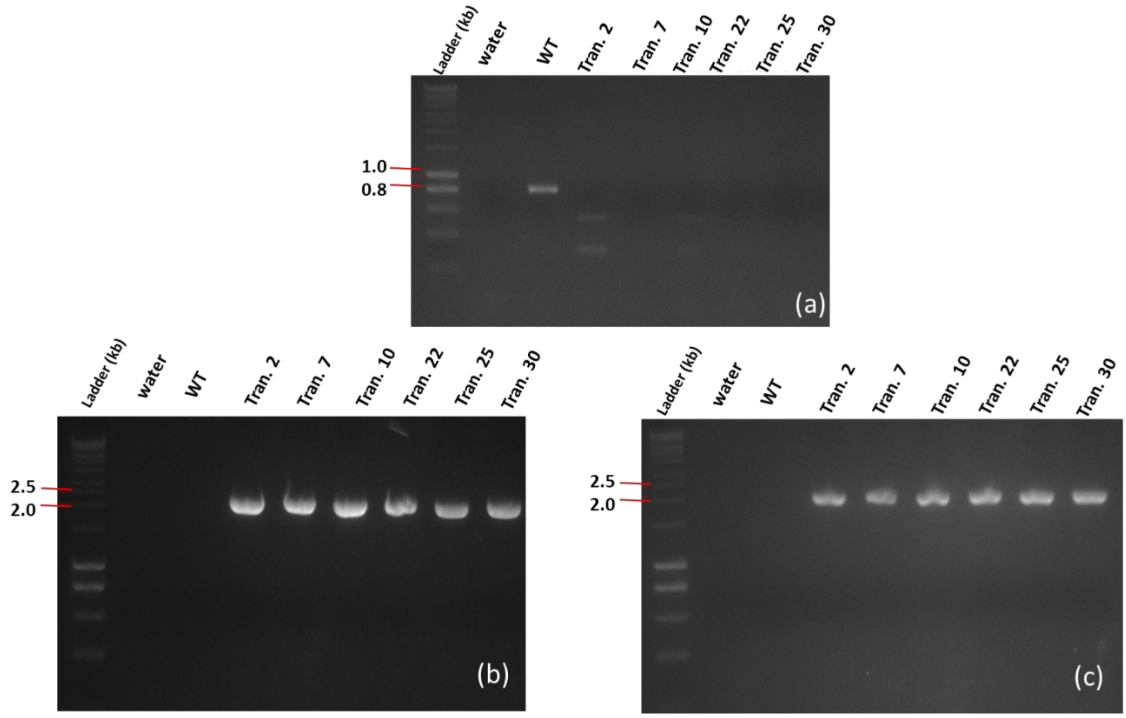


**Figure S3.** PCR analysis of transformants for the *ΔdmxPKS* strain: **A**, the absence of a 869 bp PCR product from *dmxpks* for the transformants demonstrates genetic purity; **B**, a 2063 bp PCR product shows HygR cassette correctly incorporated on LH side; **C**, a 2264 bp PCR product shows HygR cassette correctly incorporated on RH side.

## Genetic characterisation of the P450 dimerase disruption (*dmxR5*)

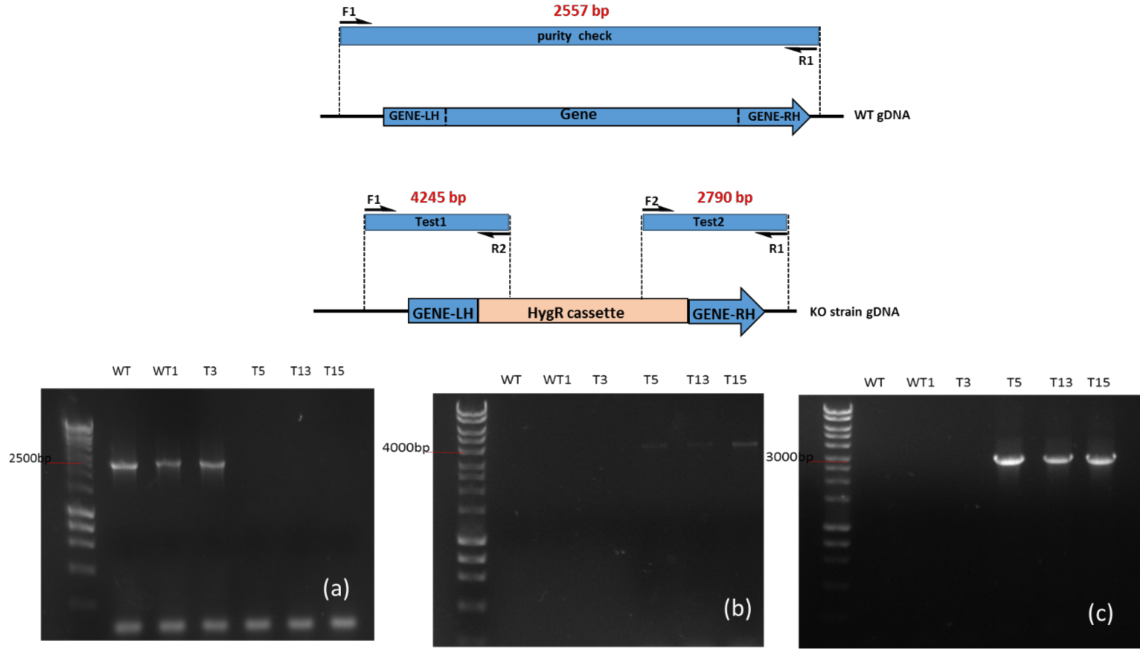


**Figure S4. A**, the transformants T5, T13 and T15 were confirmed to be a genetically pure by the absence of a 2557 bp product for *dmxR5*. **B**, The PCR amplification for the LH side integration was successful, but only produced very faint bands. **C**, PCR amplification showing correct RH side integration was successful showing that hygromycin cassette was correctly incorporated in the *ΔdmxR5* transformants T5, T13 and T15.

## Genetic characterisation of the BVMO disruption (*dmxR6*)

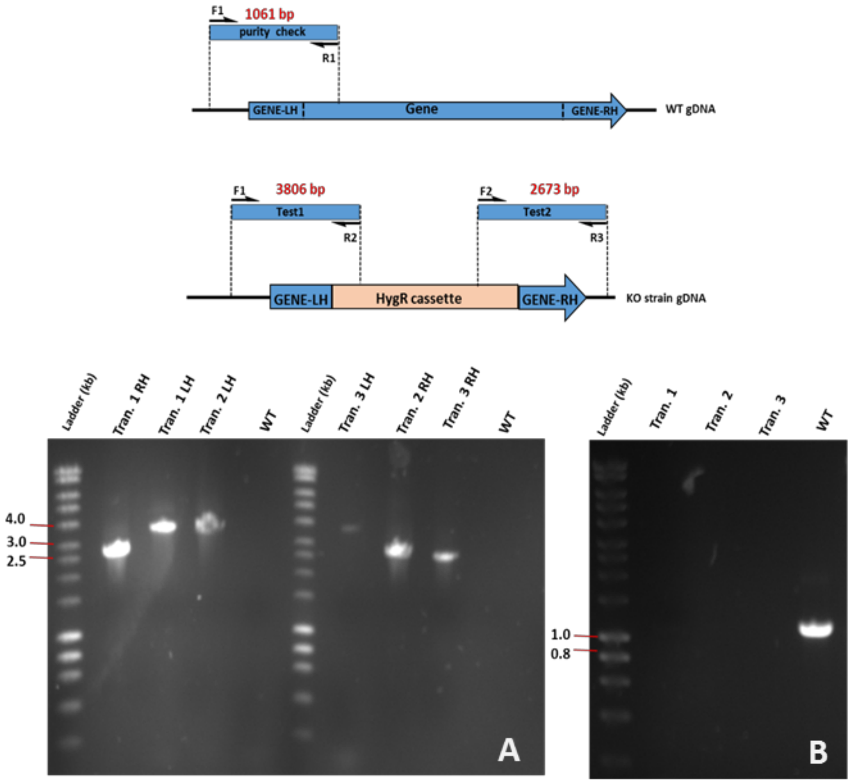


**Figure S5**. PCR analysis of transformants for the *ΔdmxR6* strain: **A**, 3806 bp PCR product shows HygR cassette correctly incorporated on LH side and 2673 bp PCR product shows that the HygR cassette was correctly incorporated on RH side; **B**, 869 bp PCR only for the WT shows that transformants were genetically pure.

## Genetic characterisation of the *O*-acyl transferase (*dmxR13*), the acyl CoA carboxylase (*dmxL1*) and hrPKS (*dmxL2*) disruptions

**Table S5.** PCR analysis confirmed that the three genes (*dmxL2*, *dmxR13* and *dmxL1*) were successfully disrupted.

| Gene targeted | Target | primers | WT product (bp) | KO product (bp) | Number of KO strains |
|---|---|---|---|---|---|
| ***dmxL2*** | Purity | F1+R1 | 2047 | no products | 1 |
| | LH | F2+R3 | no product | 997 | |
| | RH | F2+R2 | no products | 1315 | |
| ***dmxR13*** | Purity | F1+R2 | 2534 | 5905 / no product | 3 |
| | LH | F1+R3 | no product | 1104 | |
| | RH | F2+R2 | no products | 1011 | |
| ***dmxL1*** | Purity | F1+R1 | 2364 | no product | 4 |
| | LH | F2+R3 | no product | 4894 | |
| | RH | F2+R2 | no product | 3852 | |

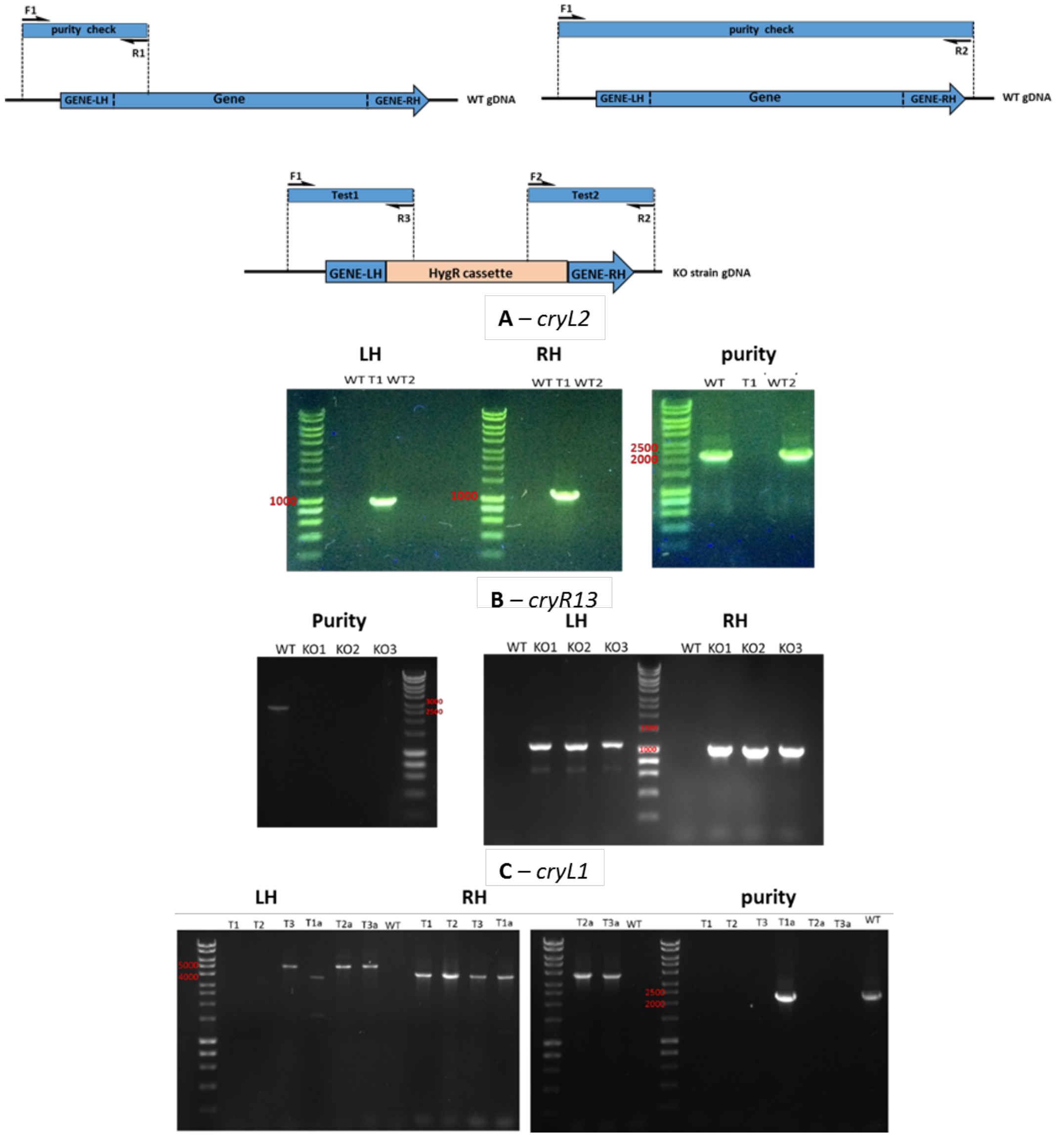


**Figure S6.** PCR analysis confirmed that the three genes were successfully disrupted: **A**, *dmxL2*; **B**, *dmxR13*; **C**, *dmxL1*.

## 12. Mass spectra and UV spectra

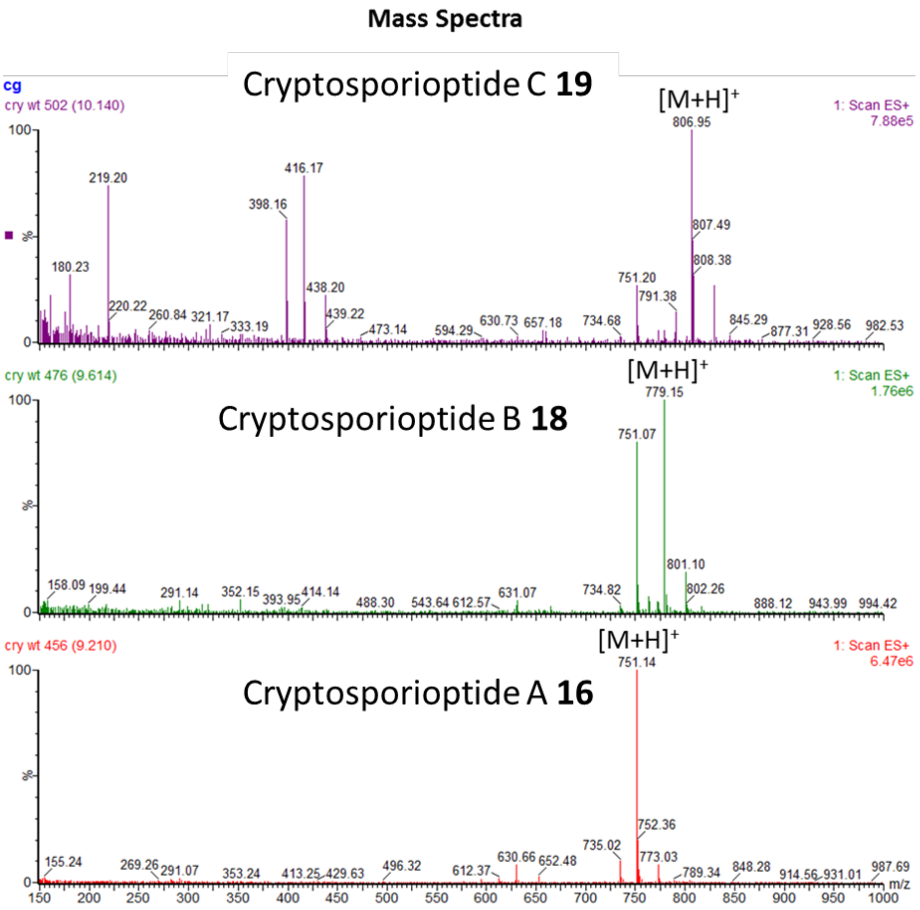


**Figure S7.** $ES^+$ spectra of cryptosporioptide A **16**, cryptosporioptide B **18** and cryptosporioptide C **19**.

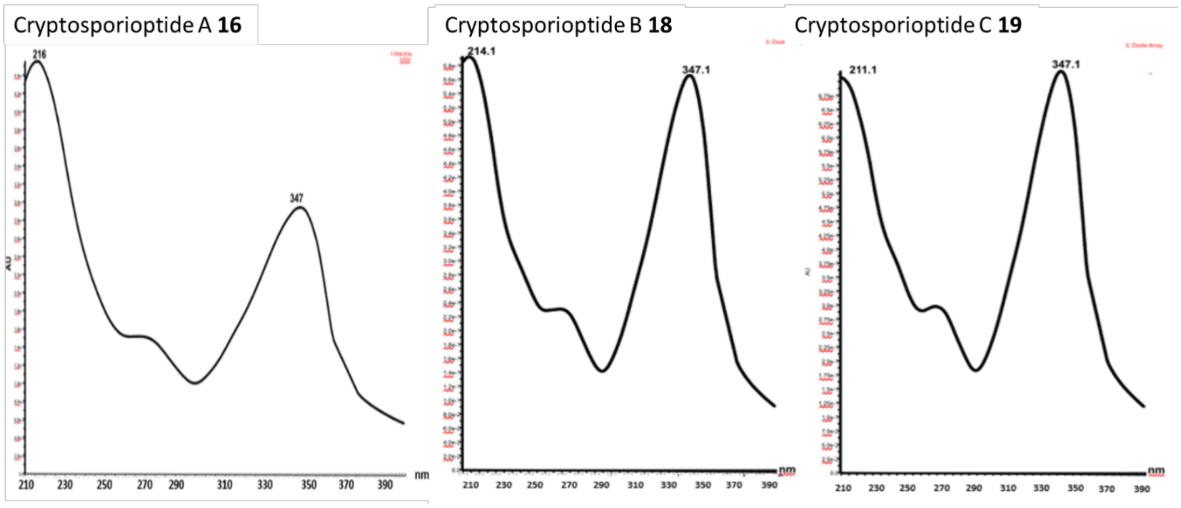


**Figure S8.** UV spectra of cryptosporioptide A **16**, cryptosporioptide B **18** and cryptosporioptide C **19**.

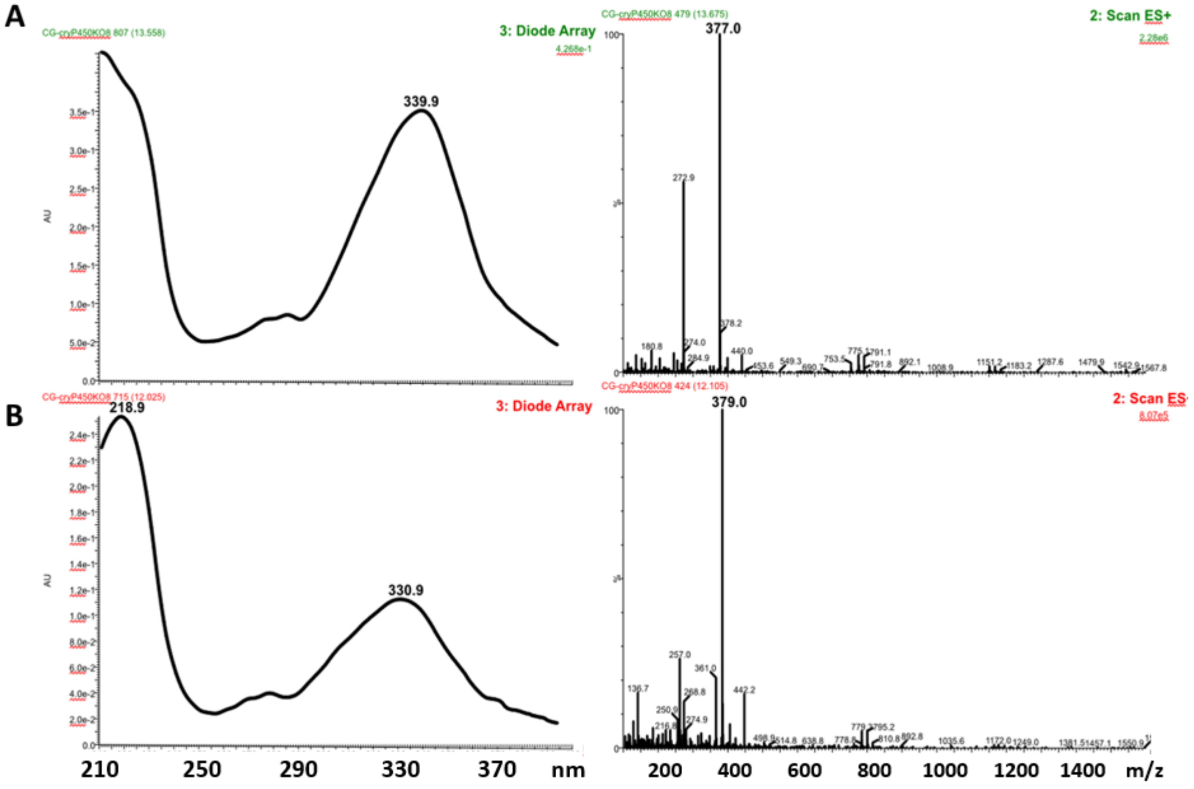


**Figure S9. A,** UV and ES+ spectra of monomer **21**; **B**, UV and ES+ spectra of monomer **22.**

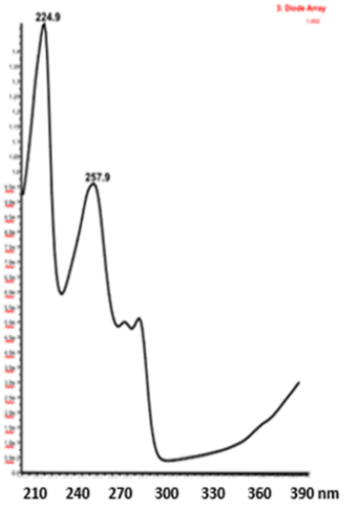


**Figure S10.** UV spectrum of chrysophanol **2**.

## FAB MS of cryptosporioptide dimethyl ester 17

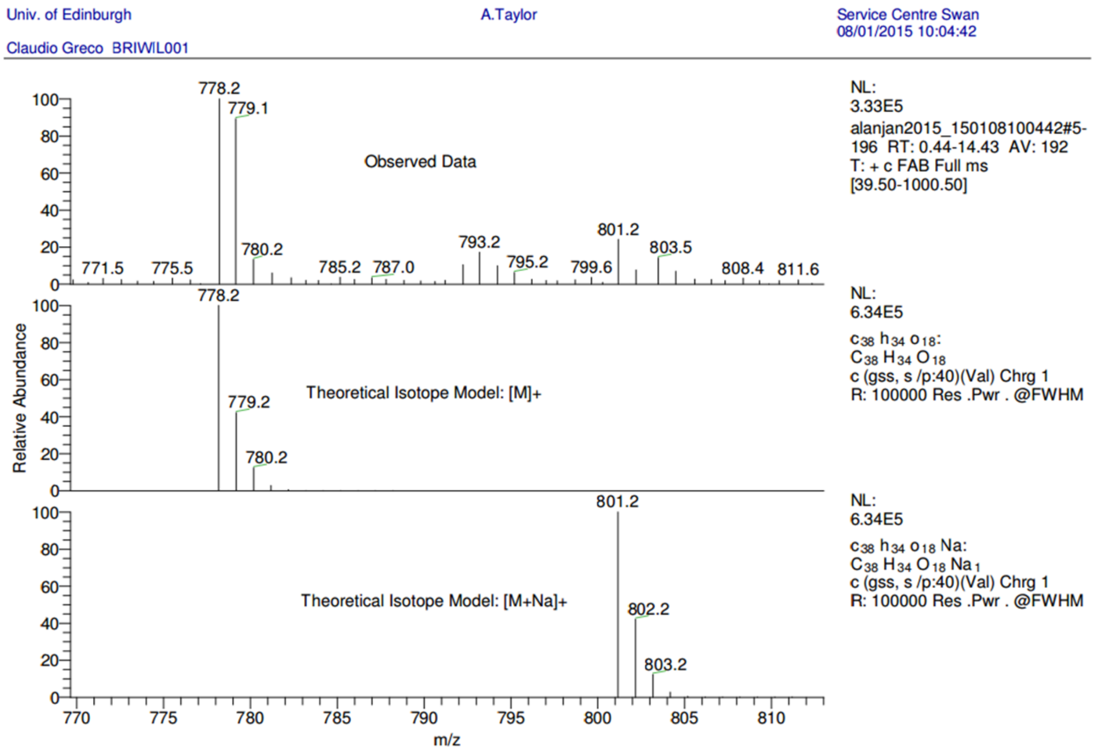


**Figure S11.** FAB-Mass spectrum of cryptosporioptide **17**.

## 13. Structure elucidation and derivatisation

### Cryptosporioptide A 16

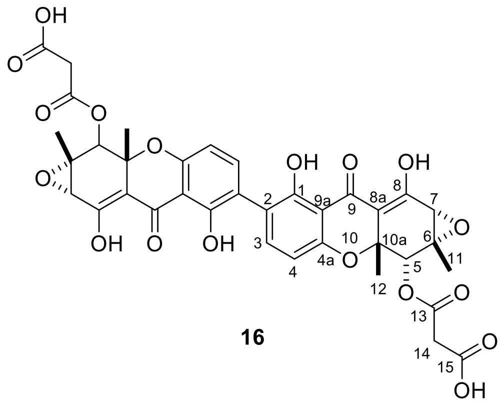

**16**

*Cryptosporiopsis* sp. 8999 was grown on rice (50 g) and extracted after 28 days. The crude extract was purified by preparative HPLC using *Method 4* to obtain cryptosporioptide A **16** as a bright yellow powder (45 mg); $[\alpha]_D^{21}$ +100 (*c* 0.1 MeOH); $\lambda_{max}$ / nm 270, 350; IR cm$^{-1}$ 3500, 2937, 1729, 1611, 1587; $\delta_H$ ($CD_3CN$, 500 MHz) 1.55 (6H, s, 11-$H_3$), 1.60 (6H, s, 12-$H_3$), 3.42 (2H, d, *J* 16.0, 14-$H_a$), 3.55 (2H, s, 7-H), 3.62 (2H, d, *J* 16.0, 14-$H_b$); 5.67 (2H, s, 5-H), 6.38 (2H, d, *J* 8.5, 4-H), 7.37 (2H, d, *J* 8.5, 3-H), 11.71 (2H, s, 1-O*H*), 13.98 (2H, s, 8-O*H*); $\delta_C$ ($CD_3CN$, 125 MHz) 18.2 (C-11), 28.5 (C-12), 41.8 (C-14), 56.3 (C-7), 59.9 (C-6), 73.5 (C-5), 79.9 (C-10a), 105.5 (C-8a), 106.8 (C-9a), 109.0 (C-4), 119.0 (C-2), 141.5 (C-3), 158.4 (C-4a), 159.9 (C-1), 167.7 (C-13), 170.1 (C-15), 171.2 (C-8), 188.9 (C-9); *m/z* (ESI ) 751.1502 $[M+H]^+$ ($C_{36}H_{31}O_{18}$ requires 751.1432).

**Cryptosporioptide dimethyl ester 17**

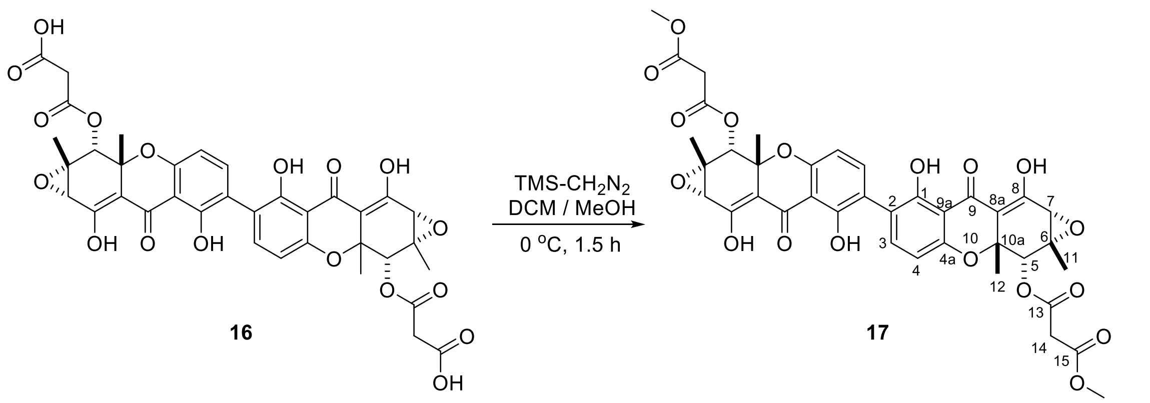


To cryptosporioptide A **16** (10.4 mg, 0.014 mmol, 1 eq.) in methanol (12 µL) and DCM (40 µL) was added dropwise TMS-CHN$_2$ 2 M solution in diethyl ether (17 µL, 0.033 mmol, 2.3 eq.) at 0 °C. After stirring for 1.5 h, the reaction was allowed to warm up to room temperature and quenched by adding a drop of acetic acid. The solvents were removed under a stream of nitrogen to obtain cryptosporioptide dimethyl ester **17** as a bright yellow powder (10.4 mg, 0.014 mmol, 95%);[18,19] $[\alpha]_D^{21}$ +210 (*c* 0.1 MeOH); $\lambda_{max}$ / nm 270, 350; $\delta_H$ ($CDCl_3$, 500 MHz) 1.59 (6H, s, 11-$H_3$), 1.65 (6H, s, 12-$H_3$), 3.39 (2H, d, *J* 16.0, 14-H*H*), 3.42 (2H, s, 7-H), 3.44 (2H, d, *J* 16.0, 14-*H*H), 3.65 (6H, s, 16-$H_3$); 5.64 (2H, s, 5-H), 6.43 (2H, d, *J* 8.5, 4-H), 7.40 (2H, d, *J* 8.5, 3-H), 11.69 (2H, s, 1-O*H*), 14.02 (2H, s, 8-O*H*); $\delta_C$ ($CDCl_3$, 125 MHz) 18.2 (C-11), 28.6 (C-12), 41.3 (C1-4), 52.7 (C-16), 56.0 (C-7), 58.8 (C-6), 73.3 (C-5), 78.9 (C-10a), 104.4 (C8a), 106.1 (C-9a), 108.4 (C-4), 117.7 (C-2), 140.6 (C-3), 157.2 (C-4a), 159.2 (C-1), 165.5 (C-13), 166.7 (C-15), 170.0 (C-8), 187.9 (C-9); *m/z* (ESI) 779.1828 $[M+H]^+$ ($C_{38}H_{35}O_{18}$ requires 779.1745).

**Cryptosporioptide B 18**

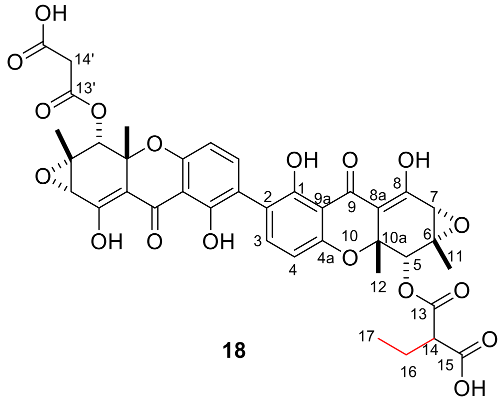


*Cryptosporiopsis* sp. 8999 was grown on rice (50 g) and extracted after 28 days. The crude extract was purified by preparative HPLC using *Method 4* to obtain cryptosporioptide B **18** as a bright yellow powder (1.8 mg); $[\alpha]_D^{21}$ +110 (*c* 0.1 MeOH); $\lambda_{max}$ / nm 270, 350; $\delta_H$ ($CD_3CN$, 500 MHz) 0.85 (3H, m, 17-$H_3$), 1.51 (3H, s, 11'-$H_3$), 1.52 (3H, s, 11-$H_3$), 1.60 (6H, s, 12-$H_3$), 1.81 (2H, m, 16-$H_2$), 3.29 (1H, m, 14-H), 3.35 (1H, d, *J* 16.0, 14'-*H*H), 3.39 (1H, d, *J* 16.0, 14'-H*H*), 3.53 (2H, s, 7-H), 5.67 (2H, s, 5-H), 6.35 (2H, d, *J* 8.5, 4-H), 7.40 (2H, d, *J* 8.5, 3-H); $\delta_C$ ($CD_3CN$, 125 MHz) 12.0 (C-17), 18.0 (C-11), 23.0 (C-16), 28.5 (C-12), 53.8 (C-14'), 56.6 (C-7), 59.8 (C-6), 73.4 (C-5), 79.9 (C-

10a), 105.0 (C-8a), 106.9 (C-9a), 108.9 (C-4), 118.7 (C-2), 141.2 (C3), 157.9 (C4a), 159.9 (C1), 169.7 (C13), 169.3 (C-13'), 172.0 (C-8), 188.3 (C-9), 41.9 (C-14), 170.8 (C-15), 170.3 (C-15'); *m/z* (ESI) 779.1823 $[M+H]^+$ ($C_{38}H_{35}O_{18}$ requires 779.1745).

**Cryptosporioptide C 19**

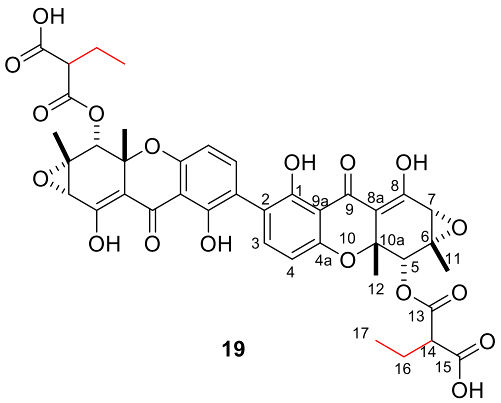

**19**

*Cryptosporiopsis* sp. 8999 was grown on rice (50 g) and extracted after 28 days. The crude extract was purified by preparative HPLC using *Method 4* to obtain cryptosporioptide C **19** as a bright yellow powder (5 mg); $[\alpha]_D^{21}$ +40° (*c* 0.1 MeOH); $\lambda_{max}$ / nm) 270, 350; $\delta_H$ ($CD_3CN$, 500 MHz) 0.85 (6H, m, 17-$H_3$), 1.51 (6H, s, 11-$H_3$), 1.59 (6H, s, 12-$H_3$), 1.81 (4H, m, 16-$H_2$), 3.29 (2H, m, 14-H), 3.50 (2H, s, 7-H), 5.69 (2H, s, 5-H), 6.33 (2H, d, *J* 8.5, 4-H), 7.37 (2H, d, *J* 8.5, 3-H), 11.96 (2H, s, 1-OH); $\delta_C$ ($CD_3CN$, 125 MHz) 12.0 (C-17), 18.2 (C-11), 23.0 (C-16), 28.5 (C-12), 54.4 (C-14), 56.8 (C-7), 59.8 (C-6), 73.4 (C-5), 80.0 (C-10a), 104.8 (C-8a), 106.9 (C-9a), 108.9 (C-4), 118.2 (C-2), 141.1 (C-3), 157.7 (C-4a), 159.9 (C-1), 169.7 (C-13), 170.3 (C-15), 170.8 (C-8), 188.0 (C-9); *m/z* (ESI) 807.2137 $[M+H]^+$ ($C_{40}H_{39}O_{18}$ requires 807.2058).

**Cryptosporioptide monomer 21**

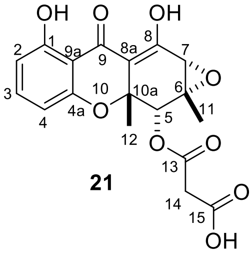

**21**

*Cryptosporiopsis ΔdmxR5* strain was grown on rice (10 g) and extracted after 28 days. The crude extract was purified by preparative HPLC using *Method 4* to obtain cryptosporioptide monomer **21** as a bright yellow powder (28 mg); $\lambda_{max}$ / nm 270, 350; $[\alpha]_D^{21}$ +50 (c 0.1 MeOH); $\delta_H$ ($CD_3CN$, 500 MHz) 1.53 (3H, s, 12-$H_3$), 1.56 (3H, s, 11-$H_3$), 3.32 (1H, d, *J* 16.0, 14-H*H*), 3.37 (1H, d, *J* 16.0, 14-*H*H), 3.52 (1H, s, 7-H), 5.64 (1H, s, 5-H), 6.47 (1H, d, *J* 8.3, 4-H), 6.33 (1H, d, *J* 8.5, 2-H), 7.37 (1H, t, *J* 8.5, 3-H), 11.21 (1H, s, 1-O*H* ); $\delta_C$ ($CD_3CN$, 125 MHz) 18.0 (C-12), 28.4 (C-11), 41.8 (C-14), 79.7 (C-10a), 59.4 (C-7), 59.8 (C-6), 73.6 (C-5), 105.1 (C-8a), 107.1 (C-9a), 109.4 (C-2), 110.4 (C-4), 139.4 (C-3), 158.6 (C-1), 162.6 (C-4a), 167.9 (C-13), 171.1 (C-15), 171.7 (C-8), 188.5 (C-9); *m/z* (ESI) 399.0672 $[M+Na]^+$ ($C_{18}H_{16}NaO_9$ requires 399.0687).

**Cryptosporioptide monomer 22a and 22b**

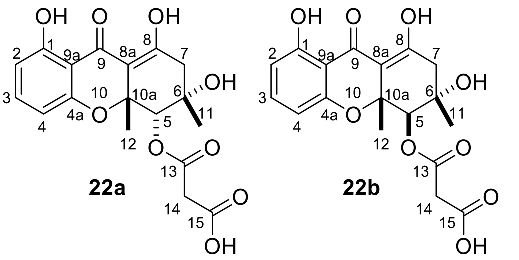

**22a** **22b**

*Cryptosporiopsis ΔdmxR5* strain was grown on rice (10 g) and extracted after 28 days. The crude extract was purified by preparative HPLC using *Method 4* to obtain a mixture of cryptosporioptide monomer **22a** and monomer **22b** as a bright yellow powder (15 mg in a 3:1 mixture); $\lambda_{max}$ / nm 270, 350; *m/z* (ESI) 401.0852 $[M+Na]^+$ ($C_{18}H_{18}NaO_9$ requires 401.0843).

Monomer **22a**: $\delta_H$ ($CD_3CN$, 500 MHz) 1.33 (3H, s, 11-$H_3$), 1.70 (3H, s, 12-$H_3$), 2.41 (1H, d, *J* 19.0, 7-*H*H), 2.74 (1H, d, *J* 19.0, 7-H*H*), 3.32 (1H, d, *J* 16.0, 14-H*H*), 3.37 (1H, d, *J* 16.0, 14-*H*H), 5.18 (1H, s, 5-H), 6.30 (1H, d, *J* 8.0, 2-H), 6.46 (1H, d, *J* 8.0, 4-H), 7.36 (1H, t, *J* 8.0, 3-H), 11.36 (1H, s, 1-OH); $\delta_C$ ($CD_3CN$, 125 MHz) 26.0 (C-12), 27.4 (C-11), 40.8 (C-7), 42.1 (C-14), 71.1 (C-6), 77.3 (C-5), 80.9 (C-10a), 105.3 (C-8a), 107.7 (C-9a), 109.5 (C-2), 110.1 (C-4), 139.1 (C-3), 159.2 (C-1), 162.8 (C-4a), 167.0 (C-13), 168.2 (C-15), 175.4 (C-8), 189.2 (C-9).

Monomer **22b**: $\delta_H$ ($CD_3CN$, 500 MHz) 1.32 (3H, s, 11-$H_3$), 1.50 (3H, s, 12-$H_3$), 2.64 (1H, d, *J* 19.0, 7-H*H*), 2.80 (1H, d, *J* 19.0, 7-*H*H), 3.40 (1H, d, *J* 16.0, 14-H), 3.55 (1H, d, *J* 15.0, 14-H), 5.53 (1H, s, 5-H), 6.33 (1H, d, *J* 8.0, 2-H), 6.48 (1H, d, *J* 8.0, 4-H),7.38 (1H, t, *J* 8.0, 3-H); $\delta_C$ ($CD_3CN$, 125 MHz) 20.9 (C-12), 24.6 (C-11), 43.5 (C-14), 43.7 (C-7), 70.7 (C-6), 80.3 (C-5), 81.2 (C-10a), 106.8 (C-8a), 108.2 (C-9a), 109.4 (C-2), 110.5 (C-4), 139.3 (C-3), 159.0 (C-1), 163.0 (C-4a), 168.2 (C-13), 170.4 (C-15), 173.7 (C-8), 189.5 (C-9).

**Chrysophanol 2**

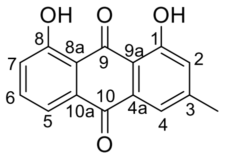

**2**

*Cryptosporiopsis ΔdmxR6* strain was grown on rice (10 g) and extracted after 28 days. The crude extract was purified by preparative HPLC using *Method 4* to obtain chrysophanol **2** as orange needles (22 mg); $\lambda_{max}$ / nm 225, 257, 290; $\delta_H$ (500 MHz, $CDCl_3$) 12.13 (1H, s, 8-OH), 12.02 (1H, s, 1-OH), 7.83 (1H, dd, 7.5, 1.0, 5-H), 7.67 (1H, dd, 8.5, 7.5, 6-H), 7.66 (1H, m, 4-H), 7.30 (1H, dd, 8.5, 1.0, 7-H), 7.11 (1H, m, 2-H), 2.47 (3H, s, Me); $\delta_C$ (125 MHz, $CDCl_3$) 192.7 (C-9), 182.2 (C-10), 162.9 (C-8), 162.6 (C-1), 149.5 (C-3), 137.1 (C-6), 133.8 (C-5a), 133.5 (C-4a), 124.7 (C-7), 124.5 (C-2), 121.5 (C-4), 120.1 (C-5), 116.1 (C-8a), 113.9 (C-9a), 22.4 (-$CH_3$); *m/z* (ESI) 255.0571 $[M+H]^+$ ($C_{15}H_{11}O_4$ requires 255.0579). Data in accordance to the literature.[20,21]

## 14. NMR data

**Table 6.** NMR assignments of cryptosporioptides **16**, **18** and **19** ([a]500 MHz, [b]126 MHz in $CD_3CN$).

| | Cryptosporioptide A 16 | | | Cryptosporioptide B 18 | | | Cryptosporioptide C 19 | | |
|---|---|---|---|---|---|---|---|---|---|
| **Position** | **$\delta_C^a$ / ppm** | **$\delta_H^b$ / ppm (*J* /Hz)** | **HMBC** | **$\delta_C^a$ / ppm** | **$\delta_H^b$ / ppm (*J* / Hz)** | **HMBC** | **$\delta_C^a$ / ppm** | **$\delta_H^b$ / ppm (*J* / Hz)** | **HMBC** |
| **C-1** | 159.2 | - | | 159.9 | - | | 160.0 | - | |
| **C-1-OH** | - | - | | - | - | | - | - | |
| **C-2** | 117.6 | - | | 118.7 | - | | 118.2 | - | |
| **C-3** | 140.6 | 7.37 (2H, d, 8.5) | C-1, C-2, C-4a | 141.2 | 7.40 (2H, two d, 8.5) | C-1, C-2, C-4a | 141.1 | 7.37 (2H, two d, 8.5) | C-1, C-2, C-4a |
| **C-4** | 108.4 | 6.38 (2H, d, 8.5) | C-2, C-4a, C- 9, C-9a | 108.9 | 6.35 (2H, m) | C-2, C-4a, C- 9, C-9a | 108.9 | 6.33 (2H, two d, 8.5) | C-2, C-4a, C- 9, C-9a |
| **C-4a** | 157.2 | - | | 157.9 | - | | 157.7 | - | |
| **C-5** | 73.3 | 5.67 (2H, s) | C-6, C-7, C-8a, C-10a, C-11, C-12 C-13 | 73.4 | 5.67 (2H, two s) | C-6, C-7, C-8a, C-10a, C-11, C-12, C-13 | 73.4 | 5.69 (1H, s) / 5.67 (1H, s) | C-6, C-7, C-8a, C-10a, C-11,C-12, C-13 |
| **C-6** | 58.8 | - | | 59.8 | - | | 59.8 | - | |
| **C-7** | 56.0 | 3.55 (2H, s) | C-6, C-8a, C-12, C-8 | 56.6 | 3.53 (2H, s) | C-6, C-8a, C-12, C-8 | 56.8 | 3.50 (2H, s) | C-6, C-8a, C-12, C-8 |
| **C-8** | 170.0 | - | | 172.0 | | | 170.8 | 9- | |
| **C-8-OH** | - | - | | - | - | | - | - | |
| **C-8a** | 104.4 | - | | 105.0 | - | | 104.8 | - | |
| **C-9** | 187.9 | - | | 188.3 | - | | 188.0 | - | |
| **C-9a** | 106.1 | - | | 106.9 | - | | 106.9 | - | |
| **C-10a** | 78.9 | - | | 79.7 | - | | 80.0 | - | |
| **C-11** | 18.2 | 1.55 (6H, s) | C-5, C-6, C-7, C-10a | 18.0 | 1.52/1.51 (6H two s) | C-5, C-6, C-7, C-10a | 18.1 | 1.51 (6H, s) | C-5, C-6, C-7, C-10a |
| **C-12** | 28.6 | 1.60 (6H, s) | C-5, C-8a, C-10a | 28.5 | 1.60 (6H, s) | C-5, C-8a, C-10a | 28.5 | 1.59 (6H, s) | C-5, C-8a, C-10a |
| **C-13** | 165.5 | - | | 169.7 | - | | 169.7 | - | |
| **C-13'** | - | - | | 169.3 | - | | | | |
| **C-14** | 41.3 | 3.42 (2H, d, 16.0) 3.62 (2H, d, 16.0) | | 53.8 | 3.29 (1H, m) | C-13 | 54.4 | 3.29 (2H, m) | |
| **C-14'** | - | - | | 41.9 | (1H, 3.39, d, 16.0) (1H, 3.35, d, 16.0) | C-13' | - | - | |
| **C-15** | 166.7 | - | | 170.8 | - | | 170.3 | - | |
| **C-15'** | - | - | | 170.3 | - | | | | |
| **C-16** | - | - | | 23.0 | 1.81 (2H, m) | C-14, C-17 | 23.0 | 1.81 (4H m) | C-14, C-17 |
| **C-17** | - | - | | 12.0 | 0.85 (3H. m) | | 12.0 | 0.85 (6H m) | |

**Table S7.** NMR data comparison of cryptosporioptide dimethyl ester **17** and reported cryptosporioptide **12** ([a]500 MHz, [b]126 MHz in $CDCl_3$).

| | Cryptosporioptide Dimethyl Ester **17** | | | | Cryptosporioptide **12**[5] | |
|---|---|---|---|---|---|---|
| **Position** | [b]$\delta_C$ | [a]$\delta_H$ (*J* in Hz) | HMBC | NOE | [b]$\delta_C$ | [a]$\delta_H$ (*J* in Hz) |
| **C1** | 159.2 | - | - | | 158.9 | - |
| **C1-OH** | | 11.69 (2H, s) | - | C3 | | 11.78 (s) |
| **C2** | 117.7 | - | - | | 117.1 | - |
| **C3** | 140.6 | 7.40 (2H, d, 8.5) | C1, C2, C4a | C1-OH | 140.4 | 7.40 (d, 8.5) |
| **C4** | 108.4 | 6.43 (2H, d, 8.5) | C2, C4a, C9, C9a | C11 | 108.4 | 6.43 (d, 8.5) |
| **C4a** | 157.2 | - | | | 157.1 | |
| **C5** | 73.3 | 5.64 (2H, s) | C6, C7, C8a, C9, C10a, C11, C12, C13 | C6 | 73.1 | 5.62 (s) |
| **C6** | 58.8 | - | - | C5, C7 | 58.6 | - |
| **C7** | 56.0 | 3.42 (2H, s) | C6, C8, C8a C12, | C6 | 55.8 | 3.42 (s) |
| **C8** | 170.0 | - | - | - | 169.8 | - |
| **C8-OH** | | 14.02 (2H, s) | - | - | | 14.12 (s) |
| **C8a** | 104.4 | - | - | - | 104.2 | - |
| **C9** | 187.9 | - | - | - | 187.7 | - |
| **C9a** | 106.1 | - | - | - | 105.9 | - |
| **C10a** | 78.9 | - | - | - | 78.8 | - |
| **C11** | 18.2 | 1.59 (6H, s) | C5, C6, C7 | - | 18.0 | 1.60 (s) |
| **C12** | 28.6 | 1.65 (6H, s) | C5, C8a, C10a, | C4, C6 | 28.4 | 1.70 (s) |
| **C13** | 165.5 | - | - | - | 165.3 | - |
| **C14** | 41.3 | 3.39 (2H, d, 15.8)<br>3.44 (2H, d, 15.8) | C13, C15 | - | 41.1 | 3.39 (d, 15.5)<br>3.44 (d, 15.5) |
| **C15** | 166.7 | | - | - | 166.5 | - |
| **C16** | 52.7 | 3.65 (6H, s) | C15 | | 52.5 | 3.66 (s) |

**Table S8**. NMR assignments of cryptosporioptide monomers **22a, 22b** and **21** ([a]500 MHz, [b]126 MHz in $CD_3CN$).

| Position | Monomer xanthone 22a | | | | Monomer xanthone 22b | | | | Monomer xanthone 21 | | | |
|---|---|---|---|---|---|---|---|---|---|---|---|---|
| | $\delta_C$[1] / ppm | $\delta_H$[2] / ppm (*J* / Hz) | HMBC | COSY | $\delta_C$[1] / ppm | $\delta_H$[2] / ppm (*J* / Hz) | HMBC | COSY | $\delta_C$[1] / ppm | $\delta_H$[2] / ppm (*J* / Hz) | HMBC | COSY |
| **C-1** | 159.2 | - | - | - | 159.0 | - | - | - | 158.6 | - | - | - |
| **C1-OH** | - | 11.36 | - | - | | - | - | - | | 11.21 (1H, s) | - | - |
| **C-2** | 109.5 | 6.30 (1H, d, 8.0) | C-1, C-4, C-9a, C-9 | C-3 | 109.4 | 6.33 (1H, d, 8.0) | C-1, C-4, C-9a, C-9 | C-3 | 109.4 | 6.33 (1H, d, 8.0) | C-1, C-4, C-4a C-9a, | C-3 |
| **C-3** | 139.1 | 7.36 (1H, app. t, 8.0) | C-1, C-4a, | C-2, C-4 | 139.3 | 7.38 (1H, app. t, 8.0) | C-1, C-4a, | C-2, C-4 | 139.4 | 7.37 (1H, app. t, 8.0) | C-1, C-2, C-4a, C-9a | C-2, C-4 |
| **C-4** | 110.1 | 6.46 (1H, d, 8.0) | C-2, C-4a, C-9a | C-3 | 110.5 | 6.48 (1H, d, 8.0) | C-2, C-4a | C-3 | 110.4 | 6.47 (1H, d, 8.0) | C-2, C-4a, C-9, C-9a | C-3 |
| **C-4a** | 162.8 | - | | | 163.0 | - | | - | 162.6 | - | - | - |
| **C-5** | 77.3 | 5.18 (1H, s) | C-6, C-7, C-9, C-10a, C-11, C-12, C-13 | | 80.3 | 5.53 (1H, s) | C-6, C-7, C-10a, C-11, C-12, C-13 | - | 73.6 | 5.64 (1H, s) | C-6, C-7, C-8, C-8a C-9, C-11, C-12, C-13 | - |
| **C-6** | 71.1 | - | | | 70.7 | - | | | 59.8 | - | - | - |
| **C-7** | 40.8 | 2.41 (1H, d, 19.0) 2.74 (1H, d, 19.0) | C-5, C-6, C-8a, C-12, C-8 | C-7’ C-7’’ | 43.7 | 2.64 (1H, d, 19.0) 2.80 (1H, d, 19.0) | C-6, C-8a, C-12, C-8 | C-7’ C-7’’ | 56.4 | 3.52(1H, s) | C-6, C-8a, C-12, C-8 | |
| **C-8** | 175.4 | - | - | - | 173.7 | - | - | - | 171.7 | - | - | - |
| **C-8a** | 105.3 | - | - | - | 106.8 | - | - | - | 105.1 | - | - | - |
| **C-9** | 189.2 | - | - | - | 189.5 | - | - | - | 188.5 | - | - | - |
| **C-9a** | 107.7 | - | - | - | 108.2 | - | - | - | 107.4 | - | - | - |
| **C-10a** | 80.9 | - | - | - | 81.2 | - | - | - | 79.7 | - | - | - |
| **C-11** | 27.4 | 1.33 (3H, s) | C-5, C-6, C-7, C-8 | - | 24.6 | 1.32 (3H, s) | C-5, C-6, C-7 | - | 18.0 | 1.53 (3H, s) | C-5, C-6, C-7 | - |
| **C-12** | 26.0 | 1.70 (3H, s) | C-5, C-8a, C-10a | - | 20.9 | 1.50 (3H, s) | C-5, C-8a | - | 28.4 | 1.56 (3H, s) | C-5, C-8a, C-10a | - |
| **C-13** | 167.0 | - | - | - | 168.2 | - | - | - | 167.9 | - | - | - |
| **C-14** | 42.1 | 3.37 (1H, d, 16.0) 3.32 (1H, d, 16.0) | C-13, C-15 | C-14 C-14’ | 43.5 | 3.40 (1H, d, 16.0) 3.55 (1H, d, 16.0) | C-13, C-15 | C-14 C-14’ | 41.8 | 3.37 (1H, d, 16.0) 3.32 (1H, d, 16.0) | C-13, C-15 | |
| **C-15** | 168.2 | - | - | - | 170.4 | - | - | - | 171.1 | | | |

Cryptosporioptide A **16**

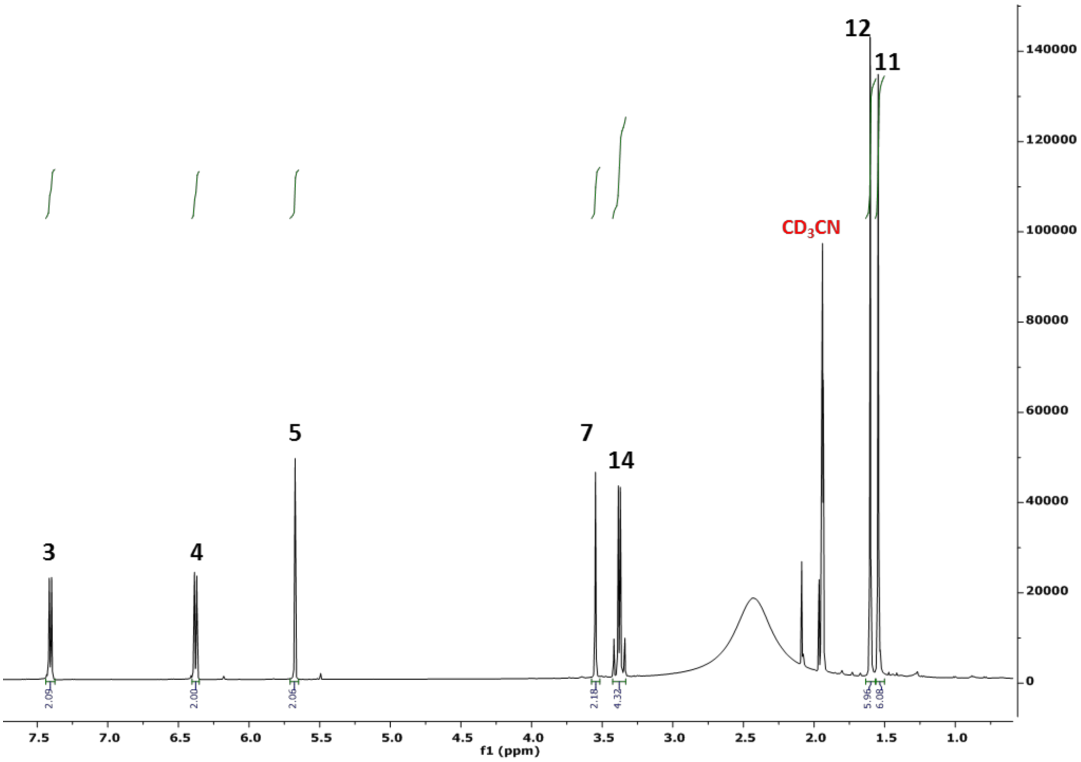


**Figure S12:** $^{1}H$ NMR of cryptosporioptide A **16** (500 MHz in $CD_3CN$).

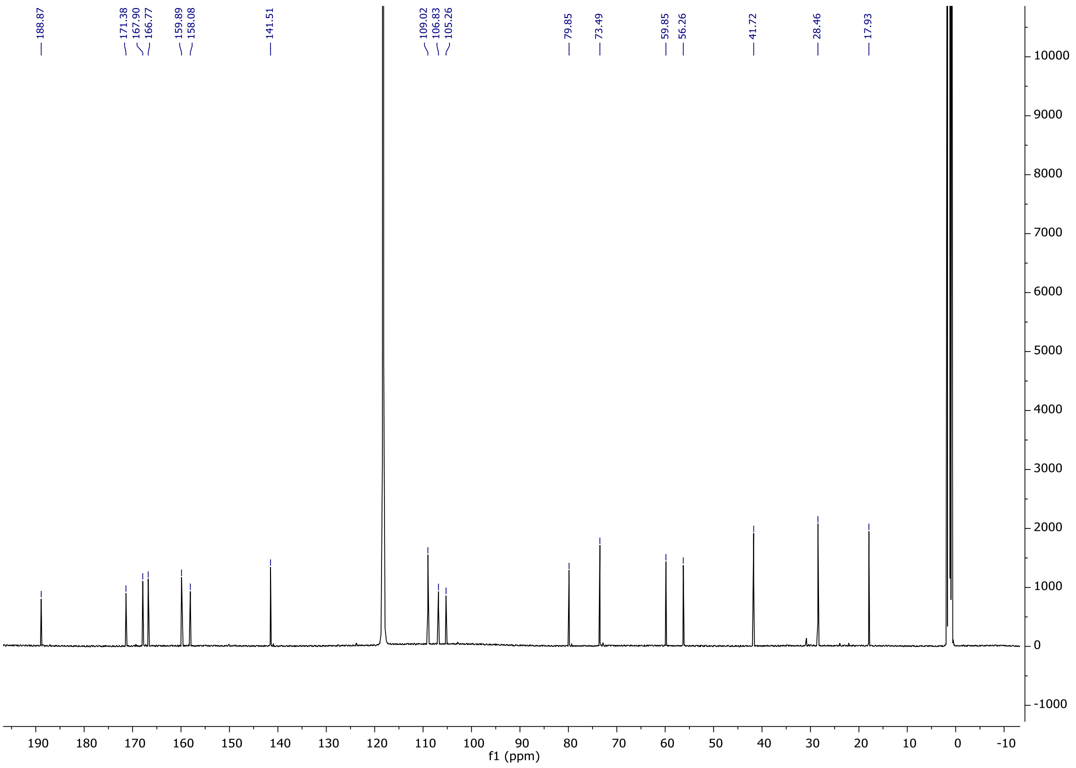


**Figure S13:** $^{13}C$ NMR of cryptosporioptide A **16** (126 MHz in $CD_3CN$).

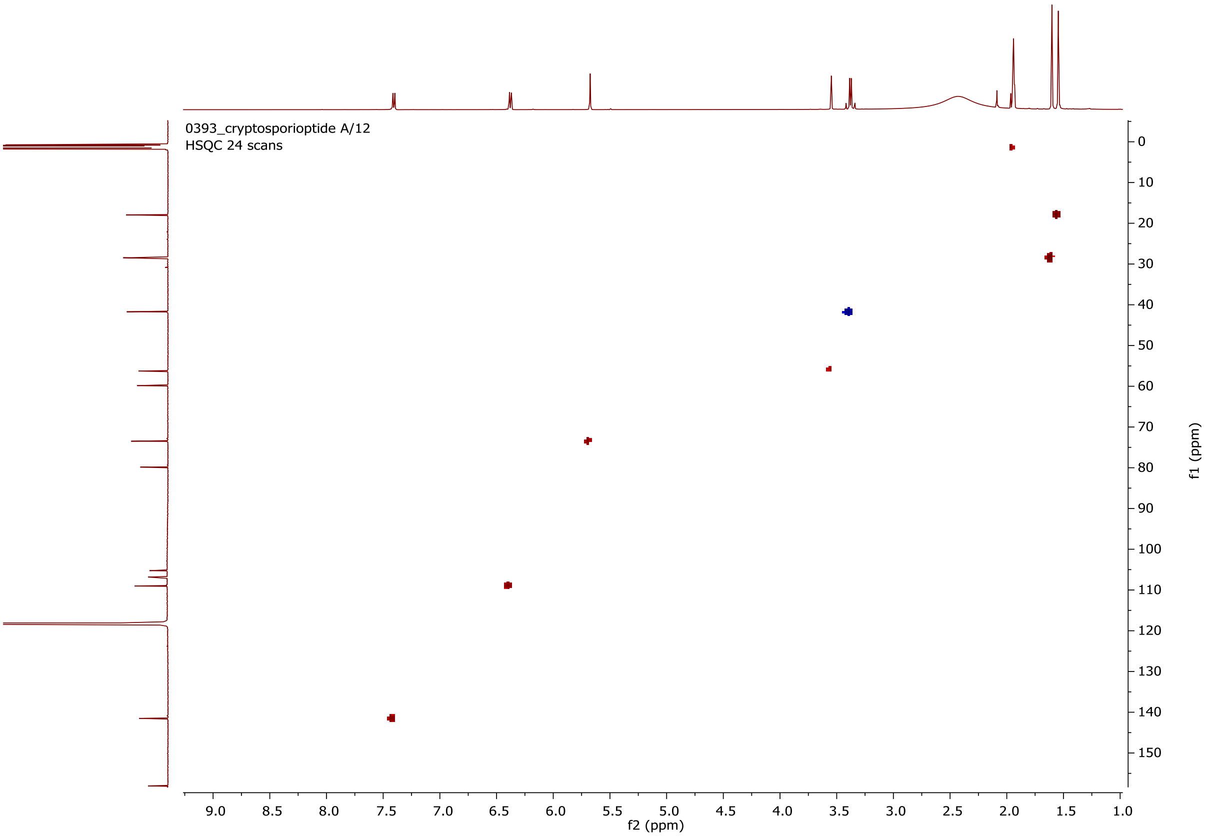


**Figure S14:** HSQC of cryptosporioptide A **16** (500 MHz in $CD_3CN$).

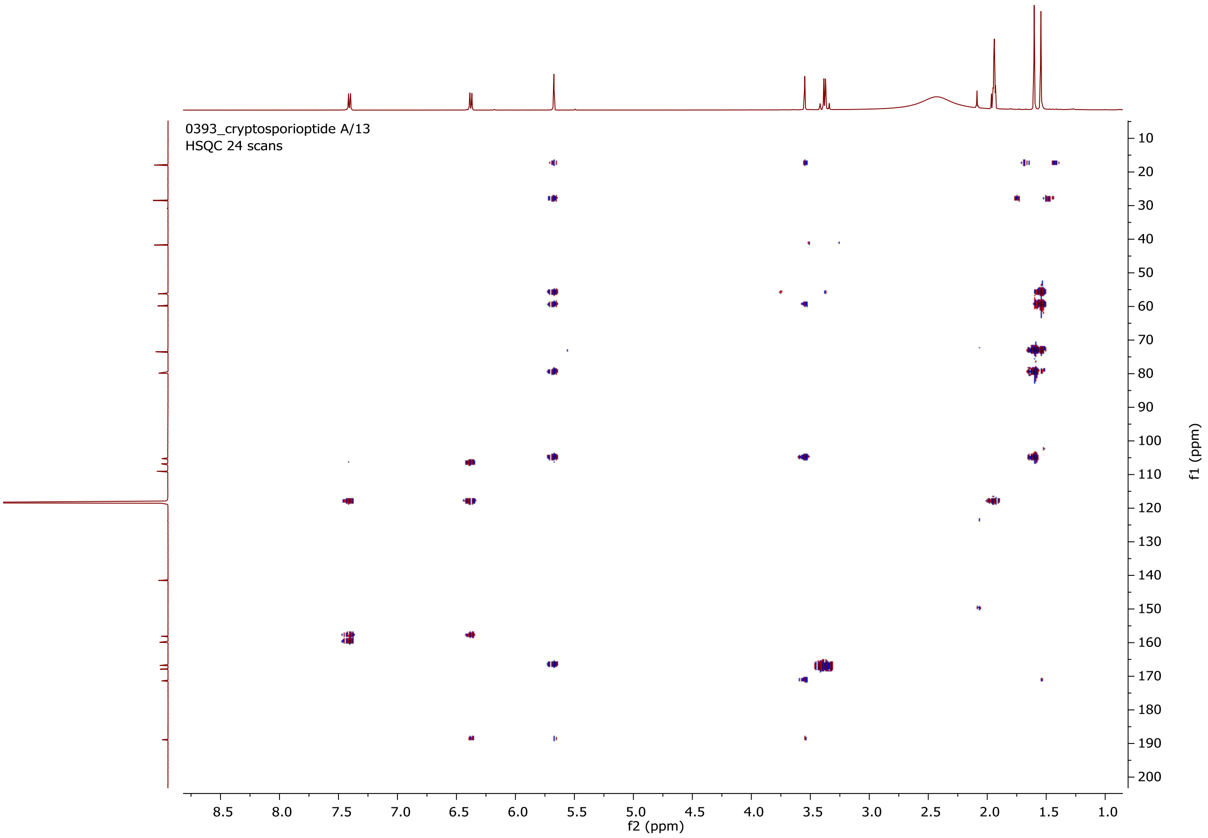


**Figure S15:** HMBC of cryptosporioptide A **16** (500 MHz in $CD_3CN$).

## Cryptosporioptide dimethyl ester 17

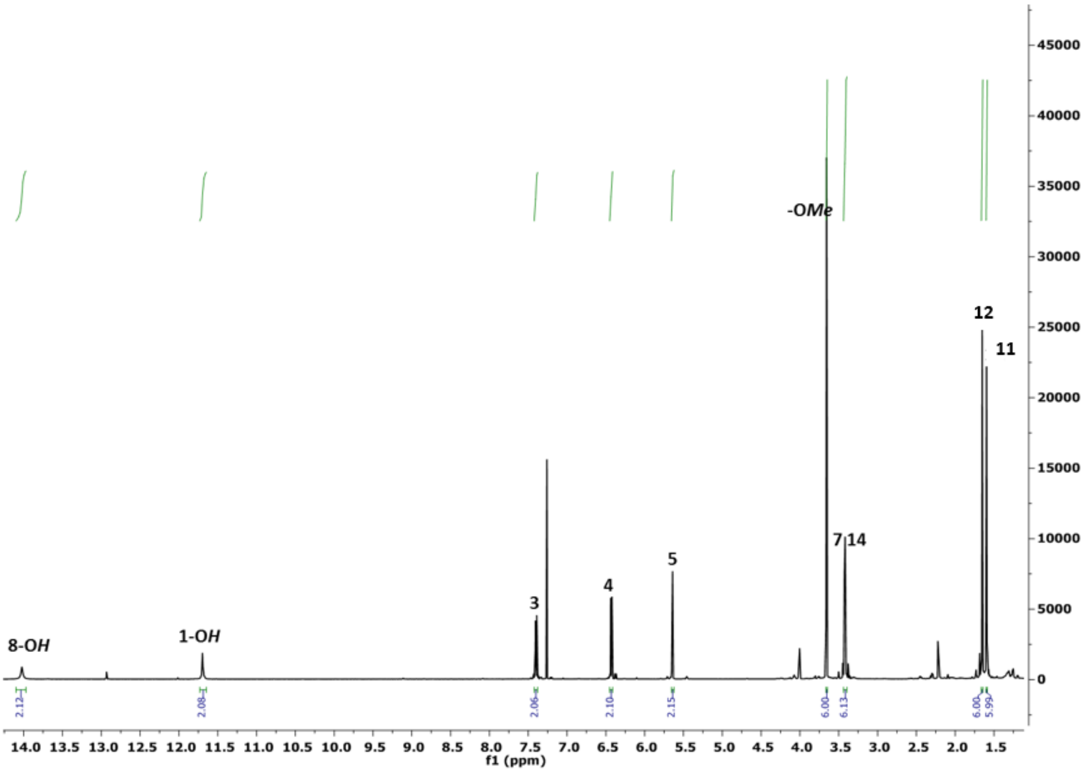


**Figure S16.** $^{1}$H NMR spectrum for cryptosporioptide **17** (500 MHz in $CDCl_3$).

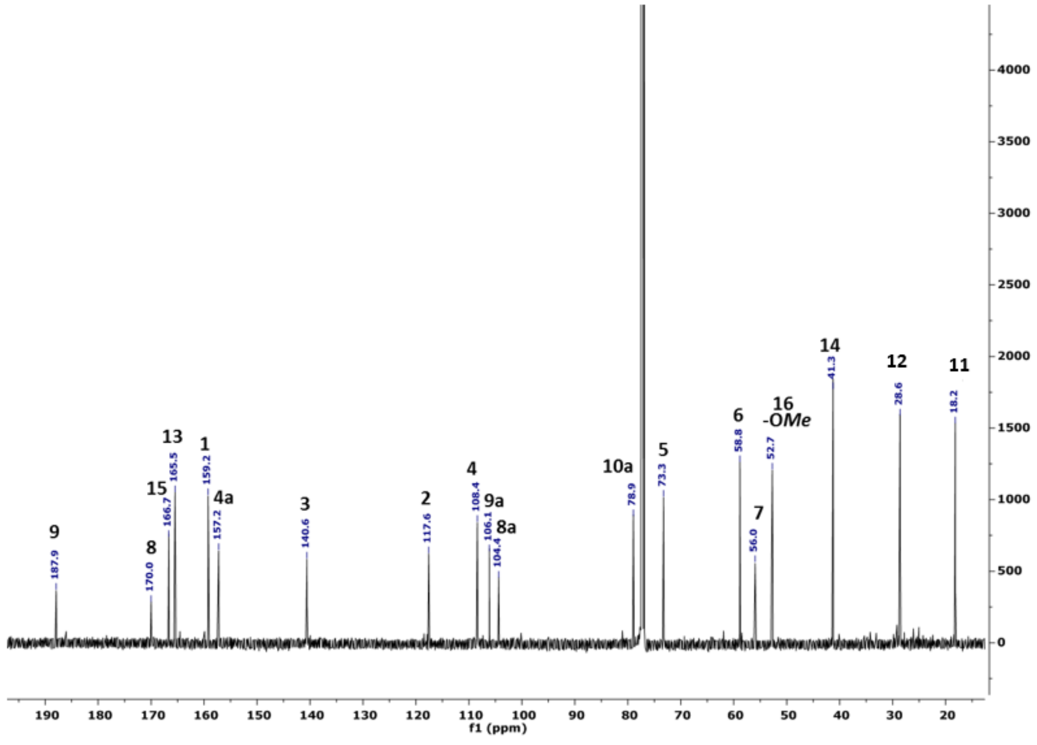


**Figure S17.** $^{13}$C NMR spectrum for cryptosporioptide **17** (126 MHz in $CDCl_3$).

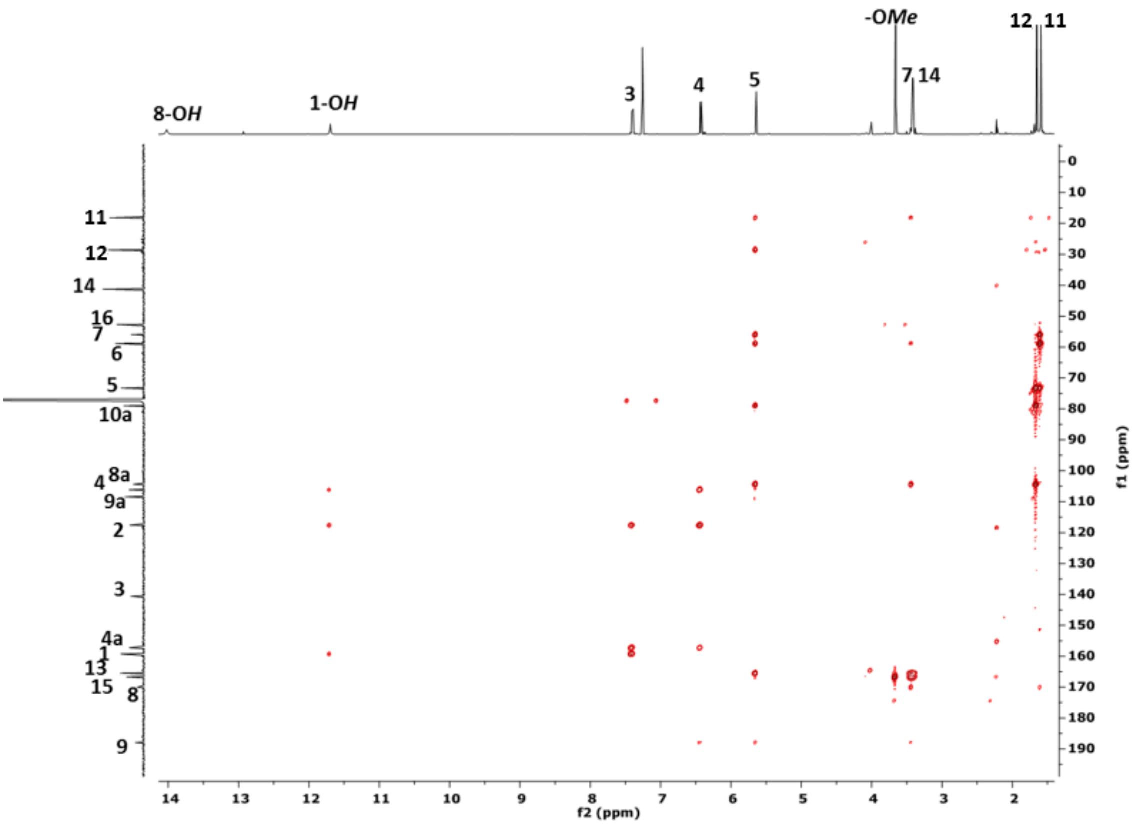


**Figure S18.** HMBC spectrum for cryptosporioptide **17** (500 MHz in $CDCl_3$).

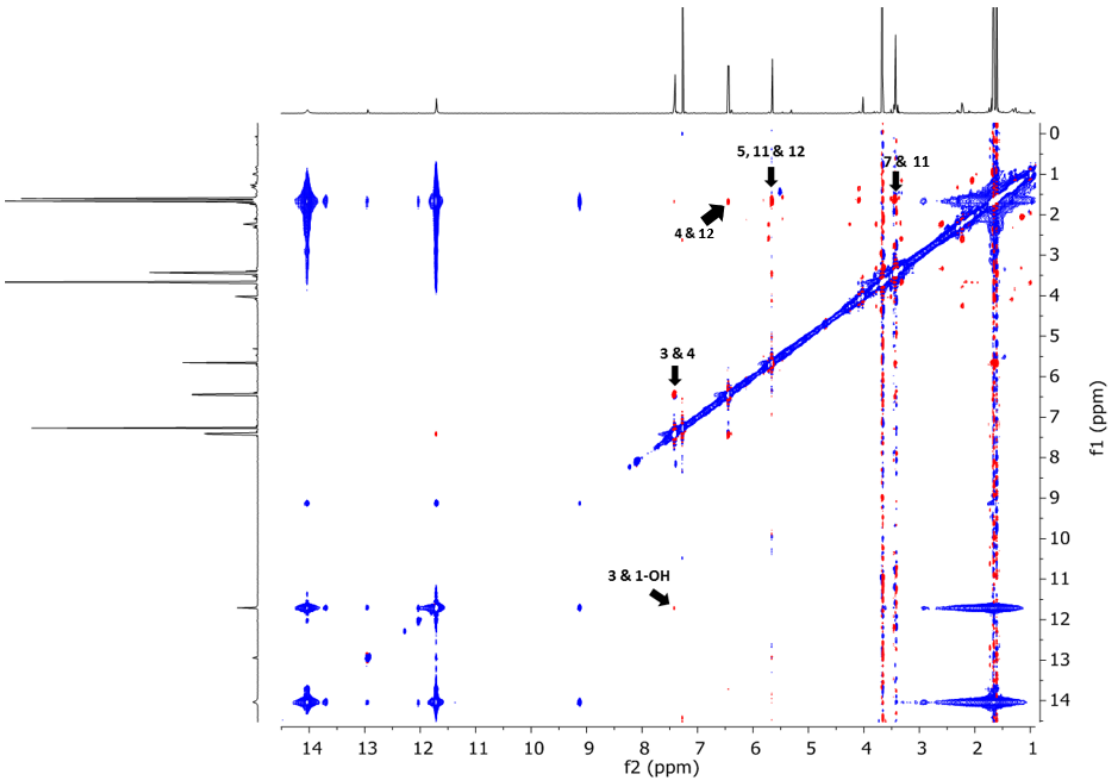


**Figure S19.** NOESY spectrum for cryptosporioptide **17** (500 MHz in $CDCl_3$); black arrows point to key NOE correlations.

**Cryptosporioptide B 18**

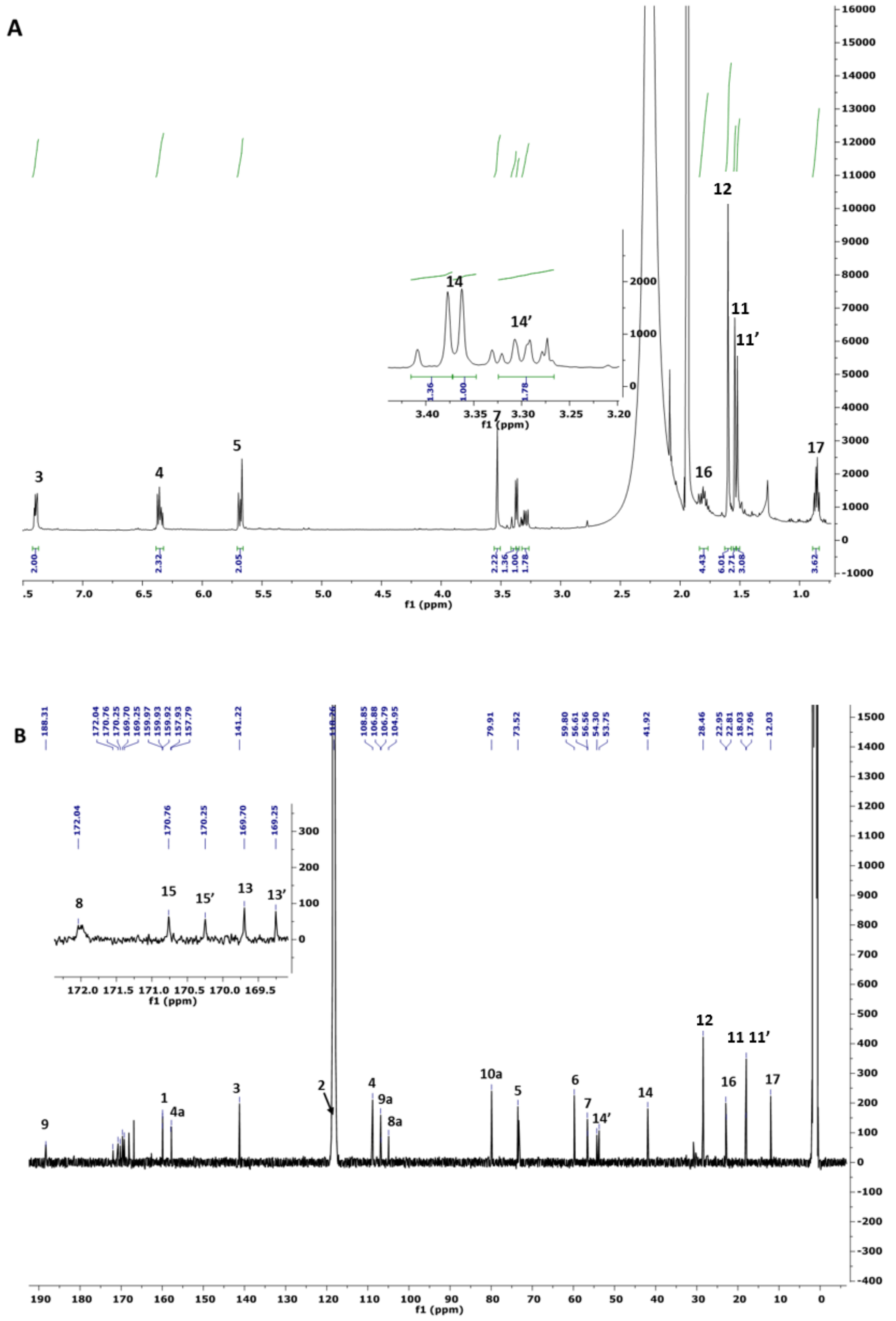


**Figure S20.** **A**,$^{1}$H NMR spectrum for cryptosporioptide B **18** (500 MHz in $CD_3CN$); **B**, $^{13}$C NMR spectrum for cryptosporioptide B **18** (126 MHz in $CD_3CN$).

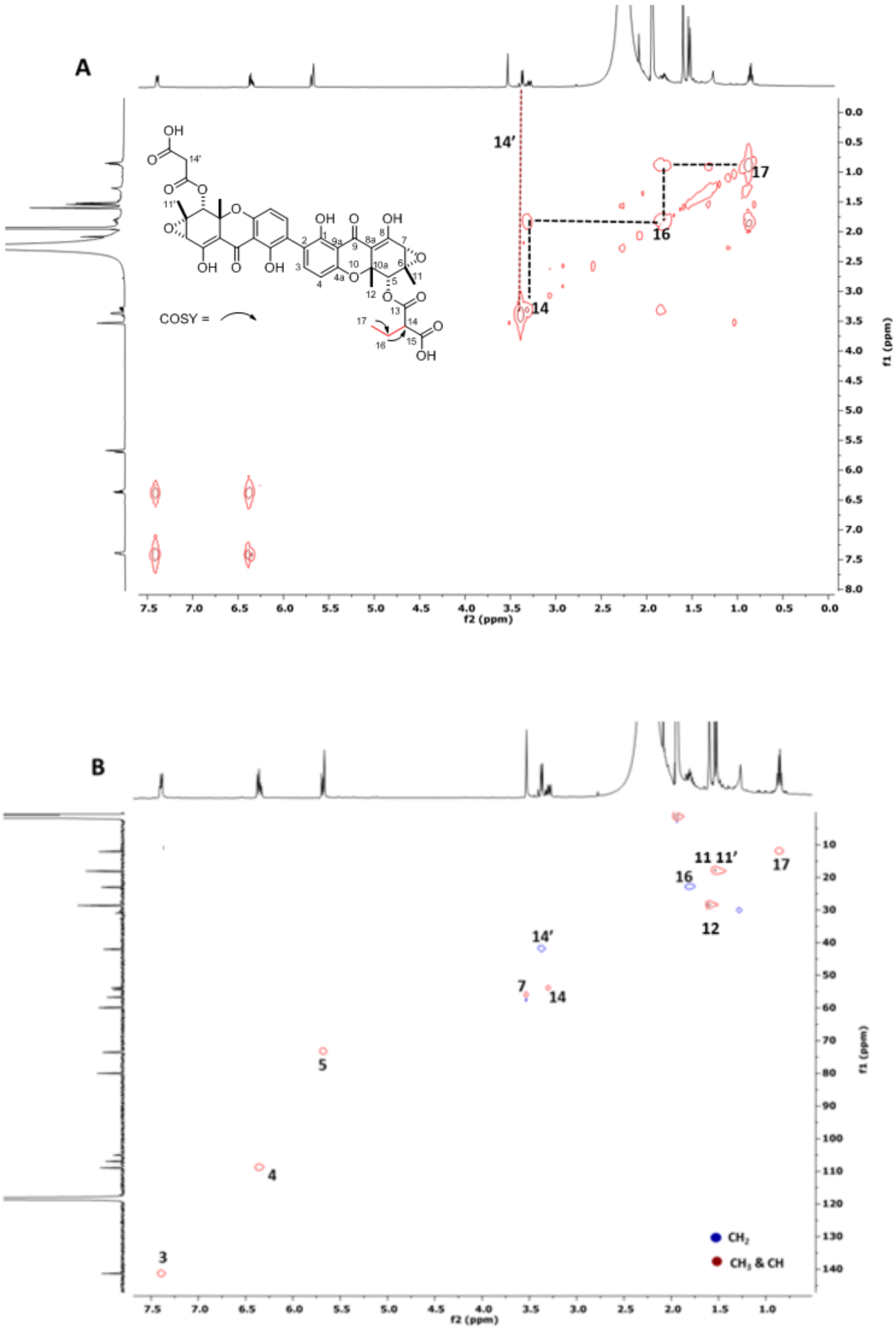


**Figure S21. A**, in the cryptosporioptide B **18** COSY spectrum correlation between the ethylmalonyl subunit can be observed (black dashed line) and no correlation for the H-14a (red dashed line); **B**, in the HSQC spectrum C-14 is clearly a -CH- group and signal for C-16 -$CH_2$- and C-17 -$CH_3$ can be observed, as well as C-14a which is a -$CH_2$-.

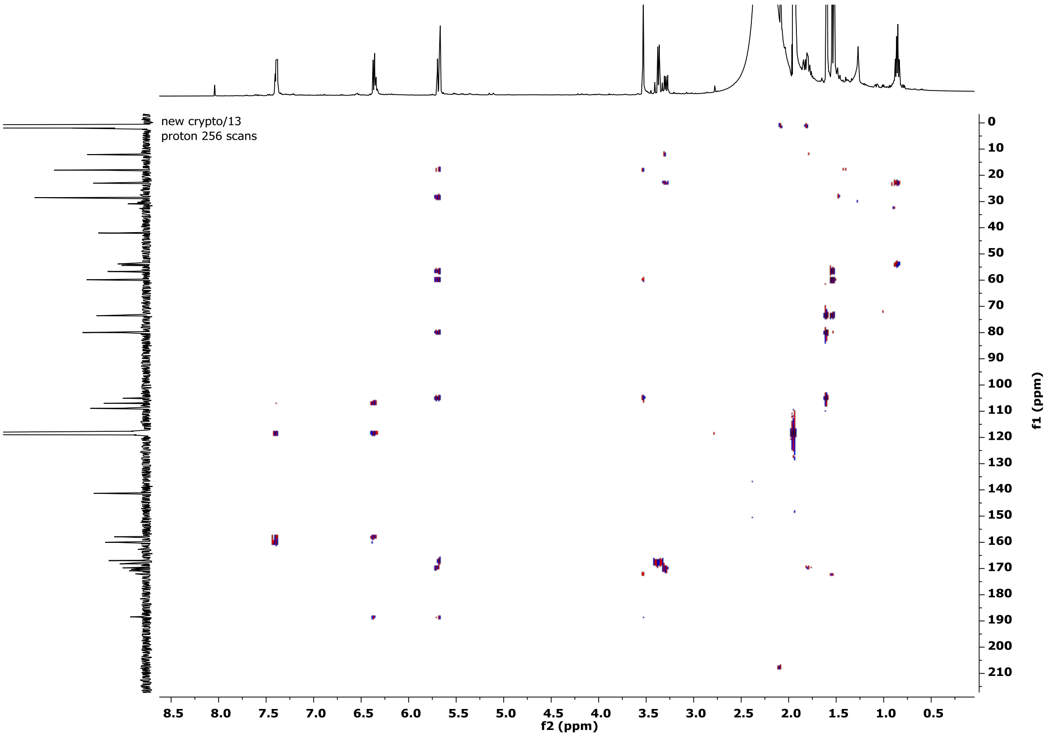


**Figure S22.** HMBC spectrum for cryptosporioptide B **18** (500 MHz in CDCl3).

## Cryptosporioptide C 19

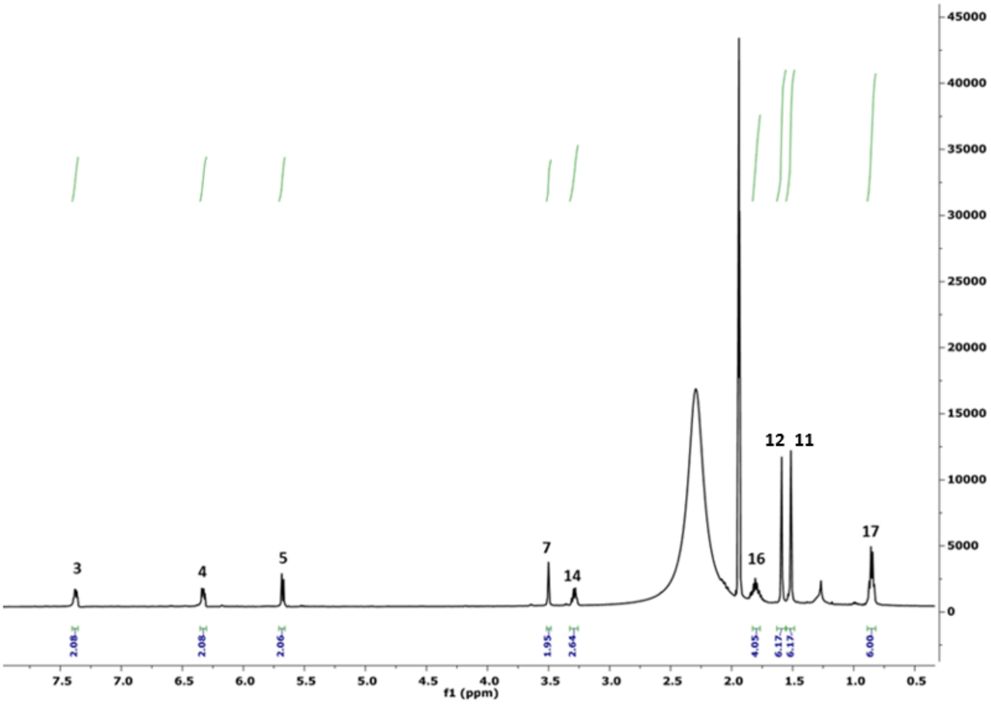


**Figure S23.** $^{1}$H NMR spectrum for cryptosporioptide C **19** (500 MHz in $CD_3CN$).

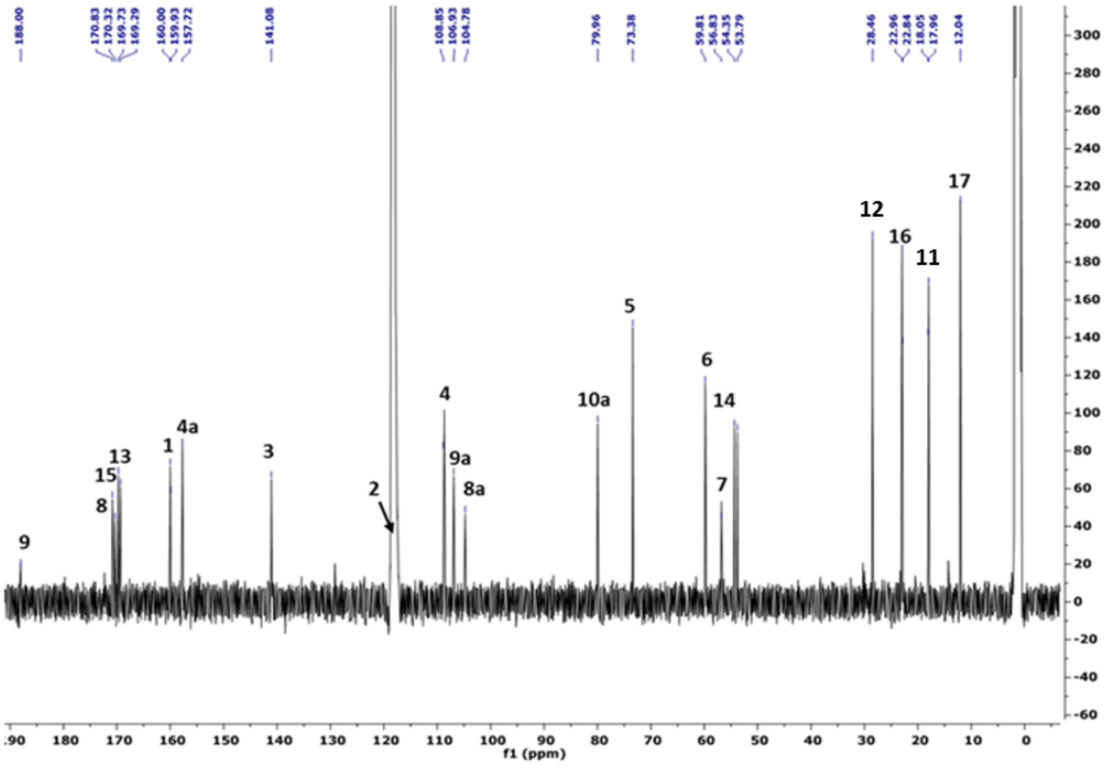

**Figure S24.** $^{13}$C NMR spectrum for cryptosporioptide C **19** (126 MHz in $CD_3CN$).

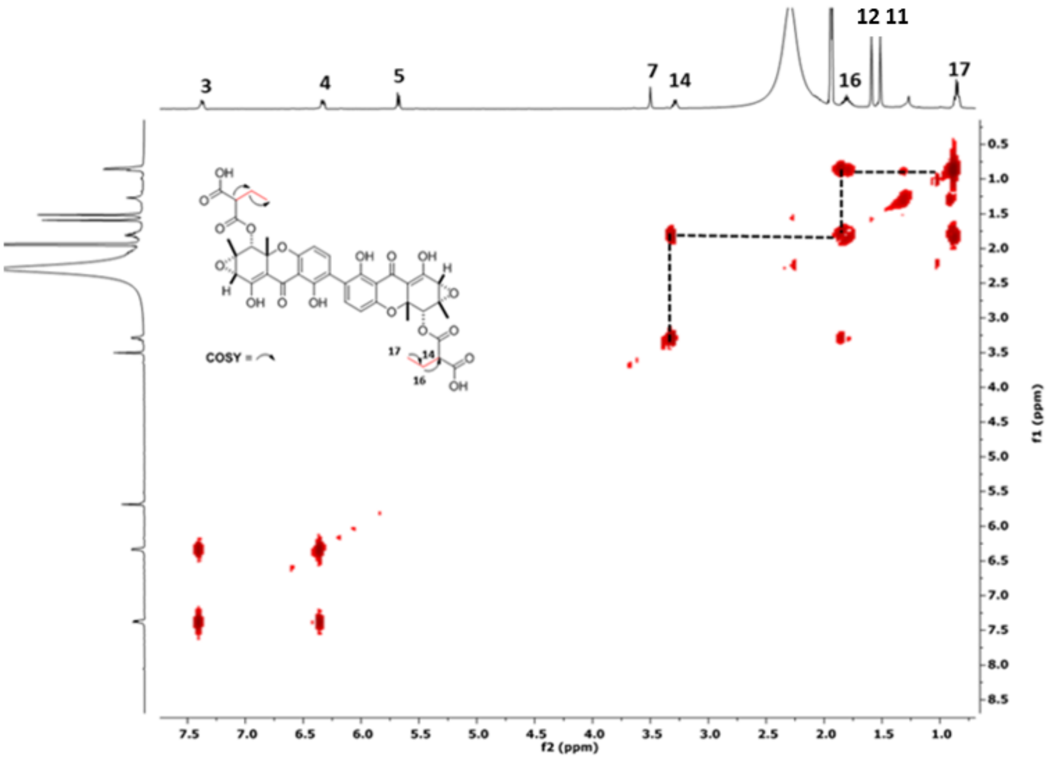

**Figure S25.** In the cryptosporioptide C **19** COSY spectrum correlation between the ethylmalonyl subunit can be observed (dashed line).

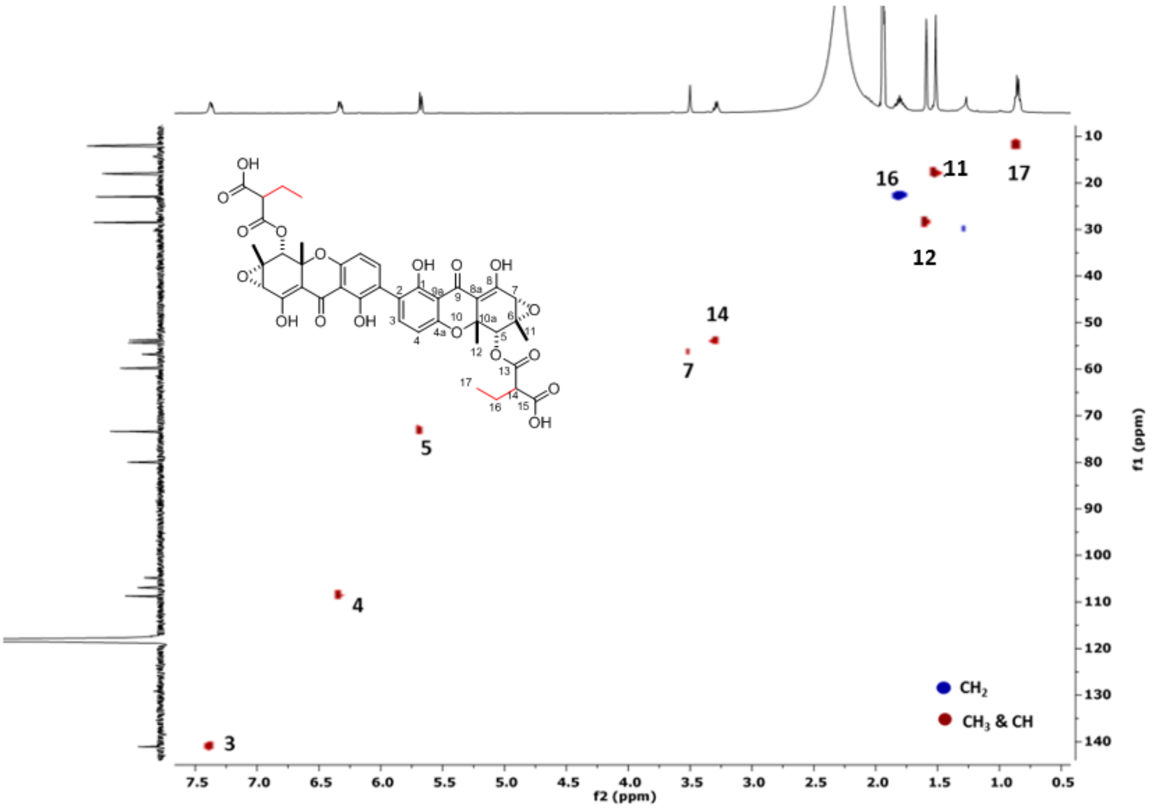


**Figure S26.** In the HSQC spectrum of **19** C-14 is clearly a -CH- group and signals for C-16 -$CH_2$- and C-17 -$CH_3$ can be observed.

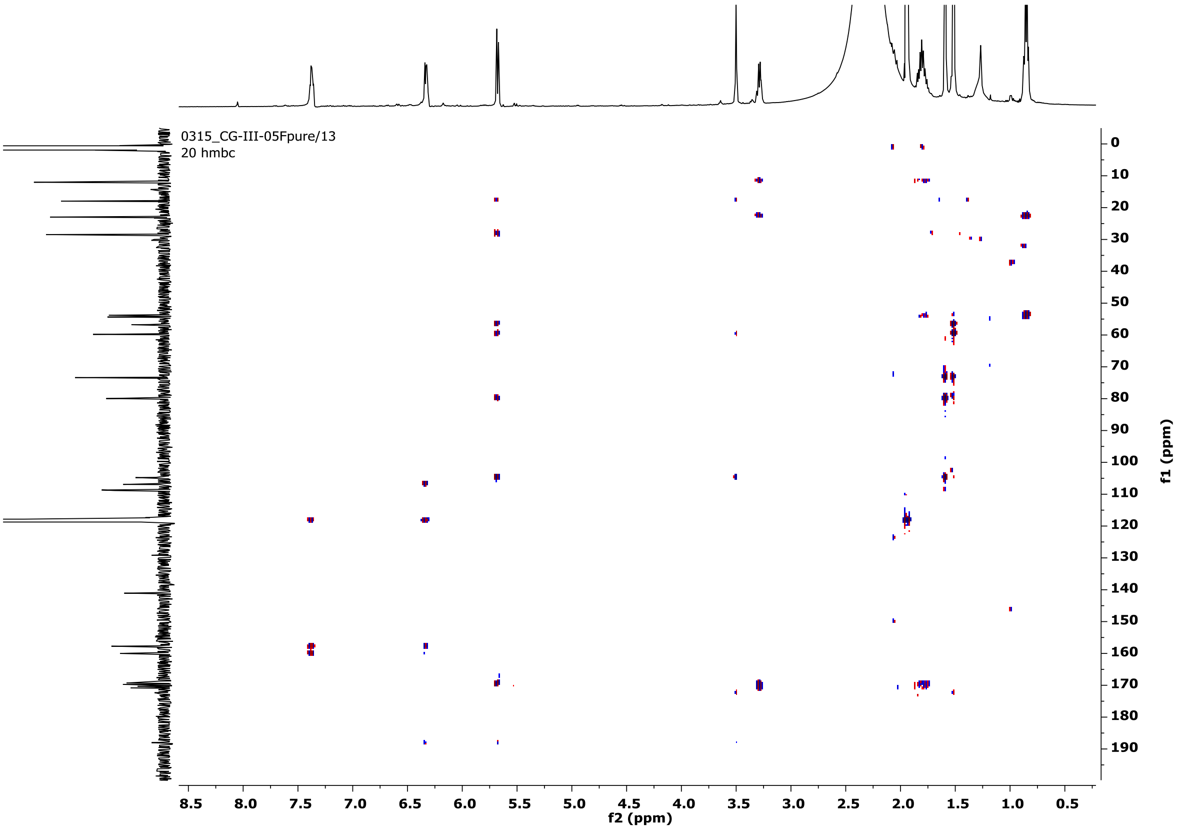


**Figure S27.** HMBC spectrum for cryptosporioptide C **19** (500 MHz in CDCl3).

## Cryptosporioptide monomer 21

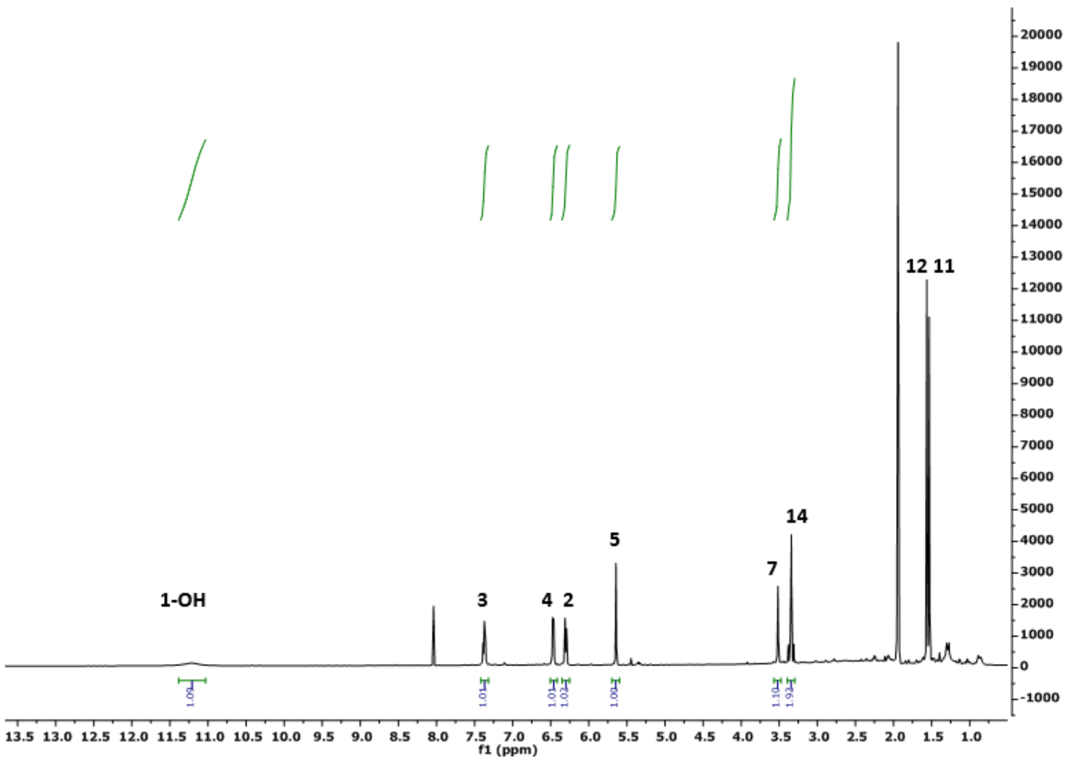


**Figure S28:** $^{1}$H NMR of cryptosporioptide monomer **21** (500 MHz in $CD_3CN$).

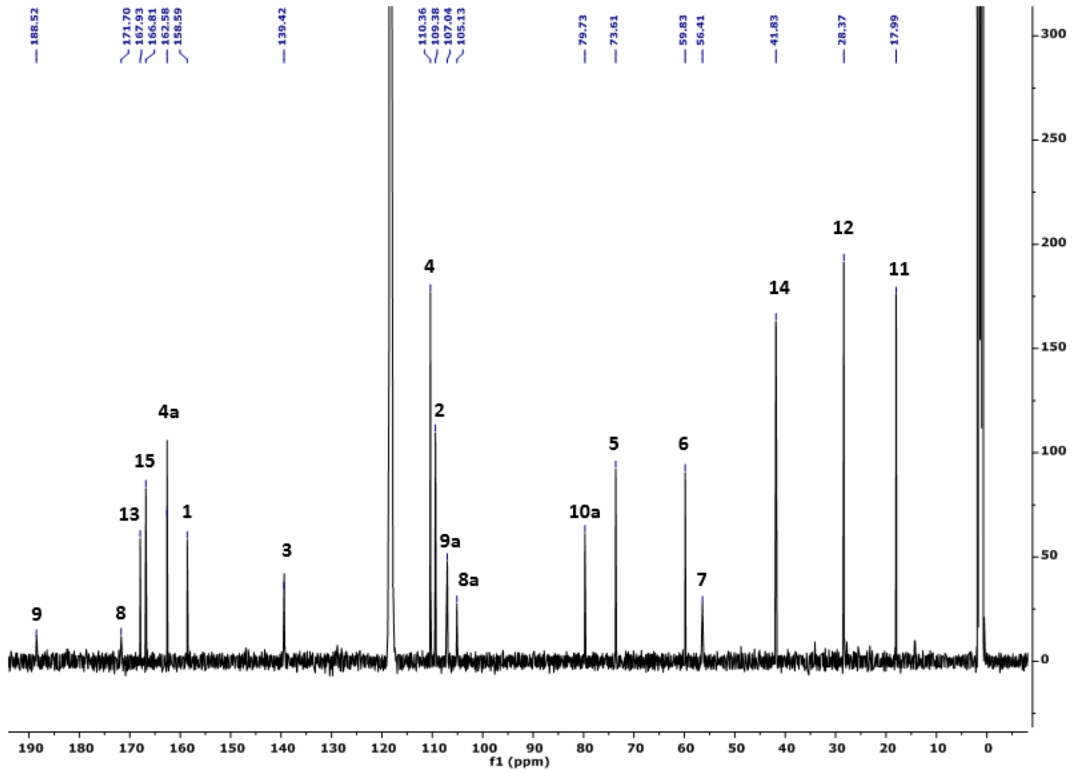


**Figure S29:** $^{13}$C NMR of cryptosporioptide monomer **21** (500 MHz in $CD_3CN$).

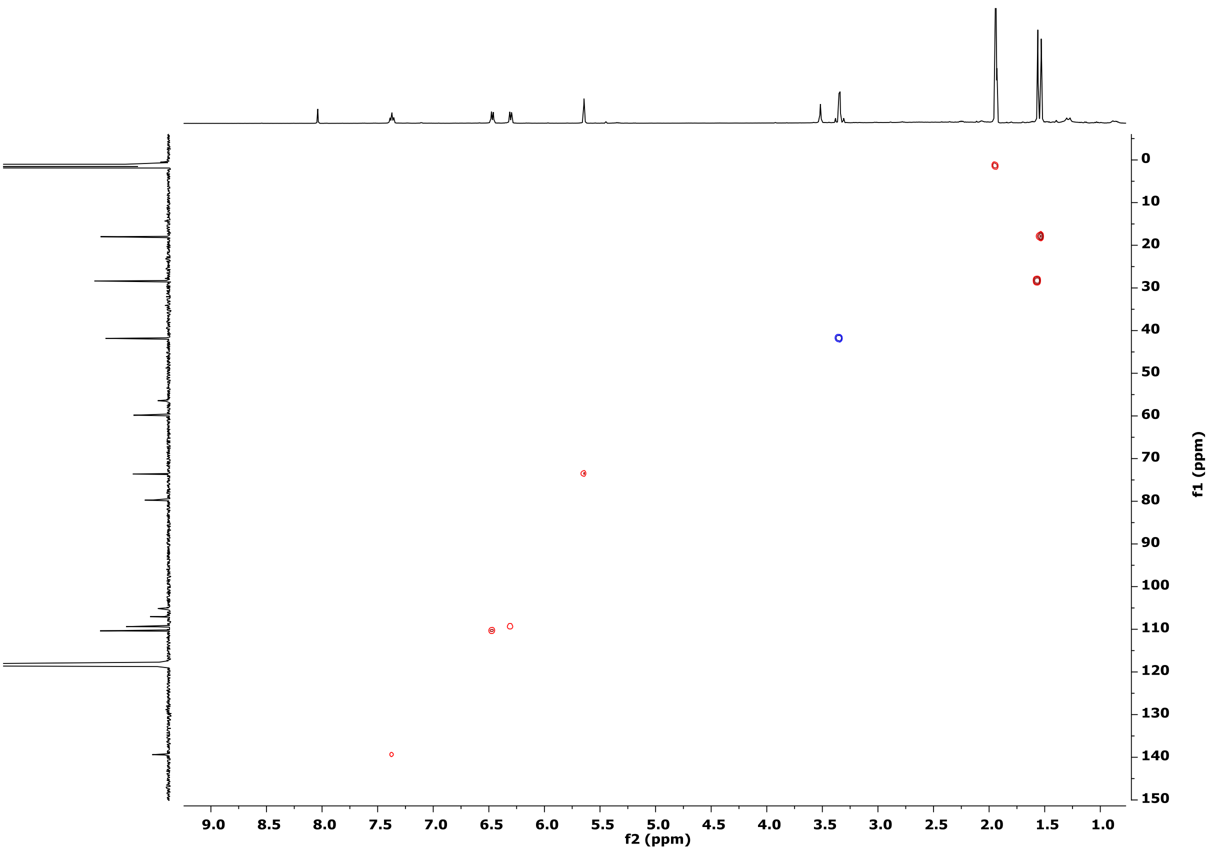


**Figure S30:** HSQC of cryptosporioptide monomer **21** (500 MHz in $CD_3OD$).

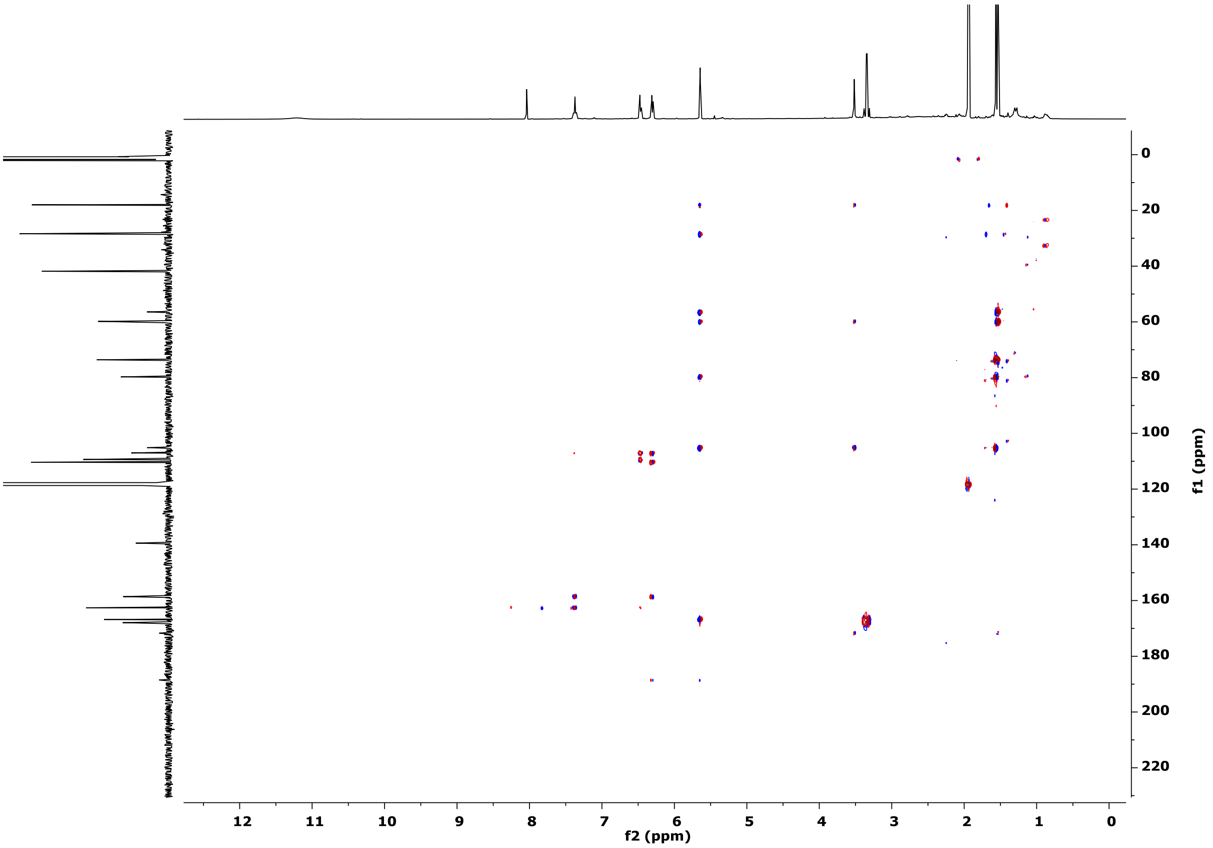


**Figure S31:** HSQC of cryptosporioptide monomer **21** (500 MHz in $CD_3CN$).

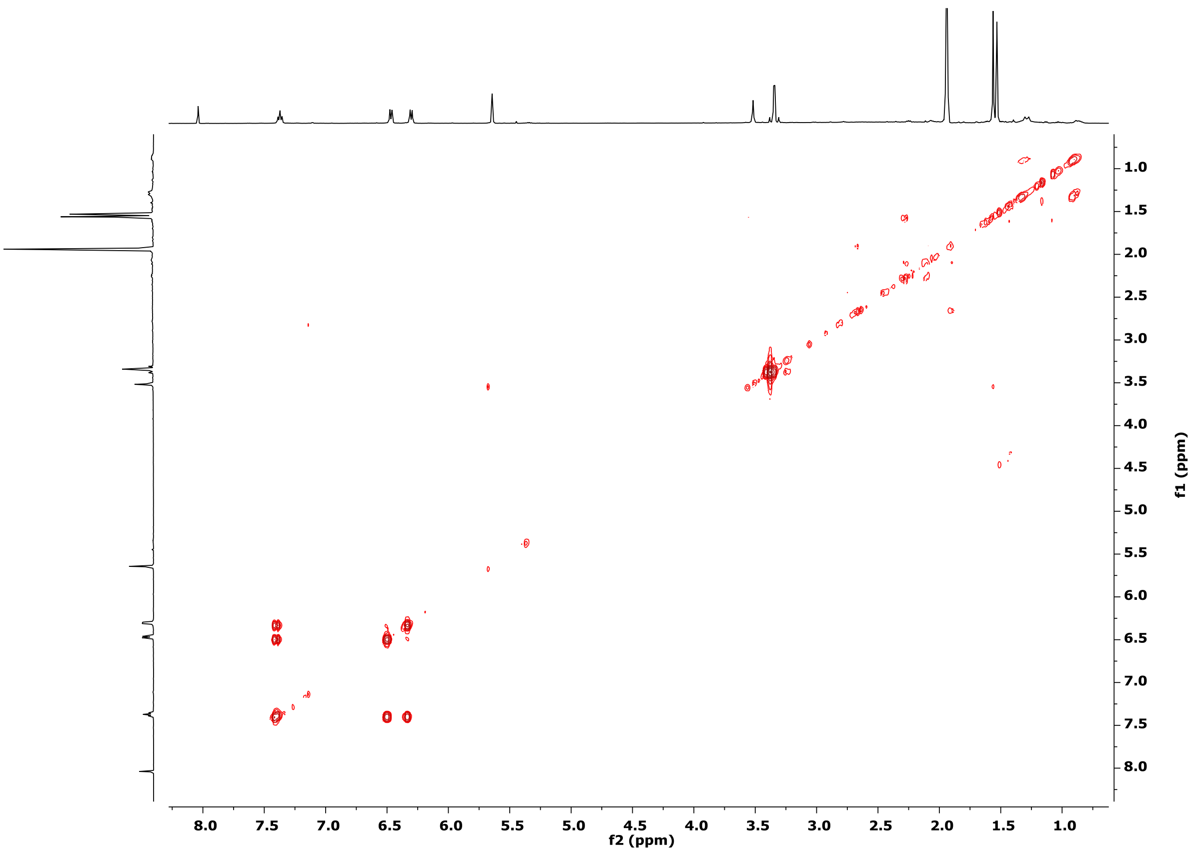


**Figure S32.** COSY of cryptosporioptide monomer **21 (**500 MHz in $CD_3CN$).

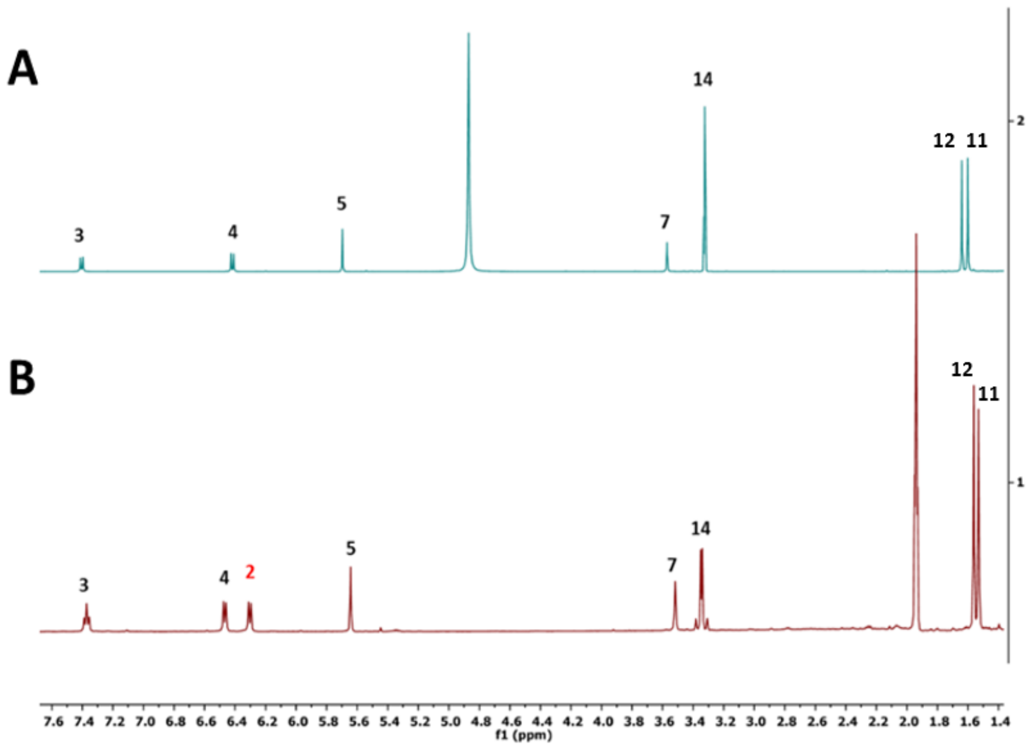


**Figure S33.** $^{1}$H NMR spectra: **A**, the cryptosporioptide A **16** (500 MHz $CD_3OD$); **B**, monomer **21** (500 MHz in $CD_3CN$). The additional aromatic proton is highlighted in red.

**Cryptosporioptide monomers 22a and 22b**

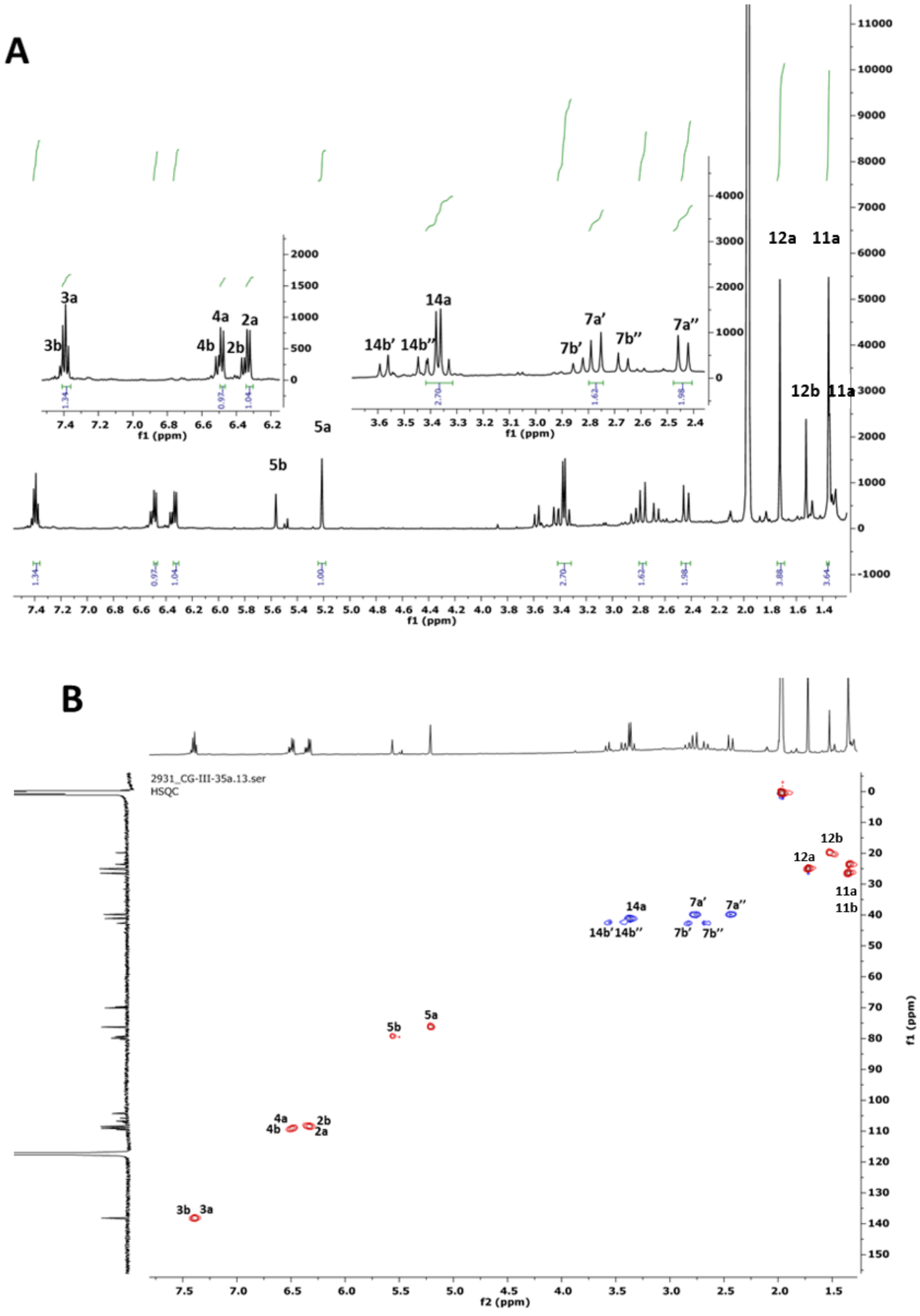


**Figure S34. A,** $^1$H NMR and **B**, HSQC of the two diastereoisomers **22a** and **22b** (500 MHz in $CD_3ON$).

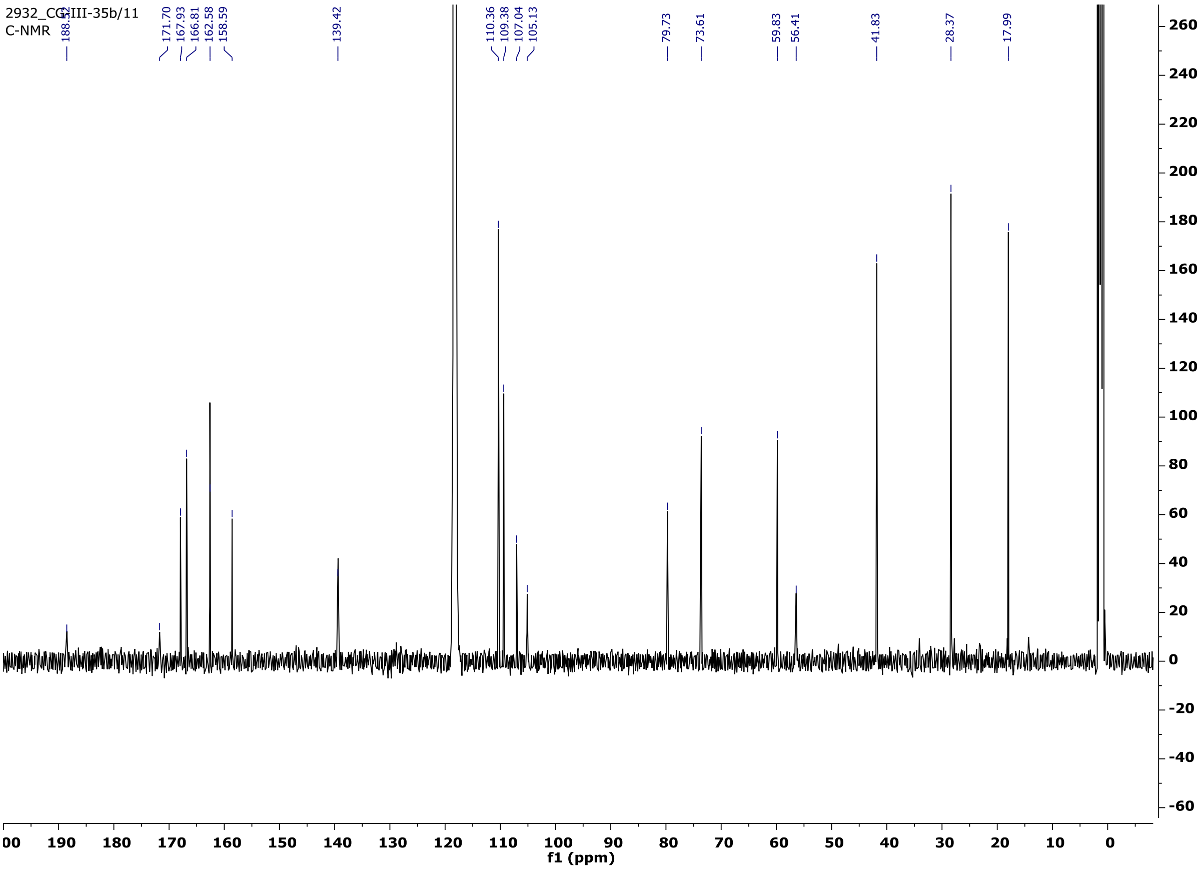


**Figure S35:** $^{13}$C NMR of cryptosporioptide monomers **22a** and **22b** **(**500 MHz in $CD_3ON$).

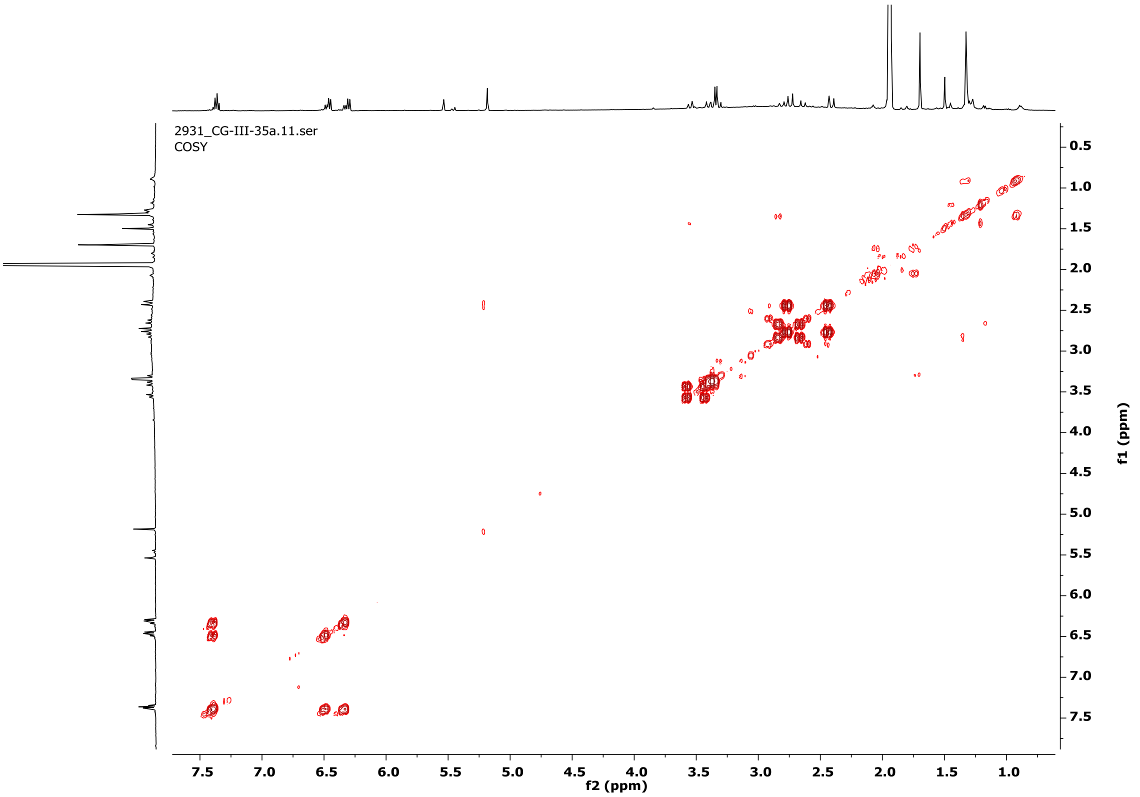


**Figure S36:** COSY of cryptosporioptide monomer **22a** and **22b** (500 MHz in $CD_3ON$).

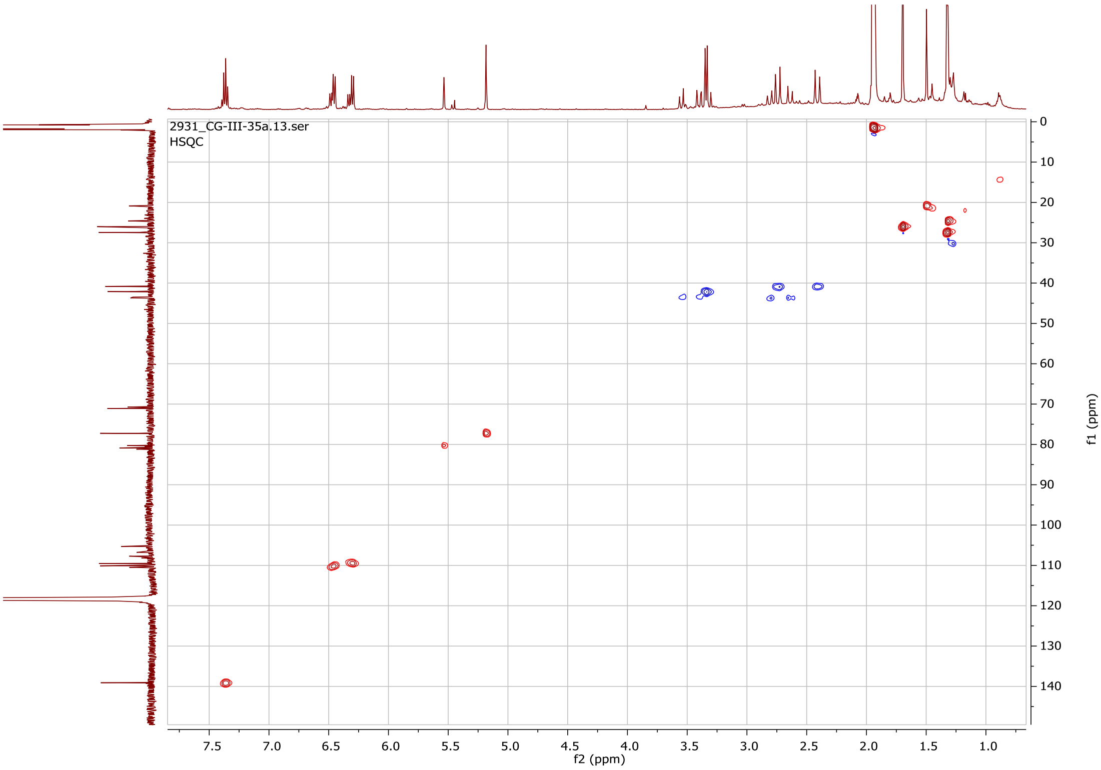


**Figure S37:** HSQC of cryptosporioptide monomer **22a** and **22b** (500 MHz in $CD_3CN$).

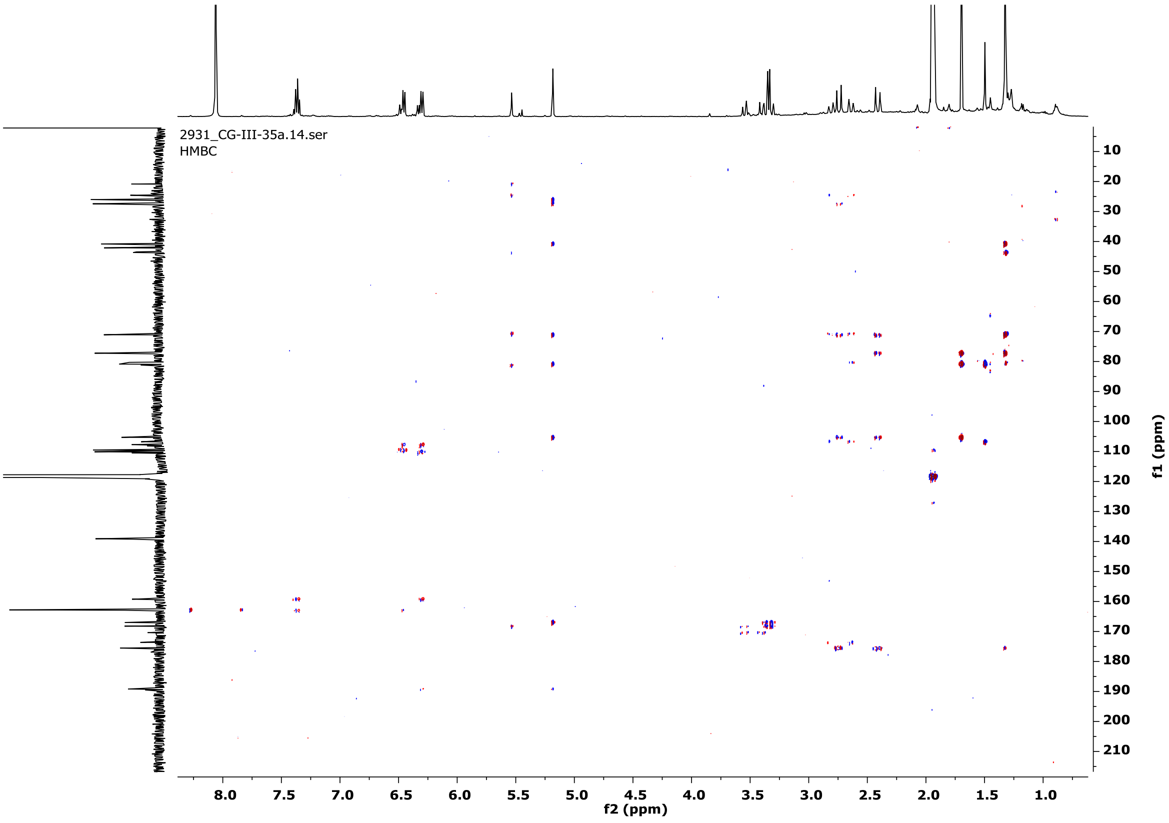


**Figure S38:** HMBC of cryptosporioptide monomer **22a** and **22b (**500 MHz in $CD_3CN$).

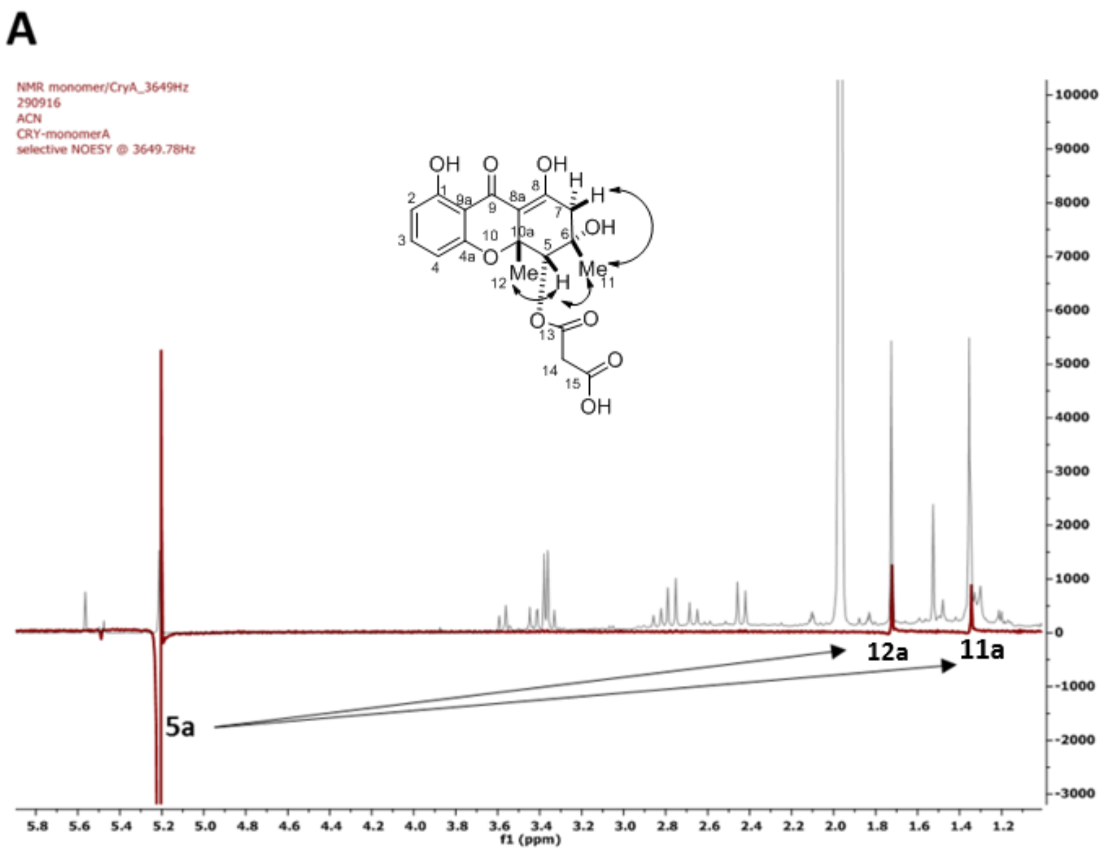


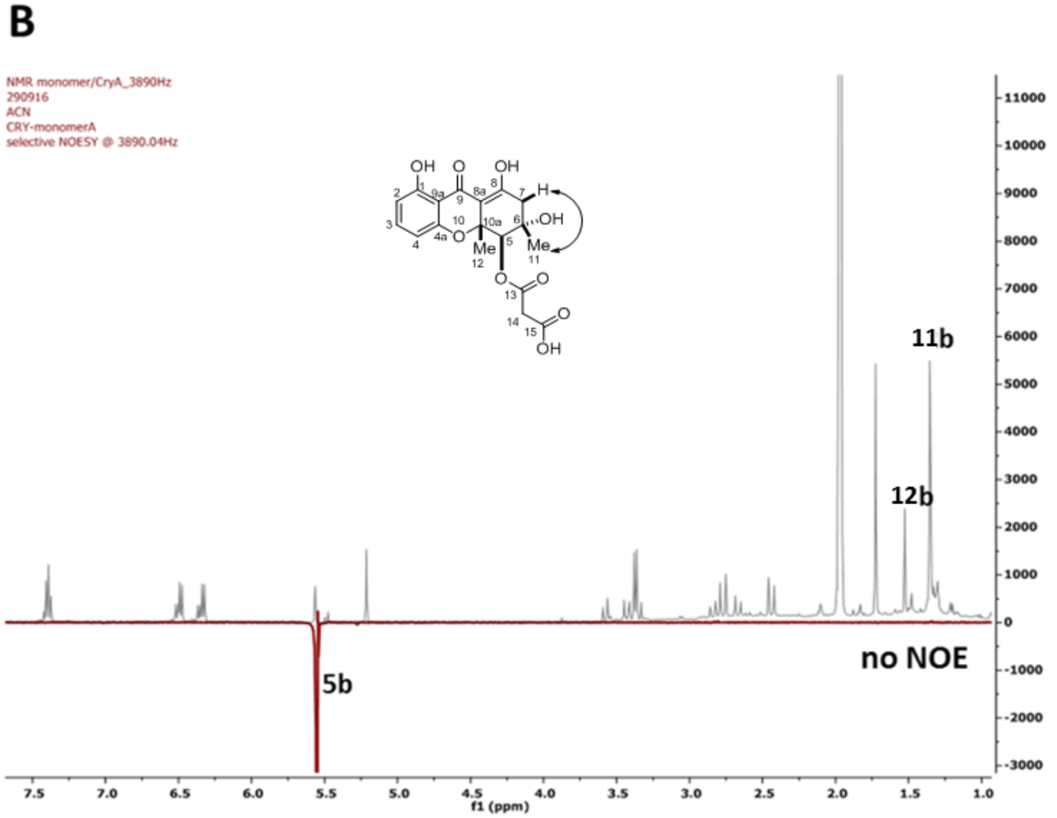


**Figure S39.** nOe correlations (700 MHz $CD_3CN$): **A**, to H-5a in **22a**; B, to H-5b in **22b**.

## References


1. T. B. Regueira, K. R. Kildegaard, B. G. Hansen, U. H. Mortensen, C. Hertweck and J. Nielsen, *Appl. Environ. Microbiol.*, 2011, **77**, 3035-3043.
2. J. Y. W. C. Nierman, N. D. Federova-Abrams, L. Losada, T. E. Cleveland, D. Bhatnagar, J. W. Bennett, R. Dean, and G. A. Payned, *Genome Announc.*, 2015, **3**.
3. C. D. Reeves, Z. H. Hu, R. Reid and J. T. Kealey, *Appl. Environ. Microbiol.*, 2008, **74**, 5121-5129.
4. W. Alfeel, S. S. Chirala and S. J. Wakil, *PNAS*, 1992, **89**, 4534-4538.
5. Y. M. Chiang, E. Szewczyk, A. D. Davidson, R. Entwistle, N. P. Keller, C. C. C. Wang and B. R. Oakley, *Appl. Environ. Microbiol.*, 2010, **76**, 2067-2074.
6. S. Hacquard, B. Kracher, K. Hiruma, P. C. Munch, R. Garrido-Oter, M. R. Thon, A. Weimann, U. Damm, J. F. Dallery, M. Hainaut, B. Henrissat, O. Lespinet, S. Sacristan, E. V. L. van Themaat, E. Kemen, A. C. McHardy, P. Schulze-Lefert and R. J. O'Connell, *Nat. Commun.*, 2016, **7**, 12.
7. J. J. Yu, P. K. Chang, K. C. Ehrlich, J. W. Cary, D. Bhatnagar, T. E. Cleveland, G. A. Payne, J. E. Linz, C. P. Woloshuk and J. W. Bennett, *Appl. Environ. Microbiol.*, 2004, **70**, 1253-1262.
8. E. Yang, W. N. Chow, G. Wang, P. C. Y. Woo, S. K. P. Lau, K. Y. Yuen, X. R. Lin and J. J. Cai, *Plos Genetics*, 2014, **10**.
9. Y.-M. Chiang, E. Szewczyk, A. D. Davidson, R. Entwistle, N. P. Keller, C. C. C. Wang and B. R. Oakley, *Appl. Environ. Microbiol.*, 2010, **76**, 2067-2074.
10. J. Davison, A. al Fahad, M. H. Cai, Z. S. Song, S. Y. Yehia, C. M. Lazarus, A. M. Bailey, T. J. Simpson and R. J. Cox, *PNAS*, 2012, **109**, 7642-7647.
11. M. R. Andersen, M. P. Salazar, P. J. Schaap, P. J. I. van de Vondervoort, D. Culley, J. Thykaer, J. C. Frisvad, K. F. Nielsen, R. Albang, K. Albermann, R. M. Berka, G. H. Braus, S. A. Braus-Stromeyer, L. M. Corrochano, Z. Y. Dai, P. W. M. van Dijck, G. Hofmann, L. L. Lasure, J. K. Magnuson, H. Menke, M. Meijer, S. L. Meijer, J. B. Nielsen, R. A. Samson, H. Stam, A. Tsang, J. M. van den Brink, A. Atkins, A. Aerts, H. Shapiro, J. Pangilinan, A. Salamov, Y. G. Lou, E. Lindquist, S. Lucas, J. Grimwood, I. V. Grigoriev, C. P. Kubicek, D. Martinez, N. van Peij, J. A. Roubos, J. Nielsen and S. E. Baker, *Genome Res.*, 2011, **21**, 885-897.
12. K. Throckmorton, F. Y. Lim, D. P. Kontoyiannis, W. F. Zheng and N. P. Keller, *EMI*, 2016, **18**, 246-259.
13. K. Throckmorton, P. Wiemann and N. P. Keller, *Toxins*, 2015, **7**, 3572-3607.
14. I. Fujii, Z. G. Chen, Y. Ebizuka and U. Sankawa, *Biochem. Internat.*, 1991, **25**, 1043-1049.
15. J. Sambrook, E. F. Fritsch and T. Maniatis, *Molecular cloning: A laboratory manual*, Cold Spring Harbor Laboratory Press, New York, 2004.
16. R. D. Gietz and R. A. Woods, *Methods Mol. Biol.*, 2006, **313**, 107-120.
17. L. M. Halo, J. W. Marshall, A. A. Yakasai, Z. Song, C. P. Butts, M. P. Crump, M. Heneghan, A. M. Bailey, T. J. Simpson, C. M. Lazarus and R. J. Cox, *Chembiochem*, 2008, **9**, 585-594.
18. E. B. Chain and G. Mellows, *J. Chem. Soc., Perkin Trans. 1*, 1977, 294-309.
19. A. Francais, A. Leyva-Perez, G. Etxebarria-Jardi, J. Pena and S. V. Ley, *Chem. Eur. J.*, 2011, **17**, 329-343.
20. J. A. Anderson, *Phytochem.*, 1985, **25**, 103-106.
21. D. Abdissa, G. Geleta, K. Bacha and N. Abdissa, *Plos One*, 2017, **12**.
